# Double-Blind, Randomized, Placebo-Controlled Phase III Clinical Trial to Evaluate the Efficacy and Safety of treating Healthcare Professionals with the Adsorbed COVID-19 (Inactivated) Vaccine Manufactured by Sinovac – PROFISCOV: A structured summary of a study protocol for a randomised controlled trial

**DOI:** 10.1186/s13063-020-04775-4

**Published:** 2020-10-15

**Authors:** Ricardo Palacios, Elizabeth González Patiño, Roberta de Oliveira Piorelli, Monica Tilli Reis Pessoa Conde, Ana Paula Batista, Gang Zeng, Qianqian Xin, Esper G. Kallas, Jorge Flores, Christian F. Ockenhouse, Christopher Gast

**Affiliations:** 1 Center for Clinical Trials and Pharmacovigilance, Instituto Butantan, São Paulo, Brazil; 2 Sinovac Life Sciences, Beijing, China; 3 Department of Infectious and Parasitic Diseases, School of Medicine, University of São Paulo, São Paulo, Brazil; 4 PATH US, Washington DC, USA; 5 PATH, Seattle, WA USA

**Keywords:** COVID-19, Randomised controlled trial, protocol, Inactivated Vaccines, Phase III Clinical Trial, COVID-19 Vaccine, Brazil

## Abstract

**Objectives:**

To evaluate the efficacy of two doses of the adsorbed vaccine COVID-19 (inactivated) produced by Sinovac in symptomatic individuals, with virological confirmation of COVID-19, two weeks after the completion of the two-dose vaccination regimen, aged 18 years or older who work as health professionals providing care to patients with possible or confirmed COVID-19.

To describe the occurrence of adverse reactions associated with the administration of each of two doses of the adsorbed vaccine COVID-19 (inactivated) produced by Sinovac up to one week after vaccination in Adults (18-59 years of age) and Elderly (60 years of age or more).

**Trial design:**

This is a Phase III, randomized, multicenter, endpoint driven, double-blind, placebo-controlled clinical trial to assess the efficacy and safety of the adsorbed vaccine COVID-19 (inactivated) produced by Sinovac.

The adsorbed vaccine COVID-19 (inactivated) produced by Sinovac (product under investigation) will be compared to placebo. Voluntary participants will be randomized to receive two intramuscular doses of the investigational product or the placebo, in a 1: 1 ratio, stratified by age group (18 to 59 years and 60 years or more) and will be monitored for one year by active surveillance of COVID-19. Two databases will be established according to the age groups: one for adults (18-59 years) and one for the elderly (60 years of age or older).

The threshold to consider the vaccine efficacious will be to reach a protection level of at least 50%, as proposed by the World Health Organization and the FDA. Success in this criterion will be defined by sequential monitoring with adjustment of the lower limit of the 95% confidence interval above 30% for the primary efficacy endpoint.

**Participants:**

Healthy participants and / or participants with clinically controlled disease, of both genders, 18 years of age or older, working as health professionals performing care in units specialized in direct contact with people with possible or confirmed cases of COVID-19. Participation of pregnant women and those who are breastfeeding, as well as those intending to become pregnant within three months after vaccination will not be allowed. Participants will only be included after signing the voluntary Informed Consent Form and ensuring they undergo screening evaluation and conform to all the inclusion and exclusion criteria. All the clinical sites are located in Brazil.

**Intervention and comparator:**

Experimental intervention: The vaccine was manufactured by Sinovac Life Sciences (Beijing, China) and contains 3 μg/0.5 mL (equivalent to 600 SU per dose) of inactivated SARS-CoV-2 virus, and aluminium hydroxide as adjuvant.

Control comparator: The placebo contains aluminium hydroxide in a 0.5 mL solution

The schedule of both, experimental intervention and placebo is two 0.5 mL doses IM (deltoid) with a two week interval.

**Main outcomes:**

The primary efficacy endpoint is the incidence of symptomatic cases of virologically confirmed COVID-19 two weeks after the second vaccination. The virological diagnosis will be confirmed by detection of SARS-CoV-2 nucleic acid in a clinical sample.

The primary safety endpoint is the frequency of solicited and unsolicited local and systemic adverse reactions during the period of one week after vaccination according to age group in adult (18-59 years old) and elder (60 years of age or older) subjects. Adverse reactions are defined as adverse events that have a reasonable causal relationship to vaccination.

**Randomisation:**

There will be two randomization lists, one for each age group, based on the investigational products to be administered, *i.e*., vaccine or placebo at a 1: 1 ratio. Each randomization list will be made to include up to 11,800 (18-59 year-old) adults, and 1,260 elderly (60 y-o and older) participants, the maximum number of participants needed per age group. An electronic central randomization system will be used to designate the investigational product that each participant must receive.

**Blinding (masking):**

This trial is designed as a double-blind study to avoid introducing bias in the evaluation of efficacy, safety and immunogenicity. The clinical care team, the professionals responsible for the vaccination and the participants will not know which investigational product will be administered. Only pharmacists or nurses in the study who are responsible for the randomization, separation and blinding of the investigational product will have access to unblinded information. The sponsor's operational team will also remain blind.

**Numbers to be randomised (sample size):**

The total number of participants needed to evaluate efficacy, 13,060 participants, satisfies the needed sample size calculated to evaluate safety. Therefore, the total number obtained for efficacy will be the number retained for the study. Up to 13,060 participants are expected to enter the study, with up to 11,800 participants aged 18 to 59 years and 1,260 elderly participants aged 60 and over. Half of the participants of each group will receive the experimental vaccine and half of them will receive the placebo. The recruitment of participants may be modified as recommended by the Data Safety Monitoring Committee at time of the interim unblinded analysis or blind assessment of the COVID-19 attack rate during the study.

**Trial Status:**

Protocol version 2.0 – 24-Aug-2020. Recruitment started on July 21^st^, 2020. The recruitment is expected to conclude in October 2020.

**Trial registration:**

ClinicalTrials.gov Identifier: NCT0445659. Registry on 2 July 2020

**Full protocol:**

The full protocol is attached as an additional file, accessible from the Trials website (Additional file 1). In the interest in expediting dissemination of this material, the familiar formatting has been eliminated; this Letter serves as a summary of the key elements of the full protocol.

## Supplementary information

**Additional file 1.** Full Study Protocol.

## Data Availability

Undecided

