# Supplementary material for: Double-Blind, Randomized, Placebo-Controlled Phase III Clinical Trial to Evaluate the Efficacy and Safety of treating Healthcare Professionals with the Adsorbed COVID-19 (Inactivated) Vaccine Manufactured by Sinovac – PROFISCOV: A structured summary of a study protocol for a randomised controlled trial
Source: Trials. 2020 Oct 15;21:853. doi: 10.1186/s13063-020-04775-4 (PMC7558252; doi:10.1186/s13063-020-04775-4)
Supplement: Supplementary file 1 — Additional file 1. Full Study Protocol. [file 13063_2020_4775_MOESM1_ESM.pdf]

COV-02-IB

PROFISCOV Study Protocol

Original: Version 2.0 24-Aug-2020 – Portuguese

Translation: Version 2.0 24-Aug-2020 – English

INSTITUTO BUTANTAN
A serviço da vida

Protocolo de pesquisa clínica	COV-02-IB<br><br>Ensaio Clínico Fase III duplo-cego, randomizado, controlado com placebo para Avaliação de Eficácia e Segurança em Profissionais da Saúde da Vacina adsorvida COVID-19 (inativada) produzida pela Sinovac
Acrônimo do Estudo	PROFISCOV

Versão: 2.0 de 24 de agosto de 2020

CONFIDENCIAL

As informações confidenciais contidas neste documento são fornecidas para você como um investigador ou membro de uma equipe de pesquisa do estudo ou revisor dentro do processo de aprovação do estudo. A sua aceitação deste documento se caracteriza em um acordo no qual você se compromete a não revelar as informações aqui contidas para outras partes sem a autorização por escrito do Instituto Butantan ou dos seus representantes autorizados.

Instituto Butantan
Av. Vital Brasil, 1500, Butantã
São Paulo – SP 05503-900 – Brasil
Fone: (11) 3723 2121 – Fax: (11) 2627 9375

CONTEÚDO

1 INFORMAÇÃO ADMINISTRATIVA 8

1.1 Título do protocolo 8

1.2 Informação de registro 8

1.3 Histórico de versões 11

1.4 Financiamento 11

1.5 Responsáveis pelo protocolo 11

1.5.1 Contribuições para o protocolo 11

1.5.2 Papel do patrocinador e financiadores 12

1.5.3 Comitês do estudo 12

2 INTRODUÇÃO 12

2.1 Revisão da literatura/Estado atual do conhecimento sobre o tema do projeto 12

2.1.1 Infecções por coronavírus em seres humanos 12

2.1.2 A emergência do SARS-CoV-2 14

2.1.3 Apresentação clínica 15

2.1.4 Epidemiologia da COVID-19 16

2.1.5 Resposta imune contra o vírus SARS-CoV-2 17

2.1.6 Desenvolvimento de vacinas contra a COVID-19 18

2.1.7 Desenvolvimento da vacina adsorvida COVID-19 (inativada) produzida pela Sinovac (CoronaVac) 18

2.2 Objetivos 23

2.2.1 Objetivos primários 23

2.2.2 Objetivos secundários 23

2.2.3 Objetivos exploratórios 24

2.3 Desenho geral do estudo 25

3 MÉTODOS 26

3.1 Centros de estudo 26

3.2 Participantes 26

3.2.1 Critérios de inclusão 26

3.2.2 Critérios de exclusão 26

3.3 Desfechos 28

3.3.1 Desfechos primários 28

3.3.2 Desfechos secundários 28

3.3.3 Desfechos exploratórios 29

3.4 Intervenções 30

3.4.1 Composição final e formulações da vacina adsorvida covid-19 (inativada) produzida pela Sinovac 30

3.4.2 Intervenções ou regimes de tratamentos concomitantes permitidos, não recomendados e proibidos 31

3.4.3 Risco de gravidez para participantes do sexo feminino 32

3.4.4 Procedimentos para verificar a adesão ao tratamento 33

3.5 Procedimentos para o participante 33

3.5.1 Acompanhamento dos participantes 33

3.5.2 Procedimentos para a realização do estudo 33

3.5.3 Descrição dos procedimentos do estudo por visita 33

3.5.4 Vacinação 36

3.5.5 acompanhamento de eventos adversos nas duas semanas após a vacinação e uso do “diário do participante” 36

3.5.6 Procedimentos em caso de febre e suspeita de COVID-19 37

3.5.7 Procedimento em caso de perda de visita ou contato 38

3.6 Diagrama de procedimentos do estudo 38

3.7 Critérios e procedimentos para suspensão ou exclusão do participante 39

3.8 Tamanho amostral ... 40

3.8.1 Cálculo de tamanho amostral e poder estatístico ... 40

3.8.2 Número estimado de participantes ... 42

3.9 População fonte do estudo e área de recrutamento ... 42

3.10 Alocação de participantes ... 43

3.10.1 Geração da sequência de alocação ... 43

3.10.2 Mecanismo de ocultamento da alocação ... 43

3.10.3 Implementação da geração da sequência de alocação ... 43

3.11 Procedimentos de cegamento ... 44

3.11.1 Descrição de quem será cego no estudo ... 44

3.11.2 Procedimentos para a quebra do cegamento ... 44

3.12 Coleta de dados ... 44

3.13 programa de análise e processamento de dados ... 45

3.14 Plano de análise de dados, incluindo metodologia estatística ... 45

3.14.1 Definições de populações a serem analisadas ... 45

3.14.2 Metodologia Descritiva ... 46

3.14.3 Análise primário de eficácia ... 47

3.14.4 Análises de segurança ... 47

3.14.5 Análises de imunogenicidade ... 48

3.14.6 gerenciamento de dados faltantes/ausentes ... 49

3.15 Monitoramento de dados do estudo ... 49

3.15.1 Comitê de monitoramento de dados e segurança ... 49

3.15.2 Análises e revisão interina ... 50

3.15.3 Critérios para suspensão ou interrupção do estudo ... 51

3.16 Avaliação da segurança ... 51

3.16.1 Definições ... 51

3.16.2 Monitoramento dos eventos adversos 52

3.16.3 Classificação da intensidade 53

3.16.4 Classificação da relação causal 56

3.16.5 Identificação e relato dos eventos adversos 57

3.16.6 Período de avaliação da segurança 59

3.16.7 Definições de caso de COVID-19 60

3.16.8 Resposta a achados novos ou inesperados e alterações no ambiente do estudo 62

3.17 Garantia/controle de qualidade de dados 62

3.17.1 Monitoramento clínico 62

3.17.2 Acesso aos documentos fonte 63

4 ÉTICA E PUBLICAÇÕES 63

4.1 Aprovações para a realização da pesquisa 63

4.1.1 Declaração do marco regulatório do estudo 63

4.1.2 Aprovações do estudo 63

4.2 Emendas ao protocolo 64

4.3 Consentimento livre e esclarecido 64

4.3.1 Procedimentos para a implementação e documentação do consentimento livre e esclarecido 64

4.3.2 Armazenamento de amostras biológicas e uso de biorrepositórios 64

4.4 Descrição dos riscos (físicos, sociais e psicológicos) para o indivíduo ou grupo e métodos para minimizar estes riscos 65

4.4.1 Detalhamento dos riscos do estudo 65

4.4.2 Descrição dos benefícios antecipados ao participante do estudo 66

4.4.3 Descrição da relação entre os riscos potenciais e benefícios antecipados 66

4.4.4 Descrição de custos e justificativa de reembolsos ou compensações que serão usados 66

4.5 Confidencialidade 66

4.6 Declaração de conflito de interesses 67

4.7 Acesso aos dados 67

4.8 Seguros e cuidado de emergência 67

4.9 Comunicado aos participantes de seus resultados individuais 67

4.9.1 Divulgação dos resultados ao público 67

4.9.2 Comunicado aos participantes dos achados do estudo 68

4.9.3 Acesso de terceiros aos dados do estudo 68

5 REFERÊNCIAS 68

Índice de tabelas

Tabela 1 Estimativas de taxas de letalidade e de hospitalização por faixa etária entre casos infectados por SARS-CoV-2 na China [19] 17

Tabela 2. Incidência de reações adversas no estudo fase II da vacina adsorvida COVID-19 (inativada) [31] 20

Tabela 3. Taxas de soroconversão por anticorpos neutralizantes e ELISA IgG [31] 21

Tabela 4 Diagrama de procedimentos do estudo. 39

Tabela 5 Classificação da intensidade dos eventos adversos clínicos locais solicitados 53

Tabela 6 Classificação da intensidade dos eventos adversos clínicos sistêmicos solicitados e dos sinais e sintomas no caso de febre e suspeita de COVID-19. 53

Tabela 7 Classificação da intensidade dos Eventos Adversos clínicos não solicitados e de outros sinais e sintomas no caso de febre e suspeita de COVID-19 55

Tabela 8 Classificação da relação causal dos Eventos Adversos com o produto sob investigação 57

Tabela 9. Escala de progressão clínica de infecção por SARS- CoV-2. Adaptada de proposta da Organização Mundial da Saúde[41] 61

Índice de Figuras

Figura 1 Acompanhamento por 36 meses dos títulos de anticorpos de SARS-CoV-1 [4] 13

Figura 2 Representação esquemática do genoma (A) e filogenia (B) do SARS-CoV-2 [8] 14

Figura 3 Distribuição dos resultados de PCR e sorologia por intervalo de tempo entre o início de sintomas e a coleta de amostra em 496 casos de COVID-19 em um programa de vigilância comunitária [18] 16

1 INFORMAÇÃO ADMINISTRATIVA

1.1 TÍTULO DO PROTOCOLO

COV-02-IB – Ensaio Clínico Fase III duplo-cego, randomizado, controlado com placebo para Avaliação de Eficácia e Segurança em Profissionais da Saúde da Vacina adsorvida COVID-19 (inativada) produzida por Sinovac – PROFISCOV

1.2 INFORMAÇÃO DE REGISTRO

**Categoria**		**Informação**
Registro Primário		ClinicalTrials.gov NCT04456595
Data de registro		02-Jul-2020
Fontes de apoio financeiro ou material		Fundação Butantan<br>Instituto Butantan
Patrocinador primário		Instituto Butantan
Contato para questões públicas e cientificas		Ricardo Palacios, MD, PhD<br>Diretor Médico de Pesquisa Clínica<br>Instituto Butantan<br>Av. Vital Brasil, 1500<br>São Paulo, SP, Brasil CEP 05503-900<br>Telefone: (11)37232121<br>E-mail: ricardo.palacios@butantan.gov.br
Título científico		Ensaio Clínico Fase III duplo-cego, randomizado, controlado com placebo para Avaliação de Eficácia e Segurança em Profissionais da Saúde da Vacina adsorvida COVID-19 (inativada) produzida pela Sinovac
Título público		Ensaio Clínico Fase III para Avaliação de Eficácia e Segurança em Profissionais da Saúde da Vacina COVID-19 Inativada Produzida pela Sinovac
Código do estudo		COV-02-IB
Acrônimo do estudo		PROFISCOV
Países de recrutamento		Brasil
Condições ou problemas de saúde		COVID-19
Intervenções	Nome	Vacina (CoronaVac) Adsorvida COVID-19 (inativada)
	Descrição	Dose 600 SU/dose<br>Via: Intramuscular (deltoide)
	Nome	Placebo
Critérios de inclusão		a. Adultos com 18 anos de idade ou mais;<br>b. Profissionais de saúde realizam atendimento em contato direto com pessoas com quadros possíveis ou confirmados de COVID-19;<br>c. Concordar com contatos periódicos por telefone, meios eletrônicos e visitas domiciliares;<br>d. Demonstrar intenção de participar do estudo, documentada pela assinatura do Termo de consentimento livre e esclarecido por parte do participante.
Critérios de exclusão		e. Para o sexo feminino: Gravidez (confirmada por teste β-hCG positivo), estar amamentando e/ou manifestar intenção de ter práticas sexuais com potencial reprodutivo sem uso de método contraceptivo nos três meses seguintes à vacinação;

f. Evidência de doença ativa neurológica, cardíaca, pulmonar, hepática ou renal não controlada, conforme anamnese ou exame físico. Mudanças significativas de tratamento ou internações por piora do quadro nos últimos três meses são indicadores de doença não controlada;
g. Doenças com comprometimento do sistema imunológico inclusive: neoplasias (exceto carcinoma basocelular), imunodeficiências congênitas ou adquiridas e doenças autoimunes não controladas conforme anamnese ou exame físico. Mudanças significativas de tratamento ou internações por piora do quadro nos últimos três meses são indicadores de doença não controlada;
h. Doença comportamental, cognitiva ou psiquiátrica que, na opinião do investigador principal ou seu representante médico, afete a capacidade do participante em entender e colaborar com as exigências do protocolo do estudo;
i. Qualquer utilização considerada abusiva de álcool ou drogas nos últimos 12 meses anteriores à inclusão no estudo que tenha causado problemas médicos, profissionais ou familiares, como indicado pela história clínica;
j. História de reação alérgica grave ou anafilaxia à vacina ou componentes da vacina de estudo;
k. Histórico de asplenia;
l. Participação em outro ensaio clínico com administração de produto sob investigação durante os seis meses antecedentes a sua inclusão no estudo ou participação programada em outro ensaio clínico nos dois anos seguintes à inclusão;
m. Participação prévia em estudo de avaliação de vacina de COVID-19 ou exposição prévia a uma vacina de COVID-19;
n. Uso de terapias imunossupressoras seis meses anteriores à inclusão no estudo ou seu uso programado nos dois anos seguintes à inclusão. Serão consideradas terapias imunossupressoras: quimioterapia antineoplásica, radioterapia, imunossupressores para induzir tolerância a transplantes, entre outras.
o. Ter recebido dose imunossupressora de corticoide nos últimos três meses antes da sua inclusão no estudo ou administração programada de dose imunossupressora de corticoide para os três meses seguintes à inclusão no estudo. A dose de corticoide considerada imunossupressora é a equivalente à prednisona na dose de 20 mg/dia para adultos, por mais de uma semana. O uso contínuo de corticoide tópico ou nasal não é considerado imunossupressor;
p. Ter recebido hemoderivados (transfusões ou imunoglobulinas) nos últimos três meses antes da sua inclusão no estudo, ou administração programada de hemoderivados ou imunoglobulina nos dois anos seguintes à inclusão no estudo;
q. Suspeita ou confirmação de febre nas 72 horas que antecedem à vacinação ou temperatura axilar superior a 37,8°C* no dia da vacinação (a inclusão pode ser adiada até que o participante complete 72 horas sem febre);
r. Caso possível ou confirmado de COVID-19 no dia da vacinação (a vacinação pode ser adiada até que o participante complete 72 horas sem sintomas ou seja descartado diagnóstico);
s. Ter recebido vacina com vírus vivo atenuado nos últimos 28 dias ou vacina inativada nos últimos 14 dias que antecedem a sua inclusão no

	estudo, ou ainda ter imunização programada para os primeiros 28 dias após sua inclusão no estudo;<br>t. História de distúrbios hemorrágicos (por exemplo: deficiência de fatores de coagulação, coagulopatia, disfunção de plaquetas), ou história anterior de sangramento ou contusões significantes após injeção IM ou punção venosa;<br>u. Qualquer outra condição que, na opinião do investigador principal ou do seu representante médico, possa colocar em risco a segurança ou os direitos de um participante em potencial ou que o impeça de cumprir com este protocolo.<br>** A temperatura aferida com termômetro cutâneo de scanner temporal é considerada equivalente à temperatura axilar.*
Tipo de estudo	Ensaio clínico de Fase III, randomizado, multicêntrico, duplo cego e placebo controlado para avaliar a eficácia e segurança da Vacina adsorvida COVID-19 (inativada) produzida pela Sinovac.
Tipo de alocação	Estudo clínico randomizado controlado 1:1
Status de recrutamento:	Em andamento
Data do 1º recrutamento	21 de Julho de 2020
Tamanho da amostra alvo	13060 participantes
Desfecho primário	• Eficácia: O desfecho primário de eficácia é a densidade de incidência geral de casos sintomáticos de COVID-19 confirmados virologicamente após duas semanas da segunda vacinação. O diagnóstico virológico será confirmado por detecção de ácido nucleico do SARS-CoV-2 em amostra clínica.<br>• Segurança: O desfecho primário de segurança é a frequência de reações adversas locais e sistêmicas solicitadas e não solicitadas durante o período de uma semana após a vacinação de acordo com a faixa etária (Adultos de 18-59 anos e Idosos com 60 anos de idade ou mais). As reações adversas são definidas como eventos adversos que tenham relação causal razoável com a vacinação.
Desfechos secundários	Eficácia:<br>• A densidade de incidência de casos de COVID-19 confirmados virologicamente após duas semanas da segunda vacinação segundo exposição prévia a SARS-CoV-2. A exposição prévia a SARS-CoV-2 será comprovada por teste anterior ou o dia da primeira vacinação de detecção de ácido nucleico do SARS-CoV-2 em amostra clínica ou por teste positivo de anticorpos contra SARS-CoV-2 no dia da primeira vacinação.<br>• A densidade de incidência de casos de COVID-19 confirmados virologicamente após administração da vacina adsorvida COVID-19 (inativada) produzida pela Sinovac, duas semanas após a primeira vacinação.<br>• A densidade de incidência geral de infecções sintomáticas e assintomáticas por SARS-CoV-2 detectadas sorologicamente e/ou virologicamente, duas semanas após a segunda vacinação. A confirmação sorológica de infecções por SARS-CoV-2 será por um aumento de quatro vezes do nível dos títulos de IgG em ensaios sorológicos validados.

- A densidade de incidência de casos graves de COVID-19 confirmados virologicamente duas semanas após a administração da segunda dose da vacina adsorvida COVID-19 (inativada).

Segurança:

- A frequência de reações adversas à vacina, locais e sistêmicas, solicitadas e não solicitadas, depois de cada uma das duas doses do esquema de vacinação dentro do período de quatro semanas após a vacinação de acordo com o grupo etário (Adultos de 18-59 anos e idosos com 60 anos de idade ou mais).
- A frequência de casos de COVID-19 graves em participantes que receberam, ao menos, uma dose do produto experimental.
- A frequência de casos de Eventos Adverso de Interesse Especial em participantes que receberam, ao menos, uma dose do produto experimental.

Imunogenicidade:

- As taxas de soroconversão na segunda semana após cada vacinação de acordo com o grupo etário (Adultos de 18-59 anos e Idosos com 60 anos de idade ou mais).
- A resposta imune mediada por células em um subgrupo de participantes antes de cada vacinação e duas e quatro semanas após a segunda vacinação de acordo com o grupo etário (Adultos de 18-59 anos e Idosos com 60 anos de idade ou mais).
- A frequência de detecção de anticorpos contra SARS-CoV-2 antes e duas semanas após a administração da segunda dose da Vacina adsorvida COVID-19 (inativada) produzida pela Sinovac em Adultos (18-59 anos de idade) e Idosos (60 anos de idade ou mais). A avaliação será realizada em todos os participantes da pesquisa através de metodologia de ELISA e/ou Quimiluminescência validadas

1.3 Histórico de versões

Versão	Data	Comentário
1.0	30-Jun-2020	Versão inicial submetida à agência regulatória
1.1	03-Jul-2020	Versão incorporando considerações da agência regulatória
2.0	24-Ago-2020	Mudança no desenho do estudo e inclusão de participantes independente de exposição prévia

1.4 Financiamento

Este estudo tem financiamento da Fundação Butantan.

1.5 Responsáveis pelo protocolo

Os nomes e informações de contato dos responsáveis pelo protocolo estão detalhados no Anexo A.

1.5.1 Contribuições para o protocolo

Este protocolo de pesquisa foi escrito pela equipe do Instituto Butantan composta por Ricardo Palacios, Elizabeth González Patiño, Roberta de Oliveira Piorelli, Monica Tili Reis Pessoa Conde e Ana Paula Batista.

Os Drs Gang Zeng e Qianqian Xin, da Sinovac, e o Dr Esper Kallas, da Faculdade de Medicina da Universidade de São Paulo, também contribuíram para o protocolo. Os investigadores principais de cada centro também foram consultados sobre o conteúdo do protocolo. Na revisão do protocolo, em particular do desenho do estudo, participaram Jorge Flores, Christopher Gast e Christopher F. Ockenhouse de PATH nos Estados Unidos.

1.5.2 Papel do patrocinador e financiadores

As entidades financiadoras não terão influência no desenho e execução, na análise, na interpretação de dados nem na publicação dos resultados do estudo.

O Instituto Butantan, ligado à Secretária do Estado da Saúde de São Paulo, assume as responsabilidades de patrocinador do estudo em conformidade com as Boas Práticas Clínicas. O patrocinador fará a administração dos recursos financeiros para garantir o desempenho adequado do estudo e da plataforma para entrada de dados. O patrocinador também obterá as aprovações necessárias junto à Agência Nacional de Vigilância Sanitária. A Sinovac fornecerá o produto sob investigação.

As análises de dados serão realizadas por uma equipe do Instituto Butantan. Os pesquisadores terão acesso às bases de dados originais e poderão solicitar ou realizar análises adicionais conforme este protocolo, complementado por um plano de análise estatístico a ser finalizado antes do congelamento da base de dados. A publicação dos resultados do estudo será feita de comum acordo entre o patrocinador, a Sinovac e os investigadores, sem prejuízo do direito dos investigadores de apresentar o estudo e suas conclusões em revistas e encontros científicos.

1.5.3 Comitês do estudo

A equipe de estudo por parte do patrocinador será composta pelo representante do patrocinador, os especialistas médicos do patrocinador e a coordenação de monitoramento do estudo. Essa equipe estará encarregada de administrar o estudo e as responsabilidades relacionadas ao patrocinador. Os especialistas médicos do patrocinador farão a revisão médica dos eventos adversos pelo patrocinador.

Um Comitê Independente de Monitoramento de Dados e Segurança será constituído para o estudo conforme descrito na Seção 3.15.1.

2 Introdução

2.1 Revisão da literatura/Estado atual do conhecimento sobre o tema do projeto

2.1.1 Infecções por coronavírus em seres humanos

Os coronavírus são vírus RNA de genoma relativamente grande ≈ 30kB que se apresentam de forma ubíqua na natureza em diversas espécies animais, inclusive causando doenças em animais domésticos e de cria industrial. Antes do surgimento da COVID-19, foram identificados seis coronavírus capazes de causar infecção em seres humanos. Na década de 1960, dois coronavírus causadores de quadros de resfriado comum foram identificados, HCoV-OC43 e HCoV-229E. Já no presente século, foram acrescentados mais dois coronavírus como agentes associados a resfriado comum, HCoV-NL63 e HCoV-HKU1. Todos eles, exceto o último, têm descritas relações de proximidade genética com coronavírus de morcegos, que poderiam ter a ver com sua origem. Os dois coronavírus restantes, também identificados

no presente século, foram SARS-CoV-1, associado a surtos de Síndrome Respiratória Aguda Grave que começaram em 2002, e o MERS-CoV encontrado em 2012, associado também a quadros de doença grave em pessoas em contato com camelos e trabalhadores de saúde de locais que atendiam esses pacientes [1].

A resposta imune de seres humanos aos coronavírus parece ser heterogênea e transitória. Em um modelo de infecção controlada com HCoV-229E, a infecção foi bem-sucedida em 10 participantes com títulos de anticorpos neutralizantes tipo IgG e de IgA inferiores a outros 5 participantes que não desenvolveram a infecção dando à imunidade humoral para essa infecção. Após a infecção experimental, esses títulos aumentaram[2]. No caso de SARS-CoV-1, foi demostrada queda nos níveis tanto de IgA como de anticorpos neutralizantes de tipo IgG em seguimento de 36 meses (Figura 1), embora não se conheça a significância clínica, por não existir evidência de reexposição. Ainda, foi encontrado que uma das sequelas do quadro clínico, a necrose femoral, foi associada com títulos menores de anticorpos [3]. Essa queda pode ser ainda maior em pacientes com SARS-CoV-1 convalescentes 4 e 6 anos depois, com perda de resposta de anticorpos neutralizantes, que permanece presente em apenas 50% e 8,7%, dos indivíduos, respectivamente [4]. Em relação a MERS-CoV também foi descrita a queda de anticorpos neutralizantes ao longo do tempo em um número limitado de pacientes. Da mesma forma, também não se conhece o significado clínico e não foi possível documentar reexposição nesses casos [5]. Essas informações de heterogeneidade da resposta imune de coronavírus em seres humanos poderiam acontecer também em SARS-CoV-2 e teriam relevância para o potencial desenvolvimento de imunobiológicos.

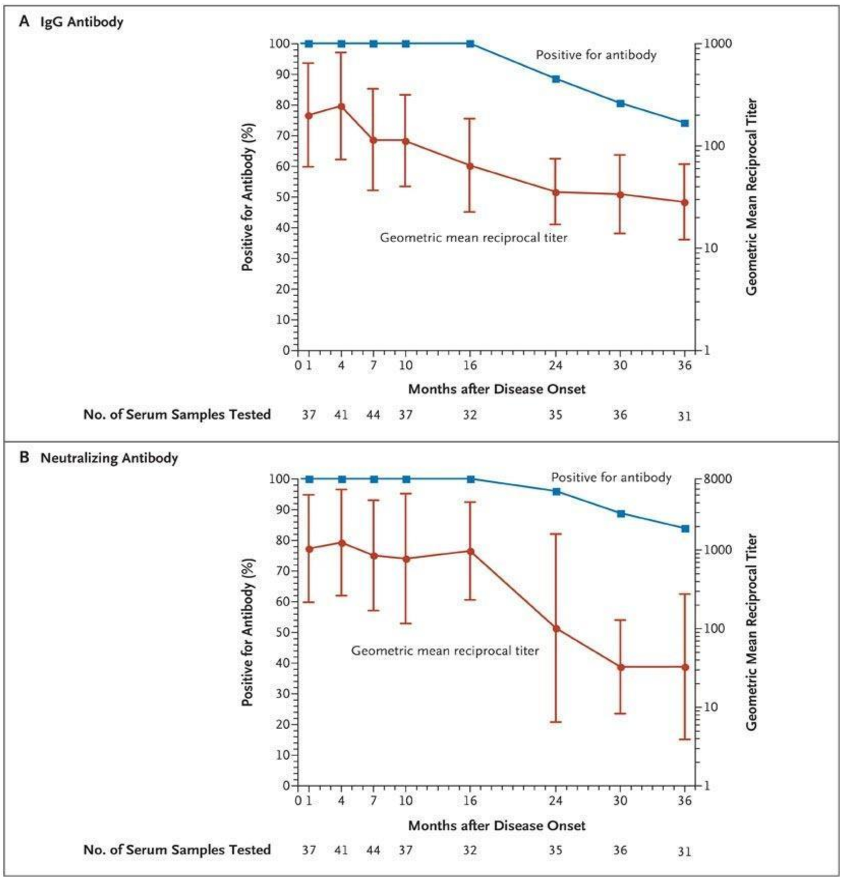

Figura 1 Acompanhamento por 36 meses dos títulos de anticorpos de SARS-CoV-1 [4]

2.1.2 A EMERGÊNCIA DO SARS-COV-2

No final do mês de dezembro de 2019, um agregado de pacientes com pneumonia foi identificado em associação com um mercado na cidade de Wuhan, na Província de Hubei, na China. De amostras coletadas de lavagem bronco-alveolar de três desses pacientes foi identificado um coronavírus diferente daqueles conhecidos infectando seres humanos. O novo coronavírus foi identificado como um betacoronavírus, assim como o SARS-CoV-1, com o qual possui mais proximidade genética (Figura 2) [6]. Esse vírus seria denominado posteriormente como SARS-CoV-2

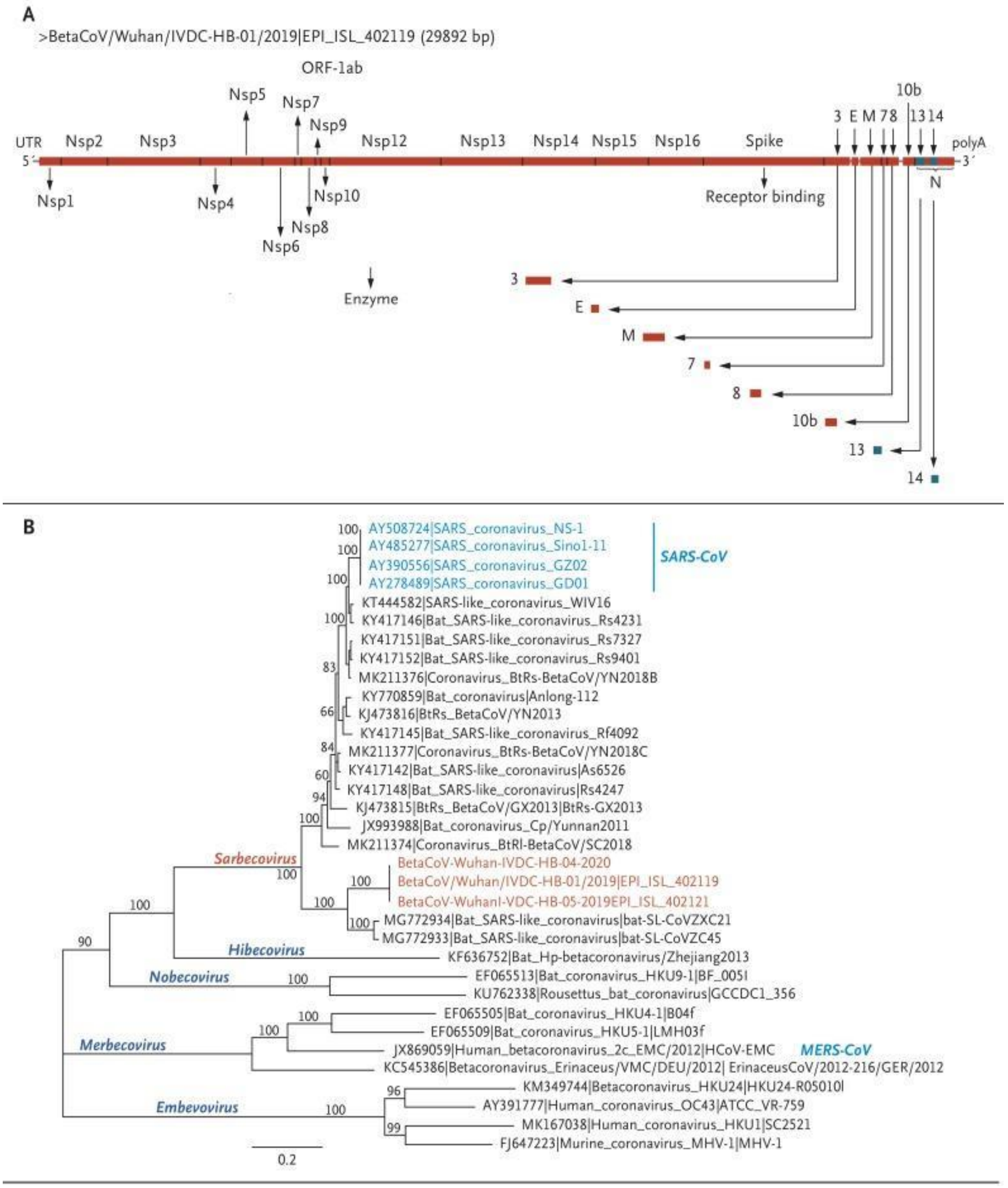

Figura 2 Representação esquemática do genoma (A) e filogenia (B) do SARS-CoV-2 [8]

O SARS-CoV-2 é um vírus esférico ou pleomórfico composto de um RNA de fita simples associado a uma nucleoproteína dentro de uma cápside proteica recoberta por um envelope com glicoproteínas

projetadas ao exterior em forma de espícula, ou Spike em inglês. Essa proteína Spike é fundamental para sua replicação, em forma análoga ao SARS-CoV-1, e os anticorpos neutralizantes estão direcionados principalmente contra ela [7].

A origem do SARS-CoV-2 é zoonótica com proximidade genética com betacoronavírus de morcegos, entre eles o RaTG13, encontrado no morcego-ferradura intermediário *(Rhinolophus affinis*), o mais próximo geneticamente. Mas a característica mais relevante desse SARS-CoV-2 é o Domínio de Ligação do Receptor 2 da Enzima Convertidora de Angiotensina (ACE2) na glicoproteína Spike que permite a entrada do vírus nas células de diversos tecidos humanos. Esse domínio é muito semelhante ao encontrado em coronavírus que infectam o pangolim-malaio (*Manis javanica*) [8]. As mutações até o momento parecem limitadas, embora alguns pesquisadores propõem que teria uma diferenciação em três tipos (A, B e C) com alteração pontual em aminoácidos [9].

A transmissão da infecção de SARS-CoV-2 é principalmente por gotículas e fômites como muitos outros vírus respiratórios, podendo se manter viável por várias horas e inclusive dias em diferentes superfícies [10]. A replicação em vias aéreas superiores facilita a transmissão pessoa a pessoa e a contaminação de áreas.

2.1.3 Apresentação clínica

Desde os relatos iniciais de casos da doença na cidade de Wuhan, o quadro mais grave do espectro clínico de COVID-19 foi bem caracterizado e constante. A apresentação é de um quadro febril com sintomas respiratórios associados tais como tosse, que evolui para pneumonia bilateral com comprometimento difuso que pode evoluir para síndrome de angustia respiratória aguda, lesão cardíaca e falência múltipla de órgãos. Essa evolução clínica grave foi mais frequente em pacientes de idade mais avançada e pessoas com condições médicas subjacentes como hipertensão, diabetes e doença cardiovascular, entre outras [11]. Essa associação com condições médicas subjacentes tem se reiterado em outras series de casos [12].

Os casos de COVID-19 se apresentam predominantemente com febre e que pode estar acompanhada de fadiga, tosse não produtiva ou com expectoração. Outros sintomas incluem mialgia, anorexia, sensação de aperto torácico e dispneia. Sintomas menos frequentes são náusea e vômito, diarreia, cefaleia, dor na faringe, calafrios e dor abdominal [13]. Cabe destacar entre os sintomas leves e moderados as alterações de olfato e paladar, que se apresentaram mais comumente entre mulheres [14]. Em crianças, os sintomas de quadros de COVID-19 parecem ser mais leves e menos frequentes, inclusive a febre e a tosse em comparação com adultos [15]. A evolução clínica em crianças também parece ser mais benigna com poucos pacientes evoluindo para formas graves [16]. A identificação rotineira de casos assintomáticos não é realizada atualmente, ainda que há alguns relatos principalmente associados à investigação epidemiológica de contatos de casos índice, demonstrando a ocorrência de transmissão de forma eficiente inclusive em pessoas jovens [17].

Num estudo recente de vigilância comunitária de COVID-19, a mediana de dias entre os que tiveram confirmação virológica por RT-PCR foi de 5 dias (P_{25-75} 4 -7 dias) sendo que houve um incremento no número de ciclos para detecção do vírus, que indica diminuição de carga viral na amostra, na medida que aumentava o tempo de sintomas [18].

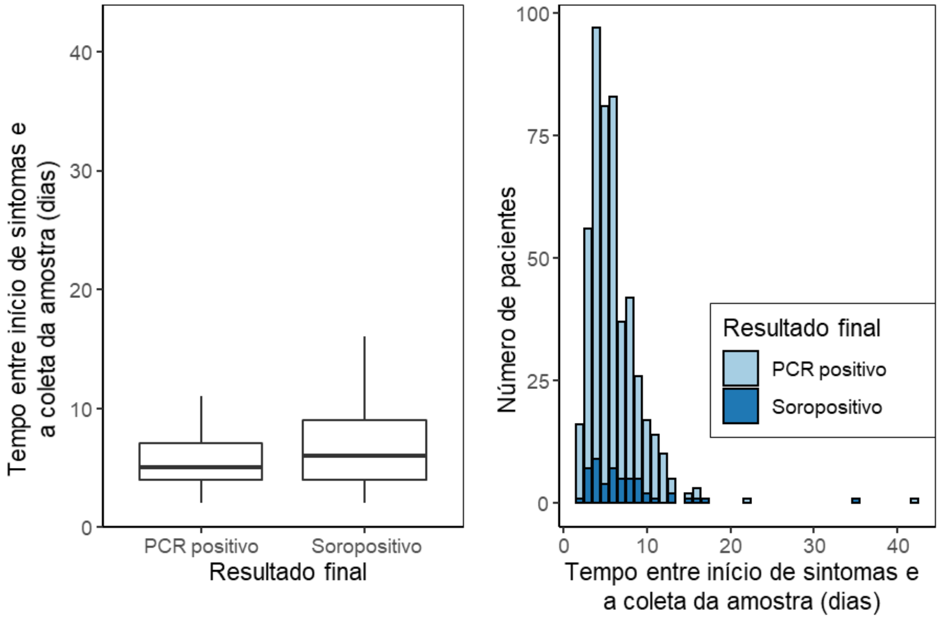

Figura 3 Distribuição dos resultados de PCR e sorologia por intervalo de tempo entre o início de sintomas e a coleta de amostra em 496 casos de COVID-19 em um programa de vigilância comunitária [18]

2.1.4 Epidemiologia da COVID-19

Em junho de 2020, o número de casos confirmados de COVID-19 no mundo ultrapassou os 10 milhões de pessoas diagnosticadas e com mais de meio milhão de óbitos. Esses dados são atualizados diariamente pela Organização Mundial de Saúde em site específico (https://covid19.who.int/) que permite ainda avaliar a expansão geográfica da pandemia, que atualmente atingiu quase a totalidade de países e territórios no mundo. A região das Américas é a mais atingida com perto da metade dos casos e dos óbitos relatados. Os Estados Unidos e o Brasil são os países mais atingidos na região e no mundo em números absolutos.

Considerando as limitações que a pandemia impõe ao sistema de saúde e a indisponibilidade de testes para diagnóstico de todos os casos suspeitos, foi necessária a priorização dos testes para os casos sintomáticos e entre eles, os de maior gravidade clínica. Por essa razão, o número real de casos de infeção por SARS-CoV-2 e de casos mais leves de COVID-19 ainda não está precisamente determinado. No entanto, alguns modelos matemáticos permitem fazer estimativas em relação a casos que precisam de hospitalização e que evoluem para óbito, conforme apresentado na Tabela 1 [19].

Em relação ao Brasil, o primeiro caso de COVID-19 foi detectado em 25 de fevereiro de 2020 e desde então há registro de casos em todas as unidades federativas sendo relatados até Junho perto 1.400.000 casos e quase sessenta mil óbitos, número esses que têm se incrementado diariamente.

Tabela 1 Estimativas de taxas de letalidade e de hospitalização por faixa etária entre casos infectados por SARS-CoV-2 na China [19]

Faixa etária	Taxa de letalidade entre casos confirmados de infecção por SARS-CoV-2	Taxa de letalidade entre casos estimados de infecção por SARS-CoV-2	Taxa de hospitalização entre casos estimados de infecção por SARS-CoV-2
**0-9 anos**	0·00260% (0·000312–0·0382)	0·00161% (0·000185–0·0249)	0·00% (0·00–0·00)
**10-19 anos**	0·0148% (0·00288–0·0759)	0·00695% (0·00149–0·0502)	0·0408% (0·0243–0·0832)
**20-29 anos**	0·0600% (0·0317–0·132)	0·0309% (0·0138–0·0923)	1·04% (0·622–2·13)
**30-39 anos**	0·146% (0·103–0·255)	0·0844% (0·0408–0·185)	3·43% (2·04–7·00)
**40-49 anos**	0·295% (0·221–0·422)	0·161% (0·0764–0·323)	4·25% (2·53–8·68)
**50-59 anos**	1·25% (1·03–1·55)	0·595% (0·344–1·28)	8·16% (4·86–16·7)
**60-69 anos**	3·99% (3·41–4·55)	1·93% (1·11–3·89)	11·8% (7·01–24·0)
**70-79 anos**	8·61% (7·48–9·99)	4·28% (2·45–8·44)	16·6% (9·87–33·8)
**80 anos ou mais**	13·4% (11·2–15·9)	7·80% (3·80–13·3)	18·4% (11·0–37·6)
**Total**	1·38% (1·23–1·53)	0·657% (0·389–1·33)	

2.1.5 Resposta imune contra o vírus SARS-CoV-2

A resposta imune a nível populacional contra o SARS-CoV-2 ainda não é muito bem determinada, embora resultados preliminares indicam que se assemelha a outros coronavírus em termos de heterogeneidade e duração. Um dos trabalhos que evidenciaram melhor essa variabilidade comparou 37 pacientes sintomáticos e 37 assintomáticos no período agudo e na convalescência 8 semanas depois [20]. Nesse estudo foi achado que a excreção viral foi mais prolongada entre os pacientes assintomáticos. De outro lado, a resposta de anticorpos, tanto IgG como IgM foi significativamente maior entre as pessoas sintomáticas. Em 93% dos pacientes assintomáticos e 97% dos pacientes sintomáticos houve queda importante dos títulos de IgG após 8 semanas, queda de 71% e 76%, respectivamente. Houve redução análoga em teste de anticorpos neutralizantes. Na amostra convalescente, 40% dos assintomáticos e 13% dos sintomáticos passaram a testar negativo para IgG apesar da infecção prévia confirmada virologicamente. Esses resultados demonstram quedas de IgG mais dramáticas que aquelas documentadas com SARS-CoV-1. Casos de infecção por SARS-CoV-2 mais leves parecem se restringir a vias respiratórias superiores, tendo menor oportunidade de estímulo de resposta imune sistêmica, em contraste com casos mais sintomáticos onde a infecção poderia comprometer mais órgãos e gerar uma resposta imune amplificada.

Nos casos graves, a queda de linfócitos parece ter um papel fundamental. A invasão do SARS-CoV-2 a diferentes órgãos que possuem receptores ACE2 parece não ser controlada pela resposta de anticorpos neutralizantes, possivelmente por queda específica dos níveis de linfócitos B. Não existe uma explicação para essa queda, mas IL-6 parece ter um papel importante na patogênese o que tem motivado o uso experimental de anticorpos monoclonais contra essa citocina para tratamento de formas graves de COVID-19. Também cabe anotar que os níveis de neutrófilos e dímero D são maiores em aqueles pacientes

que evoluem a óbito o que indica uma resposta inflamatória exagerada com coagulação intravascular, manifesta inclusive com quadros trombóticos e de vasculites. A recuperação da capacidade de produção de anticorpos poderia ser um dos fatores chaves para a recuperação nos casos graves de COVID-19 [21].

2.1.6 Desenvolvimento de vacinas contra a COVID-19

Desde a emergência da COVID-19, diversos esforços foram realizados para desenvolver uma vacina com velocidade nunca antes vista. Diferentes tecnologias têm sido utilizadas, sendo que em menos de 6 meses, diferentes candidatos vacinais já tinham chegado a fase clínica. Alguns deles, como é o caso da vacina desenvolvida por Sinovac, se beneficiaram de desenvolvimento anterior contra SARS-CoV-1 que tinha atingido a fase I clínica em 2004 [22], enquanto outros foram adaptações de desenvolvimentos contra MERS-CoV, como é o caso da vacina usando o vetor ChAdOx1 que teve sua fase I clínica em 2018 [23]. Em geral, as tecnologias mais usadas são vacinas inativadas, de vetores viral (adenovírus, sarampo e outros) que podem ser replicantes ou não, ácidos nucléicos (DNA e RNA), proteicas e de subunidade por recombinação ou síntese, e partículas semelhantes a vírus (VLP). Boa parte das vacinas fundamentam-se unicamente na proteína Spike ou regiões dessa proteína, em particular a RBD [24] . Um número muito limitado de candidatos vacinais tem outras proteínas virais representadas o que representa um potencial risco para aquelas que só geram resposta contra a proteína Spike ou suas regiões em caso de mutações ou que a resposta a essa proteína seja insuficiente. Daí parte do interesse em ter vacinas inativadas e outras opções que tem representação de diversas proteínas virais.

Um dos riscos teóricos das vacinas contra COVID-19 vem de achados histopatológicos de modelos animais de desafio após imunização com vacinas contra outros coronavírus, SARS-CoV-1 e MERS-CoV. Nesses casos, animais imunizados que foram expostos experimentalmente ao respectivo coronavírus fizeram uma reação inflamatória no parênquima pulmonar de predomínio eosinófilico. Esse achado fez levantar receios pela possibilidade de potencialização de doença respiratória associada a vacina em vacinas para coronavírus [25]. Até o momento, esse fenômeno não foi relatado nos candidatos vacinais para COVID-19. Embora, recomenda-se que antes de iniciar ensaios clínicos com seres humanos sejam feitos experimentos de desafio em animais de experimentação para verificar a ausência dessa resposta inflamatória pulmonar anômala na infecção experimental por SARS-CoV-2 após imunização [26]. Cabe destacar que o adjuvante hidróxido de alumínio é postulado como tendo um efeito protetor contra esse fenômeno, além de estimular uma potente resposta humoral de anticorpos neutralizantes [25,27–29].

2.1.7 Desenvolvimento da vacina adsorvida COVID-19 (inativada) produzida pela Sinovac (CoronaVac)

A Vacina adsorvida COVID-19 (inativada) é desenvolvida pela empresa Sinovac Life Science Co., Ltd. (doravante denominada Sinovac). A Vacina adsorvida COVID-19 (inativada) é derivada do novo coronavírus SARS-CoV-2 (cepa CZ02) e cultivada em célula renal de macaco verde africano (Célula Vero), seguida por cultura, colheita, inativação, concentração, purificação e adsorção de hidróxido de alumínio. Ela contém o novo coronavírus (SARS-CoV-2), hidróxido de alumínio, hidrogenofosfato dissódico, di-hidrogenofosfato de sódio e cloreto de sódio. A vacina é livre de conservantes. A embalagem final é um frasco para injetáveis ou seringa preenchida com um volume extraível de 0,5 mL. As dosagens baixa, média e alta são 300SU, 600SU e 1200SU, respectivamente. A Vacina adsorvida COVID-19 (inativada) é indicada para a imunização ativa contra a doença causada pelo novo coronavírus.

2.1.7.1 Resumo do Desenvolvimento das cepas vacinais

A cepa CZ02 é isolada a partir de um swab de garganta de pacientes infectados com SARS-CoV-2. Após ser isolada, a cepa é identificada por sequenciamento de nucleotídeos. Os lotes-semente de três camadas foram estabelecidos e testados seguindo as exigências de Fabricação e Controle de Qualidade para a Vacina adsorvida COVID-19 (inativada), os resultados obtidos até o momento estiveram conforme os parâmetros de qualidade. Os lotes-semente preparados podem atender as demandas do desenvolvimento da Vacina adsorvida COVID-19 (inativada).

2.1.7.2 Estudos não clínicos

Em relação aos estudos pré-clínicos, foram realizados vários ensaios sobre a segurança (toxicidade de dose única, teste de anafilaxia sistêmica ativa e teste de toxicidade de dose repetida), efeito de proteção e de imunogenicidade da vacina.

2.1.7.2.1 Estudos de segurança

Em relação à segurança, foram realizados teste de toxicidade de dose única em ratos, teste de anafilaxia sistêmica ativa em porquinhos-da-índia, teste de toxicidade de dose repetida em ratos e *Macaca fascicularis e* nenhuma reação clínica anormal foi observada nesses testes, o peso corporal em todos os grupos de animais mostrou uma tendência normal de aumento durante o período do estudo indicando que a vacina apresenta boa segurança em animais.

2.1.7.2.2 Estudos de imunogenicidade

Os testes de imunogenicidade em ratos e camundongos foram conduzidos para avaliar os níveis de anticorpo neutralizante e de IgG de diferentes vacinas SARS-CoV-2 (com e sem adjuvante) em diferentes pontos no tempo, utilizando diferentes esquemas de imunização após imunização intraperitoneal de camundongos e imunização intramuscular de ratos com diferentes doses de vacinas para poder determinar a formulação, dosagem e esquema de imunização da vacina. Os resultados demonstraram imunogenicidade favorável da vacina SARS-CoV-2 com adjuvante, e na dosagem de 300 SU/dose, 600 SU/dose e 1200 SU/dose, e o esquema com duas doses de imunização foi selecionado para o estudo clínico.

2.1.7.2.3 Estudos de desafio viral

Em relação ao estudo de desafio viral em macacos do gênero macaca, doze macacos foram randomizados em 4 grupos para receber 2 doses da vacina em dosagem média ou alta (Dia 0,14) ou 3 doses de adjuvante (Dia 0,7,14) ou o mesmo volume de soro fisiológico. O desafio viral iniciou a partir do Dia 22 e Dia 23 após a vacinação de grupos com dosagem média ou alta da vacina, respectivamente. Os resultados indicaram que a vacina SARS-CoV-2 inativada possui efeito protetor significativo. Além disso, não foi observada amplificação dependente de anticorpo (do inglês, *antibody-dependent enhancement* ou *ADE*) durante o estudo [30].

2.1.7.3 Ensaios clínicos

2.1.7.3.1 Estudos em adultos

Objetivo: Avaliar a segurança, tolerabilidade e imunogenicidade preliminar de diferentes dosagens de vacina administradas em diferentes esquemas de imunização em adultos para determinar a dosagem adequada e o esquema de imunização avaliações clínicas avançadas.

Desenho: Estudo clínico de Fase I/II randomizado, duplo cego e controlado por placebo, em 144 adultos saudáveis na Fase I e 600 adultos saudáveis na Fase II com 18-59 anos de idade designados a receber 2 doses de vacina com dosagem média (3µg equivalentes a 600 SU) e alta (6µg equivalentes a 1200 SU) ou placebo em um esquema de emergência (Dia 0,14) ou esquema de rotina (Dia 0,28) para avaliar a segurança e imunogenicidade.

Para a segurança na Fase I, as taxas de incidência de reações adversas solicitadas nos grupos de dosagem alta, dosagem média e placebo foram de 37,50%, 25,00% e 8,33%, respectivamente. Para a imunogenicidade, a taxa de soroconversão da Fase I nos grupos de dosagem alta, dosagem média e placebo foram de 50,00%, 45,83% e 0,00%, respectivamente. O Título Geométrico Meio – GMT nos grupos de dosagem alta, dosagem média e placebo foram de 7,7, 5,6 e 2,0, respectivamente. A taxa de soropositividade para o anticorpo IgG nos grupos de dosagem alta, dosagem média e placebo foram de 95,83%, 75,00% e 8,33%, respectivamente. Enquanto o IgM foi de 45,83%, 20,83% e 0,00%, respectivamente. Os participantes do grupo placebo falharam na resposta imune induzida.

Na Fase II, as taxas de incidência de reações adversas nos grupos de dosagem alta, dosagem média e placebo estão descritas na TABELA 2 respectivamente. As taxas de incidência de reações adversas foram maiores em dois grupos de vacina do que no grupo placebo, no entanto, não foi encontrada diferença estatisticamente significativa entre os grupos de dosagem média e dosagem alta. As reações adversas foram na sua maioria de Grau 1 e nenhuma reação adversa grave ocorreu [31].

Tabela 2. Incidência de reações adversas no estudo fase II da vacina adsorvida COVID-19 (inativada) [31]

	**Esquema de 0e 14 dias**			**Esquema de 0e 28 dias**		
**Reações adversas**	**Dose média (N=120)**	**Dose alta (N=120)**	**Placebo (N=60)**	**Dose média (N=120)**	**Dose alta (N=120)**	**Placebo (N=60)**
Intensidade	33.3	35.0	21.7	19.2	19.2	18.3
Grau 1	32.5	35.0	21.7	19.2	19.2	18.3
Grau 2	3.3	0.83	0.0	0.0	2.5	0.0
Reações adversas solicitadas	32.5	33.3	21.7	19.2	19.2	16.7
Reações adversas sistêmicas	15.0	12.5	15.0	11.7	10.0	10.0
Febre (temperatura axilar)	3.3	0.8	1.7	3.3	3.3	1.7
Reação alérgica aguda	0.0	0.8	0.0	0.8	0.0	0.0
Mucosa e pele anormal	0.0	0.0	0.0	0.0	0.0	0.0
Diarreia	5.0	0.8	1.7	0.8	2.5	1.7
Anorexia	0.8	0.0	0.0	0.0	0.8	0.0

Reações adversas	Esquema de 0e 14 dias			Esquema de 0e 28 dias		
	**Dose média (N=120)**	**Dose alta (N=120)**	**Placebo (N=60)**	**Dose média (N=120)**	**Dose alta (N=120)**	**Placebo (N=60)**
Vômito	0.8	0.0	0.0	0.0	0.0	0.0
Náusea	1.7	2.5	0.0	1.7	0.0	0.0
Dor muscular (fora do local de injeção)	2.5	1.7	1.7	1.7	3.3	5.0
Cefaleia	1.7	3.3	1.7	2.5	0.8	0.0
Tosse	0.0	1.7	0.0	0.8	0.0	0.0
Fadiga	3.3	5.8	10.0	8.3	2.5	3.3
Reações adversas locais	21.7	25.8	10.0	10.0	12.5	10.0
Dor	20.8	25.0	10.0	10.0	10.8	10.0
Induração	0.0	0.8	0.0	0.0	0.0	0.0
Inchaço	1.7	2.5	0.0	0.0	0.8	1.7
Vermelhidão	1.7	1.7	0.0	0.0	0.8	0.0
Eritema	0.0	0.0	0.0	0.0	0.0	0.0
Prurido	0.8	0.8	0.0	0.0	0.8	0.0
Reações adversas não solicitadas	2.5	2.5	0.0	0.0	0.8	1.7
Doenças neurológicas	0.8	0.8	0.0	--	--	--
- Sonolência	0.0	0.8	0.0	--	--	--
- Tontura	0.8	0.0	0.0	--	--	--
Doença sistêmica e do local de injeção	1.7	1.7	0.0	0.0	0.8	1.7
- Dor torácica	0.0	0.8	0.0	--	--	--
- Despigmentação do local de injeção	0.8	0.0	0.0	0.0	0.8	1.7
- Hipostesia no local de injeção	0.8	0.8	0.0	--	--	--
Doenças cardíacas	0.0	0.8	0.0	--	--	--
- Palpitação	0.0	0.8	0.0	--	--	--

Na Fase II, a taxa de soroconversão nos grupos de dosagem alta, dosagem média e placebo foram de 98,32%, 92,37% e 3,33%, respectivamente (TABELA 3). O GMT nos grupos de dosagem alta, dosagem média e placebo foram de 34,5, 27,6 e 2,3, respectivamente. O GMT os grupos de dosagem alta e média foram comparáveis [31].

Tabela 3. Taxas de soroconversão por anticorpos neutralizantes e ELISA IgG [31]

Marcador	Esquema de 0 e 14 dias			Esquema de 0 e 28 dias		
	**Dose média**	**Dose alta**	**Placebo**	**Dose média**	**Dose alta**	**Placebo**
Anticorpos neutralizantes						
14 dias pós-vacinação	92.4 (86.0, 96.5)	98.3 (94.1, 99.8)	3.3 (0.4, 11.5)	--	--	--
28 dias pós-vacinação	94.1 (88.2, 97.6)	99.2 (95.4, 100.0)	0.0 (0.0, 6.0)	97.4 (92.7, 99.5)	100.0 (96.9, 100.0)	0.0 (0.0, 6.1)
Anticorpos IgG						

Marcador	Esquema de 0 e 14 dias			Esquema de 0 e 28 dias		
	Dose média	Dose alta	Placebo	Dose média	Dose alta	Placebo
Antes da vacinação	2.6 (0.5, 7.4)	2.5 (0.5, 7.3)	1.8 (0.1, 9.6)	1.7 (0.2, 6.0)	0.9 (0.0, 4.7)	1.7 (0.0, 9.1)
14 dias pós-vacinação	96.5 (93.9, 99.8)	100.0 (96.9, 100.0)	0.0 (0.0, 6.4)	--	--	--
28 dias pós-vacinação	97.4 (92.5, 99.5)	100.0 (96.9, 100.0)	0.0 (0.0, 6.3)	99.2 (95.3, 100.0)	100.0 (96.9, 100.0)	6.8 (1.9, 16.5)

Conclusão: Em conclusão, todos os dados dos ensaios clínicos de Fase I/II indicaram segurança e imunogenicidade favoráveis com o esquema de duas doses da Vacina adsorvida COVID-19 (inativada). O GMT nos grupos de dosagem alta e média foram comparáveis. Com base nos resultados, não observamos uma imunidade celular explícita induzida pela Vacina adsorvida COVID-19 (inativada), e mais estudos devem ser realizados. Não foram observadas alterações significativas nos fatores inflamatórios o que indica um risco pequeno de imunopatologia induzida pela Vacina adsorvida COVID-19 (inativada).

2.1.7.3.2 Estudos em idosos

Objetivo: Avaliar a segurança e a imunogenicidade preliminar de diferentes dosagens da vacina em uma população saudável, com mais de 60 anos de idade.

Desenho: Fase I/II é um estudo clínico randomizado, duplo cego controlado por placebo com uma população saudável de 72 participantes na Fase I e com uma população saudável de 350 participantes na Fase II acima de 60 anos de idade que foram designados a receber 2 doses de dosagem diferente da vacina ou placebo em um esquema de rotina (Dia 0,28) para avaliar a segurança e imunogenicidade.

Resultados: Este estudo clínico está em andamento.

2.1.7.4 Resposta imune celular induzida pela vacina

Para o estudo clínico de Fase I em adultos saudáveis, a resposta imune celular induzida pela vacina foi avaliada utilizando o indicador de taxa positiva de resposta de célula T específica 14 dias após a vacinação (IFN-γ, IL-6, IL2 e TNF- α) na fase I.

Para 72 participantes da Fase I, não foi observada antes vacinação resposta positiva de célula T específica. Catorze (14) dias após a primeira vacinação, as taxas positivas de IFN-γ nos grupos de dosagem alta, dosagem média e placebo foram de 20,83%, 45,83% e 8,33%, respectivamente. Não houve diferenças estatisticamente significativas após a primeira dose nos níveis de IL-6 e IL2. Em relação à TNF- α houve um pequeno incremento após a imunização, incremento de duas vezes no grupo de alta dose. A totalidade dos dados não forneceu evidências de uma relação dose-resposta. Com base nesses resultados, não foi observada nenhuma imunidade celular explícita induzida pela Vacina adsorvida COVID-19 (inativada) e mais estudos devem ser realizados.

2.2 Objetivos

2.2.1 Objetivos primários

- Avaliar a eficácia de duas doses da Vacina adsorvida COVID-19 (inativada) produzida pela Sinovac em indivíduos sintomáticos com 18 anos de idade ou mais, com confirmação virológica de COVID-19, duas semanas após a segunda vacinação que trabalham como profissionais de saúde realizando atendimento em contato direto com pessoas com quadros possíveis ou confirmados de COVID-19.
- Descrever a ocorrência de reações adversas da droga associadas com a administração de cada dose da Vacina adsorvida COVID-19 (inativada) produzida pela Sinovac até uma semana após a vacinação em Adultos (18-59 anos de idade) e Idosos (60 anos de idade ou mais) que trabalham como profissionais de saúde realizando atendimento em contato direto com pessoas com quadros possíveis ou confirmados de COVID-19.

2.2.2 Objetivos secundários

Avaliação da eficácia

a. Avaliar a eficácia de duas doses da Vacina adsorvida COVID-19 (inativada) produzida pela Sinovac em indivíduos sintomáticos com 18 anos de idade ou mais, com confirmação virológica de COVID-19, duas semanas após a última vacinação que trabalham como profissionais de saúde realizando atendimento em contato direto com pessoas com quadros possíveis ou confirmados de COVID-19 segundo exposição prévia a SARS-CoV-2.

b. Avaliar a eficácia de pelo menos uma dose da Vacina adsorvida COVID-19 (inativada) produzida pela Sinovac em indivíduos sintomáticos com 18 anos de idade ou mais, com confirmação virológica de COVID-19, duas semanas após a primeira vacinação que trabalham como profissionais de saúde realizando atendimento em contato direto com pessoas com quadros possíveis ou confirmados de COVID-19.

c. Avaliar a eficácia de duas doses de uma Vacina adsorvida COVID-19 (inativada) produzida pela Sinovac em infecções assintomáticas e sintomáticas por SARS-CoV-2 detectadas sorologicamente e/ou virologicamente, duas semanas após a segunda vacinação que trabalham como profissionais de saúde realizando atendimento em contato direto com pessoas com quadros possíveis ou confirmados de COVID-19.

d. Avaliar a eficácia da Vacina adsorvida COVID-19 (inativada) produzida pela Sinovac em casos graves de COVID-19 confirmados virologicamente, duas semanas após receberem a segunda vacinação em indivíduos com 18 anos de idade ou mais que trabalham como profissionais de saúde realizando atendimento em contato direto com pessoas com quadros possíveis ou confirmados de COVID-19.

Avaliação de Segurança

e. Descrever o início de reações adversas à droga solicitadas e não solicitadas associadas com a administração de cada uma das duas doses Vacina adsorvida COVID-19 (inativada) produzida

pela Sinovac dentro das quatro semanas após a vacinação Adultos (18-59 anos de idade) eIdosos (60 anos de idade ou mais).

f. Descrever a ocorrência de casos graves de COVID-19 em participantes que tenham recebido, ao menos, uma dose da Vacina adsorvida COVID-19 (inativada) produzida pela Sinovac.

g. Descrever a ocorrência de Evento Adversos de Interesse Especial em participantes que tenham recebido, ao menos, uma dose da Vacina adsorvida COVID-19 (inativada) produzida pela Sinovac.

Avaliação da Imunogenicidade

h. Avaliar a resposta imune à vacinação em um subgrupo de participantes duas semanas após a administração de cada dose da Vacina adsorvida COVID-19 (inativada) produzida pela Sinovac em Adultos (18-59 anos de idade) e Idosos (60 anos de idade ou mais).

i. Avaliar a resposta imune à vacinação mediada por células em um subgrupo de participantes antes de cada vacinação e às duas e quatro semanas (e potencialmente em outros tempos) após a administração da segunda dose da Vacina adsorvida COVID-19 (inativada) produzida pela Sinovac em Adultos (18-59 anos de idade) e Idosos (60 anos de idade ou mais).

j. Avaliar a presença de anticorpos contra SARS-CoV-2 antes e duas semanas após a administração da segunda dose da Vacina adsorvida COVID-19 (inativada) produzida pela Sinovac em Adultos (18-59 anos de idade) e Idosos (60 anos de idade ou mais).

2.2.3 Objetivos exploratórios

- Avaliar a resposta imune em casos incidentes de COVID-19 ocorridos no estudo.

- Examinar o nível de anticorpos induzidos pela Vacina adsorvida COVID-19 (inativada) produzida pela Sinovac em testes de laboratório adicionais para procurar um possível correlato de proteção.

- Caracterizar a resposta imune até um ano do estudo em um subgrupo de participantes.

- Determinar interações em potencial entre a vacinação e outras condições médicas subjacentes, por exemplo, diabetes, obesidade, dislipidemia, hipertensão e doença cardiovascular.

- Descrever a resposta imune em participantes com infecção prévia por SARS-CoV-2 que receberam a Vacina adsorvida COVID-19 (inativada) produzida pela Sinovac.

- Avaliar a eficácia de duas doses de uma Vacina adsorvida COVID-19 (inativada) produzida pela Sinovac em infecções reincidentes assintomáticas e sintomáticas por SARS-CoV-2 detectadas sorologicamente e virologicamente, duas semanas após a segunda vacinação em pessoas com infecção prévia documentada.

- Avaliar a eficácia de duas doses de uma Vacina adsorvida COVID-19 (inativada) produzida pela Sinovac em casos de evento adverso grave por todas as causas e por COVID-19, duas semanas após a segunda vacinação.

- Avaliar a eficácia de duas doses de uma Vacina adsorvida COVID-19 (inativada) produzida pela Sinovac em casos de óbito por todas as causas e por COVID-19, duas semanas após a segunda vacinação.

2.3 Desenho geral do estudo

Este é um ensaio clínico de Fase III, randomizado, multicêntrico guiado por desfecho, duplo cego, controlado com placebo para avaliar a eficácia e segurança da Vacina adsorvida COVID-19 (inativada) produzida pela Sinovac.

Participantes saudáveis e/ou participantes com a doença clinicamente controlada, de ambos os gêneros, com 18 anos de idade ou mais, que trabalham como profissionais de saúde realizando atendimento em contato direto com pessoas com quadros possíveis ou confirmados de COVID-19 serão incluídos neste estudo. Não será permitida a participação no estudo de mulheres grávidas e aquelas que estão amamentando, bem como as com intenção de engravidar dentro dos três meses após a vacinação. Todos os participantes serão incluídos após assinatura do Termo de Consentimento Livre e Esclarecido e as avaliações do estudo baseadas nos critérios de inclusão e de exclusão.

A Vacina adsorvida COVID-19 (inativada) produzida pela Sinovac (produto sob investigação) será comparada ao placebo. Participantes voluntários serão randomizados para receber uma dose intramuscular do produto sob investigação ou o placebo, em uma proporção de 1:1, estratificado por faixa etária (18 a 59 anos e 60 anos ou mais) e serão monitorados por um ano por vigilância ativa de COVID-19. Serão estabelecidas duas bases de dados de acordo com os grupos etários: uma de adultos (18-59 anos) e uma de Idosos (60 anos de idade ou mais).

O critério para determinar que a eficácia vacinal é satisfatória será atingir uma proteção de, ao menos, 50% conforme critério proposto pela Organização Mundial da Saúde e a FDA. O sucesso nesse critério será definido por um monitoramento sequencial com ajuste de limite inferior do intervalo de confiança 95% acima de 30% para o desfecho primário de eficácia.

Com o objetivo de descrever uma resposta imune induzida e explorar uma imunidade humoral correlata em potencial relacionada à vacinação com a Vacina adsorvida COVID-19 (inativada) produzida pela Sinovac, será realizada uma comparação entre a resposta imune desenvolvida por participantes que desenvolveram a doença (casos) e um subgrupo de 10% dos participantes que não desenvolveram a doença (controles). Se necessário, o subgrupo poderá ser aumentado para melhor embasar quaisquer conclusões. Todos os participantes terão amostras coletas antes, e duas semanas após e quatro semanas após cada dose da vacina para permitir a seleção descrita acima. A resposta imune será avaliada por títulos de anticorpos neutralizantes e testes ELISA para proteínas do SARS-CoV-2. Esses testes serão repetidos nas amostras das semanas 13, 26, 39 e 52.

Adicionalmente, um subgrupo menor de participantes, os 60 primeiros por faixa etária no primeiro centro de pesquisa, serão avaliados para marcadores de imunidade celular antes de cada vacinação e duas e quatro semanas após a segunda vacinação. As células coletadas serão estimuladas por *"megapools"* de peptídeos do SARS-CoV-2 reconhecíveis pelos HLA classe I e II [32] para detectar sua resposta de citocinas. Esses testes poderão ser repetidos nas amostras das semanas 13, 26, 39 e 52.

3 MÉTODOS

3.1 CENTROS DE ESTUDO

O estudo será realizado em centros de pesquisa clínica acessíveis para profissionais de saúde que trabalhem em áreas especializadas para o atendimento de pacientes com COVID-19. Os centros de estudo atuarão de uma forma integrada permitindo o acompanhamento dos participantes e a obtenção dos dados conforme descritos neste protocolo. Os dados de contato dos centros de estudo encontram-se no Anexo A.

3.2 PARTICIPANTES

Participantes sadios e/ou com doença clinicamente controlada, de ambos os sexos e com idade de 18 anos ou mais que trabalham como profissionais de saúde em unidades especializadas no tratamento de COVID-19 poderão ser incluídos no estudo. Os critérios de ingresso (inclusão e exclusão) no estudo deverão ser verificados antes da administração de cada uma das duas doses do esquema de imunização. Os participantes deverão cumprir todos os critérios de inclusão e não se enquadrarem em nenhum dos critérios de exclusão, descritos a seguir:

3.2.1 CRITÉRIOS DE INCLUSÃO

a. Adultos 18 anos de idade ou mais;

b. Profissionais de saúde que realizam atendimento em contato direto com pessoas com quadros possíveis ou confirmados de COVID-19;

c. Concordar com contatos periódicos por telefone, meios eletrônicos e visitas domiciliares;

d. Demonstrar intenção de participar do estudo, documentada pela assinatura do Termo de consentimento livre e esclarecido por parte do participante.

3.2.2 CRITÉRIOS DE EXCLUSÃO

a. Para o sexo feminino: Gravidez (confirmada por teste β-hCG positivo), estar amamentando e/ou manifestar intenção de ter práticas sexuais com potencial reprodutivo sem uso de método contraceptivo nos três meses seguintes à vacinação;

b. Evidência de doença ativa neurológica, cardíaca, pulmonar, hepática ou renal não controlada, conforme anamnese ou exame físico. Mudanças significativas de tratamento ou internações por piora do quadro nos últimos três meses são indicadores de doença não controlada;

c. Doenças com comprometimento do sistema imunológico inclusive: neoplasias (exceto carcinoma basocelular), imunodeficiências congênitas ou adquiridas e doenças autoimunes não controladas conforme anamnese ou exame físico. Mudanças significativas de tratamento ou internações por piora do quadro nos últimos três meses são indicadores de doença não controlada;

d. Doença comportamental, cognitiva ou psiquiátrica que, na opinião do investigador principal ou seu representante médico, afete a capacidade do participante em entender e colaborar com as exigências do protocolo do estudo;

e. Qualquer utilização considerada abusiva de álcool ou drogas nos últimos 12 meses anteriores à inclusão no estudo que tenha causado problemas médicos, profissionais ou familiares, como indicado pela história clínica;

f. História de reação alérgica grave ou anafilaxia à vacina ou componentes da vacina de estudo;

g. Histórico de asplenia;

h. Participação em outro ensaio clínico com administração de produto sob investigação durante os seis meses antecedentes a sua inclusão no estudo ou participação programada em outro ensaio clínico nos dois anos seguintes à inclusão;

i. Participação prévia em estudo de avaliação de vacina de COVID-19 ou exposição prévia a uma vacina de COVID-19;

j. Uso de terapias imunossupressoras seis meses anteriores à inclusão no estudo ou seu uso programado nos dois anos seguintes à inclusão. Serão consideradas terapias imunossupressoras: quimioterapia antineoplásica, radioterapia, imunossupressores para induzir tolerância a transplantes, entre outras.

k. Ter recebido dose imunossupressora de corticoide nos últimos três meses antes da sua inclusão no estudo ou administração programada de dose imunossupressora de corticoide para os três meses seguintes à inclusão no estudo. A dose de corticoide considerada imunossupressora é a equivalente à prednisona na dose de 20 mg/dia para adultos, por mais de uma semana. O uso contínuo de corticoide tópico ou nasal não é considerado imunossupressor;

l. Ter recebido hemoderivados (transfusões ou imunoglobulinas) nos últimos três meses antes da sua inclusão no estudo, ou administração programada de hemoderivados ou imunoglobulina nos dois anos seguintes à inclusão no estudo;

m. Suspeita ou confirmação de febre nas 72 horas que antecedem à vacinação ou temperatura axilar superior a 37,8°C* no dia da vacinação (a inclusão pode ser adiada até que o participante complete 72 horas sem febre);

n. Caso possível ou confirmado de COVID-19 no dia da vacinação (a vacinação pode ser adiada até que o participante complete 72 horas sem sintomas ou seja descartado o diagnóstico);

o. Ter recebido vacina com vírus vivo atenuado nos últimos 28 dias ou vacina inativada nos últimos 14 dias que antecedem a sua inclusão no estudo, ou ainda ter imunização programada para os primeiros 28 dias após sua inclusão no estudo;

p. História de distúrbios hemorrágicos (por exemplo: deficiência de fatores de coagulação, coagulopatia, disfunção de plaquetas), ou história anterior de sangramento ou contusões significantes após injeção IM ou punção venosa;

q. Qualquer outra condição que, na opinião do investigador principal ou do seu representante médico, possa colocar em risco a segurança ou os direitos de um participante em potencial ou que o impeça de cumprir com este protocolo.

* *A temperatura aferida com termômetro cutâneo de scanner temporal é considerada equivalente à temperatura axilar.*

3.3 Desfechos

3.3.1 Desfechos primários

a. O desfecho primário de eficácia é a densidade de incidência geral de casos sintomáticos de COVID-19 confirmados virologicamente após duas semanas da segunda vacinação. O diagnóstico virológico será confirmado por detecção de ácido nucleico do SARS-CoV-2 em amostra clínica.

b. O desfecho primário de segurança é a frequência de reações adversas locais e sistêmicas solicitadas e não solicitadas durante o período de uma semana após a vacinação de acordo com a faixa etária (Adultos de 18-59 anos e Idosos com 60 anos de idade ou mais). As reações adversas são definidas como eventos adversos que tenham relação causal razoável com a vacinação.

3.3.2 Desfechos secundários

Os desfechos secundários de eficácia são:

- A densidade de incidência de casos de COVID-19 confirmados virologicamente após duas semanas da segunda vacinação segundo exposição prévia a SARS-CoV-2. A exposição prévia a SARS-CoV-2 será comprovada por teste anterior ou o dia da primeira vacinação de detecção de ácido nucleico do SARS-CoV-2 em amostra clínica ou por teste positivo de anticorpos contra SARS-CoV-2 no dia da primeira vacinação.

- A densidade de incidência de casos de COVID-19 confirmados virologicamente após administração da vacina adsorvida COVID-19 (inativada) produzida pela Sinovac, duas semanas após a primeira vacinação.

- A densidade de incidência geral de infecções sintomáticas e assintomáticas por SARS-CoV-2 detectadas sorologicamente e/ou virologicamente, duas semanas após a segunda vacinação. A confirmação sorológica de infecções por SARS-CoV-2 será por um aumento de quatro vezes do nível dos títulos de IgG em ensaios sorológicos validados.

- A densidade de incidência de casos graves de COVID-19 confirmados virologicamente duas semanas após a administração da segunda dose da vacina adsorvida COVID-19 (inativada).

Os desfechos secundários de segurança são:

- A frequência de reações adversas à vacina, locais e sistêmicas, solicitadas e não solicitadas, depois de cada uma das duas doses do esquema de vacinação dentro do período de quatro semanas após a vacinação, de acordo com o grupo etário (Adultos de 18-59 anos e idosos com 60 anos de idade ou mais).

- A frequência de casos de COVID-19 graves em participantes que receberam, ao menos, uma dose do produto experimental.

- A frequência de casos de Eventos Adverso de Interesse Especial em participantes que receberam, ao menos, uma dose do produto experimental.

Os desfechos secundários de imunogenicidade são:

- As taxas de soroconversão na segunda semana após cada vacinação de acordo com o grupo etário (Adultos de 18-59 anos e Idosos com 60 anos de idade ou mais).

- A resposta imune mediada por células em um subgrupo de participantes antes de cada vacinação e duas e quatro semanas após a segunda vacinação de acordo com o grupo etário (Adultos de 18-59 anos e Idosos com 60 anos de idade ou mais).

- A frequência de detecção de anticorpos contra SARS-CoV-2 antes e duas semanas após a administração da segunda dose da Vacina adsorvida COVID-19 (inativada) produzida pela Sinovac em Adultos (18-59 anos de idade) e Idosos (60 anos de idade ou mais). A avaliação será realizada em todos os participantes da pesquisa através de metodologia de ELISA e/ou Quimiluminescência validadas.

3.3.3 Desfechos Exploratórios

- Anticorpos nos casos incidentes de COVID-19.

- Avaliação de resposta imune contra SARS-CoV-2 com testes laboratoriais adicionais.

- A avaliação da resposta imune em até um ano em um subgrupo de participantes.

- A densidade de incidência de casos de COVID-19 confirmados virologicamente, duas semanas após a vacinação, em participantes com condições médicas subjacentes, por exemplo, diabetes, obesidade, dislipidemia, hipertensão e doença cardiovascular.

- A avaliação de resposta imune em participantes com infecção prévia por SARS-CoV-2 que receberam a Vacina adsorvida COVID-19 (inativada) produzida pela Sinovac.

- A densidade de incidência geral de infecções reincidentes por SARS-CoV-2 detectadas sorologicamente e/ou virologicamente, duas semanas após a segunda vacinação em pessoas com infecção prévia documentada por diagnóstico virológico ou sorológico. A confirmação sorológica de infecções por SARS-CoV-2 será por um aumento de quatro vezes do nível dos títulos de IgG em ensaios sorológicos validados.

- A densidade de incidência de casos de eventos adversos graves por todas as causas e por COVID-19 confirmados virologicamente duas semanas após a administração da segunda dose da vacina adsorvida COVID-19 (inativada).

- A densidade de incidência de casos de óbito por todas as causas e por COVID-19 confirmados virologicamente duas semanas após a administração da segunda dose da vacina adsorvida COVID-19 (inativada).

3.4 Intervenções

3.4.1 Composição final e formulações da vacina adsorvida COVID-19 (inativada) produzida pela Sinovac

A vacina desenvolvida pela Sinovac Research & Development Co., Ltd. é um preparado fabricado a partir do novo coronavírus (cepa CZ02) e cultivada em célula renal de macaco verde africano (Célula Vero). Em seguida ao cultivo e colheita, a suspensão viral é inativada, concentrada, purificada e adsorvida por hidróxido de alumínio. Ela contém o coronavírus SARS-CoV-2, hidróxido de alumínio, hidrogenofosfato dissódico, di-hidrogenofosfato de sódio, cloreto de sódio. Seu nome comercial é: CoronaVac 0,5mL/dose contendo 600SU de antígeno do vírus SARS-CoV-2. A vacina é injetada por via intramuscular, e o esquema de vacinação são duas doses, com um intervalo de duas semanas entre elas.

O vírus SARS-CoV-2 é inativado duas vezes para garantir a biossegurança. A inativação é efetuada com a adição de β-propiolactona no líquido de colheita do vírus na proporção de 1:4000 (V/V) durante 12-24 horas de inativação a 2-8°C. O líquido coletado da inativação é então descartado com a β-propiolactona na proporção de 1:4000 e solução de formaldeído na proporção de 1:2000 para posterior tratamento a 2-8°C durante 2-8 horas, obtendo-se o líquido com SARS-CoV-2 inativado.

O parâmetro do processo de inativação definido de inativar 9 lotes de líquido de inativação e realizar a verificação da inativação deve ser seguido. Os resultados demonstram que seguindo o processo de inativação definido, o vírus SARS-CoV-2 pode ser completamente inativado o que demonstra que o processo de inativação é estável.

3.4.1.1 VACINA ADSORVIDA COVID-19 (INATIVADA) PRODUZIDA PELA SINOVAC

A vacina contém o vírus SARS-CoV-2 inativado, hidróxido de alumínio, hidrogenofosfato dissódico, di-hidrogenofosfato de sódio e cloreto de sódio.

3.4.1.1.1 Forma Farmacêutica e Embalagem

O produto final será fornecido em uma seringa pré-preenchida contendo 0,5 mL de solução injetável que corresponde a uma dose da vacina.

3.4.1.1.2 Dose/Administração/Armazenamento

A temperatura de armazenamento da vacina, como indicada no **rótulo do frasco** é de 2 a 8°C. A dose da vacina corresponde a 0,5 mL. O local de administração recomendado é o músculo deltoide do braço por injeção intramuscular. O esquema de imunização é de duas doses com um intervalo de duas semanas.

3.4.1.1.3 Lotes da vacina

Neste estudo, será utilizado um único lote de produção de vacina.

3.4.1.2 Indicações de uso/Contraindicações/Advertências/Precauções

Até que licenciada para uma indicação específica, a Vacina adsorvida COVID-19 (inativada) produzida pela Sinovac é para uso em investigação e deve ser administrada somente em sujeitos participantes de um estudo clínico conduzido de acordo com as Regulamentações Federais aplicáveis e com os critérios de inclusão e exclusão especificados em um Protocolo de Pesquisa previamente aprovado pelos órgãos éticos e regulatórios responsáveis.

Como muitos dos efeitos relacionados com a administração desta vacina experimental são desconhecidos pode existir um risco aumentado da ocorrência de eventos adversos se os sujeitos receberem outras drogas experimentais ou sob investigação. Por este motivo, é fundamental que os sujeitos que recebam os vírus vacinais não participem de quaisquer outros estudos com produtos sob investigação durante a condução deste estudo.

3.4.1.3 Composição final e formulação do placebo

3.4.1.3.1 Composição

O placebo contém hidróxido de alumínio, hidrogenofosfato dissódico, di-hidrogenofosfato de sódio, cloreto de sódio 0,5mL/dose, injeção intramuscular, duas doses administradas com duas semanas de intervalo.

3.4.1.3.2 Forma Farmacêutica e Embalagem

O produto final será fornecido em uma seringa pré-preenchida contendo 0,5 mL de solução injetável que corresponde a uma dose do placebo.

3.4.1.3.3 Dose/Administração/Armazenamento

A temperatura de armazenamento do placebo, como indicado no **rótulo do frasco** é de 2 a 8°C. A dose da vacina corresponde a 0,5 mL. O local de administração recomendado é o músculo deltoide do braço por injeção intramuscular. O esquema de imunização com o placebo é de duas doses com um intervalo de duas semanas.

3.4.1.4 Rótulo do produto sob investigação

Os rótulos do produto sob investigação serão elaborados conforme a regulação vigente. Amostras desses rótulos se encontram no Anexo B.

3.4.2 INTERVENÇÕES OU REGIMES DE TRATAMENTOS CONCOMITANTES PERMITIDOS, NÃO RECOMENDADOS E PROIBIDOS

Não há medicamentos proibidos neste protocolo. Entretanto, o uso dos seguintes produtos não é recomendado nas quatro semanas (28 dias) seguintes à administração do produto sob investigação:

- Qualquer droga ou vacina em investigação diferente da utilizada neste estudo;

- A administração crônica (≥14 dias) de corticoide sistêmico (definida como a administração equivalente a 10 mg ou mais de prednisona por dia), imunossupressores ou outras drogas imunomoduladoras;
- Qualquer vacina licenciada;
- Imunoglobulinas e/ou qualquer produto hemoderivado.

Se o uso de algum desses produtos nas primeiras quatro semanas (28 dias) após a vacinação for imprescindível este deverá ocorrer após orientação médica e o evento deverá ser anotado no registro individual do participante de pesquisa.

Se esse uso ocorreu após a primeira dose e antes da segunda dose, a segunda dose não será administrada.

Caso a equipe do centro de pesquisa identifique a necessidade de imunização adicional do participante, de acordo com as recomendações do Ministério de Saúde, este poderá ser encaminhado para ser vacinado após o prazo de quatro semanas (28 dias) após a vacinação prevista neste protocolo.

O uso de outras vacinas, mesmo que experimentais, direcionadas à prevenção de COVID-19 não é recomendado durante o período de seguimento do estudo. Caso aconteça, deverá ser anotado no registro individual do participante.

3.4.3 Risco de gravidez para participantes do sexo feminino

Todas as participantes do sexo feminino serão consideradas com potencial de engravidar, exceto aquelas que ainda não tiveram a primeira menstruação, tenham antecedente documentado de histerectomia, laqueadura ou estejam na menopausa (12 meses de amenorreia após seu último período menstrual).

Para participantes do sexo feminino com potencial de engravidar, o médico do estudo avaliará a necessidade de iniciar a utilização de um método contraceptivo antes da sua inclusão no estudo e da manutenção do seu uso até a semana 4 (dia 28) após ter recebido a última vacinação. Os métodos contraceptivos não serão obrigatórios caso a voluntária, ou sua representante legal, a declare isenta ao risco de engravidar, quer por não exercer práticas sexuais ou por exercê-las de forma não reprodutiva, até semana 4 após a última vacinação.

Essa discussão deverá abranger a efetividade dos diferentes métodos contraceptivos conforme a seguinte classificação proposta pelos Critérios de Elegibilidade Médica de Uso de Contraceptivos dos Centros de Controle de Doenças e Prevenção dos Estados Unidos [33]:

- Métodos mais efetivos (menos de um episódio de gravidez por cem mulheres-ano):
    - Implante;
    - Dispositivo intrauterino de cobre
    - Sistema intrauterino de levonorgestrel;
    - Vasectomia;
    - Esterilização feminina;
- Métodos efetivos (6 a 12 episódios de gravidez por cem mulheres-ano):
    - Injetáveis mensais;
    - Pílulas combinadas ou de progestagênios;
    - Anel vaginal;

  - Adesivo;
  - Diafragma com espermicida
- Métodos pouco efetivos (18 ou mais episódios de gravidez por cem mulheres-ano):
  - Preservativo masculino;
  - Preservativo feminino;
  - Coito interrompido;
  - Esponja;
  - Abstinência períodos férteis
  - Espermicida isolado.

Este estudo poderá encaminhar para planejamento familiar as voluntárias que o desejarem.

3.4.4 Procedimentos para verificar a adesão ao tratamento

O tratamento deste protocolo de pesquisa é a administração do produto sob investigação que será realizado nos centros de pesquisa por um membro da equipe do estudo.

3.5 Procedimentos para o participante

3.5.1 Acompanhamento dos participantes

Ao todo, cada participante será acompanhado de forma cega por um período de um ano após a sua inclusão no estudo com os objetivos de avaliar a ocorrência de casos de COVID-19 e sua resposta imune ao longo deste período.

3.5.2 Procedimentos para a realização do estudo

Após a divulgação do estudo, os participantes em potencial que se apresentarem serão recebidos pela equipe local de pesquisadores e serão indagados sobre a sua faixa etária, local de residência e doenças de base. A participação em estudos observacionais (ex. coortes), não será impeditiva para se tornar voluntário deste estudo pois não existem intervenções em tais estudos que possam interferir com o presente ensaio clínico.

Se não houver nenhum impedimento inicial para a participação no estudo, os pesquisadores fornecerão informações gerais sobre o estudo e aplicarão o Termo de Consentimento Livre e Esclarecido (TCLE) conforme legislação vigente. O processo de consentimento livre e esclarecido está descrito na Seção 4.3.1. Caso exista uma condição temporária que constitua um critério de exclusão, o potencial participante poderá ser convocado novamente para a visita de inclusão quando essa condição não existir.

Um participante será considerado como incluído no estudo no momento em que for vacinado (Dia 0).

3.5.3 Descrição dos procedimentos do estudo por visita

3.5.3.1 Visitas de vacinação (V1 e V2)

Nas visitas de vacinação (V1 e V2), serão verificados os critérios de inclusão e exclusão e sua elegibilidade através de história clínica e exame físico na V1 e serão revisados na V2. Uma vez considerado elegível, o participante será vacinado nesta mesma visita e este será considerado o Dia 0. A V1 pode ser dividida sendo possível fazer apresentação e assinatura do TCLE, assim como a obtenção de alguns dados

demográficos, antropométricos e de histórico médico até três dias antes da vacinação. A visita de vacinação 2 (V2) está programada para ocorrer duas semanas (+2 semanas de janela) após a visita de vacinação 1 (V1). A visita de vacinação é obrigatória para todos os voluntários.

Procedimentos:

a. Apresentar o estudo ao participante em potencial, realizar o procedimento de consentimento informado e documentar a decisão de participar no TCLE (somente na V1, pode ser realizada até três dias antes da vacinação);

b. Obter dados demográficos, peso, altura, circunferência abdominal e determinar condições médicas pré-existentes e uso de medicamentos concomitantes (Somente na V1, pode ser realizada até três dias antes da vacinação);

c. Aferir sinais vitais, e realizar a anamnese e o exame físico;

d. Verificar critérios de inclusão e exclusão clínicos;

e. Coletar amostras de sangue (amostras adicionais serão necessárias para os primeiros 60 participantes de cada faixa etária no primeiro centro de pesquisa);

f. Coletar amostra clínica para o teste laboratorial de detecção do vírus SARS-CoV-2 (somente na V1);

g. Realizar a vacinação (ver seção 3.5.4);

h. Fornecer ao participante o “Diário do Participante” e um (1) termômetro;

i. Orientar o participante quanto à técnica para a aferição da sua temperatura corporal e aferir a temperatura;

j. Observar o participante durante 60 minutos após a vacinação [34] e realizar avaliação dos eventos adversos solicitados;

k. Orientar sobre procura do centro de pesquisa em caso de febre ou outros sintomas de COVID-19;

As participantes com potencial de engravidar deverão passar pelos seguintes procedimentos antes da vacinação:

l. Exame de urina para detecção de β-hCG;

m. Revisão do risco de gravidez (ver seção 3.4.3);

3.5.3.2 Visitas de Resposta Imune e de Segurança (SI1 e SI2)

A intenção das visitas de segurança e resposta imune (SI1 e SI2) é de avaliar a ocorrência de reações adversas e coletar amostras para avaliar a resposta imune. Essas vistas deverão ser programadas na Semana dois e quatro após a segunda vacinação, com uma janela de até uma semana após a realização da visita, SI1: V2 + 2 semanas (+ 1 semana) e SI2: V2 + 4 semanas (+ 1 semana).

Procedimentos:

- Atualização de informações de contato, se necessário;
- Aferir sinais vitais e realizar anamnese, com atenção especial para qualquer queixa clínica, e exame físico para procurar eventos adversos solicitados e não solicitados;
- Revisão e coleta do "Diário do Participante" (ver seção 3.5.5);
- Coletar amostra de sangue (amostras adicionais serão necessárias para os primeiros 60 participantes de cada faixa etária no primeiro centro de pesquisa);
- Reforçar a importância de contatar a equipe do estudo em caso de febre ou outros sintomas de COVID-19;

Para as participantes com potencial de engravidar:

- Exame de urina para detecção de β-hCG (só na SI1);

3.5.3.3 Visitas de Resposta Imune (I1, I2, I3 e I4)

O propósito destas visitas é coletar e verificar marcadores imunes. A Visita I1 está programada para 13 semanas (+1 semana) após a V1; a Visita I2 para 26 semanas (+2 semanas) após a V1; a Visita I3 para 39 semanas (+3 semanas) após a V1; e, a Visita I4 para 52 semanas (+4 semanas) após a vista V1.

Procedimentos:

- Atualização das informações de contato;
- Realizar anamnese;
- Coletar amostra de sangue (amostras adicionais serão necessárias para os primeiros 60 participantes de cada faixa etária no primeiro centro de pesquisa):
- Reforçar a importância de contatar a equipe do estudo em caso de sintomas de COVID-19;

3.5.3.4 Contatos de Acompanhamento (C)

A finalidade dos contatos de acompanhamento (C) é verificar a ocorrência de eventos adversos e casos de COVID-19 entre os participantes. Estes contatos poderão ser realizados por meio eletrônico, telefônico ou presencial, a critério da equipe do estudo e do participante que informará a equipe as formas de contato de sua preferência. Os meios eletrônicos e telefônicos incluem, entre outros, envio de mensagens de texto ou áudio por telefone ou internet através de programa de computador, aplicativo para *tablet* ou telefone inteligente (*smartphone*). O contato também pode ser feito por visita domiciliar ou ao centro de pesquisa, caso seja necessário.

A frequência dos contatos será entre o terceiro e quinto dia após cada vacinação e depois disso toda semana durante as primeiras 13 semanas após a vacinação e a cada duas semanas no período restante

do estudo. Não será necessário efetuar contato nas semanas em que está programada uma vista ao centro de pesquisa.

Todo contato realizado, ou tentativa de contato com o participante, deverá ser registrado no Registro Individual do Participante. O intervalo de contato com o participante do estudo não deverá ultrapassar quatro semanas durante o período do estudo.

Procedimentos:

- Atualização das informações de contato;
- Verificar ativamente eventos febris e casos com suspeita de COVID-19;
- Verificar eventos adversos não solicitados;
- Reforçar a importância de contatar a equipe do estudo em caso de febre ou outros sintomas da COVID-19;

3.5.4 VACINAÇÃO

O médico do estudo autorizará a inclusão do participante no dia da vacinação. O farmacêutico ou enfermeiro não cego solicitará a alocação randômica pelo sistema eletrônico, conforme descrito na seção 3.10.2. O farmacêutico ou enfermeiro deve separar e cegar o produto sob investigação correspondente à alocação. O pessoal não cego não deverá ter contato com os participantes de estudo nem ter acesso aos dados de identificação do participante. Recomenda-se aplicar cada uma das doses da vacina em um braço diferente.

3.5.5 ACOMPANHAMENTO DE EVENTOS ADVERSOS NAS DUAS SEMANAS APÓS A VACINAÇÃO E USO DO "DIÁRIO DO PARTICIPANTE"

Os participantes serão instruídos a preencher um total de três "Diários de Participante" de quatorze dias cada um para cobrir o período de até quatro semanas após cada vacinação. Estes diários conterão informações relevantes sobre o estudo e nele os participantes deverão registrar a sua temperatura corporal, diariamente, por 14 dias a partir de cada vacinação e qualquer mal-estar ou sintoma, assim como medicações utilizadas. No dia 14 após a segunda vacinação, o participante irá receber o diário para preencher os eventos do dia 15 até o dia 28 após a segunda vacinação. Em resumo, os participantes deverão preencher três diários de 14 dias, um após a primeira vacinação e os outros dois diários para cobrir os 28 dias após a segunda vacinação.

A equipe do estudo fornecerá 1 (um) termômetro aos participantes e os instruirá quanto à técnica adequada para a aferição axilar da sua temperatura e solicitará que elas sejam realizadas todo dia aproximadamente no mesmo horário. Os participantes também serão orientados a aferirem a sua temperatura axilar caso considerem que estejam com febre. No caso de uma aferição compatível com febre (temperatura superior a 37,8ºC *), ela deverá ser confirmada por uma nova aferição após um intervalo de 20 minutos.

Duas semanas após a vacinação, a equipe do estudo irá revisar os registros de temperatura e outras informações escritas no "Diário do Participante" para avaliar a ocorrência de febre e eventos adversos

(conforme descrito na sessão 3.5.5). O médico fará a avaliação clínica dos potenciais eventos adversos e descreverá no registro individual do participante os eventos acontecidos no período considerando intensidade e relação causal com o produto sob investigação. Também anotará no registro individual do participante de pesquisa a temperatura mais alta registrada no intervalo entre a última visita e a visita atual. O "Diário do Participante" será recolhido pela equipe do estudo nessa visita.

** A temperatura aferida com termômetro cutâneo de scanner temporal é considerada equivalente à temperatura axilar.*

3.5.6 PROCEDIMENTOS EM CASO DE FEBRE E SUSPEITA DE COVID-19

O participante deverá ser constantemente orientado a procurar a equipe do estudo sempre que apresentar febre ou outros sintomas relacionados com COVID-19, para avaliação de ser um possível caso (ver seção 3.16.7). A equipe de estudo é responsável por estabelecer uma rotina que permite que este participante seja avaliado o mais rápido possível assim que contatar a equipe.

Em todos os casos possíveis, sempre serão coletadas amostras clínicas para detecção de SARS-CoV-2 e uma amostra de sangue para sorologia (8,5 mL). Além disso, um médico do estudo deverá fazer e registrar a avaliação clínica do participante. Cabe ressaltar que essa avaliação pode ser feita desde o segundo dia de sintomas. A apresentação clínica da COVID-19 pode variar muito e pode ocorrer concomitantemente com outras doenças, o que justifica a recomendação de se coletar amostras laboratoriais para verificar a COVID-19 em todos os casos possíveis, de forma que se possa descartar completamente o diagnóstico de COVID-19. Todos os casos possíveis devem ser acompanhados até a resolução de todos os sintomas e deve ser documentada a duração e intensidade de cada um dos sinais e sintomas. A TABELA 6 e a TABELA 7 podem ser utilizadas para classificar a intensidade desses sinais e sintomas (ver seção 3.16.3). Cada caso deve ser acompanhado para verificar a intensidade do quadro conforme à escala de progressão clínica proposta na Tabela 9. Particular atenção deve ser dada à detecção precoce de sinais de alarme e de sintomas associados com COVID-19 grave para oferecer tratamento em tempo oportuno e diminuir a intensidade das complicações clínicas. O manejo clínico dos casos seguirá as orientações das autoridades locais de saúde conforme gravidade clínica. Os casos hospitalizados deverão ter acompanhamento diário para verificar sua evolução conforme à escala de progressão clínica. Os casos ambulatoriais só terão registrada a máxima intensidade e a duração dos sintomas.

As autoridades locais de saúde podem solicitar a coleta de amostras para o diagnóstico de COVID-19. Nesse caso, haverá a coleta de uma outra bateria de amostras, diferente das amostras necessárias para estudo. Se for coletada uma outra bateria de amostras para atender as exigências legais locais, os resultados desses testes deverão ser anotados se o/a participante concordar em fornecer essas informações. Também poderá ser fornecida uma alíquota das amostras coletadas para o estudo para análises locais. De qualquer forma, os resultados do estudo estarão disponíveis para os participantes tomarem medida preventiva e relatarem esses dados às autoridades locais de saúde.

O desempenho dos testes de participantes com caso possível de COVID-19 varia de acordo com o dia de avaliação após a o início dos sintomas. Se a suspeita persistir após a obtenção de um resultado negativo para o teste, deve-se coletar uma nova amostra de clínica com, pelo menos, dois dias de intervalo. A saturação de oxigênio será medida em todos os casos para orientar a avaliação clínica do caso. Uma amostra de sangue (8,5 mL) também deverá ser coletada para avaliar parâmetros de resposta sorológicos.

3.5.7 Procedimento em caso de perda de visita ou contato

Caso o voluntário não compareça a alguma das visitas programadas ou não seja possível estabelecer o contato programado dentro do tempo estabelecido para a janela, a equipe de pesquisa continuará tentando estabelecer contato com o participante para verificar se houve algum evento adverso que impediu o participante de ir à visita ou atender ao contato e verificar se há algum caso de febre. Quando reestabelecido o contato com o voluntário que perdeu uma visita programada, uma visita extra poderá ser programada sendo coletadas as amostras correspondentes à visita mais próxima. Após essa visita, o seguimento deste retornará à rotina estabelecida no cronograma do estudo. As tentativas de contato devem ser documentadas e devem ser realizadas com frequência mínima de quatro semanas. As equipes do estudo cessarão as tentativas de contato na ausência de qualquer contato efetivo após um período de 13 semanas de tentativas.

Em caso de suspeita de evento adverso, quando retomado o contato, o participante poderá ser convocado para avaliação numa visita extra. Se não houver nenhuma preocupação em relação à segurança, o participante poderá ser avaliado na visita seguinte programada. Os relatos de evento adverso ao Comitê de Ética poderão ser apresentados mensalmente, de forma consolidada e seguindo diretrizes de cada Comitê.

3.6 Diagrama de procedimentos do estudo

O período de permanência no estudo para cada participante será de aproximadamente um ano após a vacinação, que será considerado como Dia 0 do estudo (visita V1).

Durante o estudo são previstas visitas presenciais e contatos para o acompanhamento de cada participante (**Tabela 4**).

Tabela 4 Diagrama de procedimentos do estudo.

				**Vigilância de COVID-19**				
**Tipo de visita**	**Inclusão**	**Vacinação 2**	**Segurança e imunidade**	**Segurança e imunidade**	**Imunidade**			
**Visita**	**V1**	**V2**	**SI1**	**SI2**	**I1**	**I2**	**I3**	**I4**
Programação da visita	0	V1 + 2 s (+2 s)	V2 + 2 s (+1 s)	V2 + 4 s (+1 s)	V1+ 13 s (+2 s)	V1 +26 s (+ 2 s)	V1 + 39s (+ 3 s)	V1 + 52s (+ 4 s)
**Procedimentos**								
Consentimento esclarecido*	X							
Revisão de critérios de inclusão e exclusão	X	X						
História clínica*	X	X						
Exame físico	X	X		X				
Sinais Vitais	X	X	X	X				
Peso, altura e circunferência abdominal*	X							
Revisão do risco de gravidez **	X	X						
Anamnese de eventos adversos solicitados		X		X				
Atualização de informação de contato		X	X	X	X	X	X	X
Vigilância de COVID-19 e de reações adversas não solicitadas		X	X	X	X	X	X	X
**Intervenção**								
Vacinação	X	X						
**Diários**								
Entrega de diários e termômetros	X	X	X					
Orientar aferição diária da temperatura	X	X	X	X				
Revisão de diários		X	X	X				
**Avaliação de segurança**								
β-hCG em urina **	X	X	X	X				
**Avaliação específica de SAS-CoV-2**								
Teste para detecção de ácido nucléico de SARS-CoV-2	X							
Teste qualitativo para detecção de anticorpos anti-SARS-CoV-2 por Quimiluminescência	X							
Amostras para exames de imunidade	X	X	X	X	X	X	X	X
**Volume de sangue coletado (mL)**								
Todos os participantes	**17,0**	**8,5**	**8,5**	**8,5**	**8,5**	**8,5**	**8,5**	**8,5**
Amostras adicionais para os primeiros 60 participantes de cada faixa etária no primeiro centro de pesquisa	**51,0**	**42,5**	**42,5**	**42,5**	**42,5**	**42,5**	**42,5**	**42,5**

* Pode ser realizado até três dias antes da primeira vacinação

** Para mulheres com potencial de engravidar.

3.7 CRITÉRIOS E PROCEDIMENTOS PARA SUSPENSÃO OU EXCLUSÃO DO PARTICIPANTE

A qualquer momento, se algum participante desejar sair do estudo ou se o médico do estudo considerar necessário que um participante seja excluído, a visita de exclusão incluirá os seguintes procedimentos:

- Se a exclusão ocorrer até a segunda semana após a vacinação, realizar os procedimentos da visita SI1;
- Se a exclusão ocorrer após a visita SI1, realizar os procedimentos da visita seguinte;

Um participante excluído pode retomar os procedimentos do estudo se a causa que justificou a sua exclusão for resolvida (e.g., participante que retorna à cidade após mudança temporária). Se a causa da exclusão tiver sido o desejo do próprio participante esse participante só poderá retomar os procedimentos do estudo após um novo procedimento de consentimento informado documentado através da assinatura de um novo Termo de Consentimento Livre e Esclarecido.

Caso um participante do estudo não possa receber a segunda dose da vacina por qualquer motivo, ele/ela será convidado/a permanecer no estudo para as vistas seguintes e para vigilância de casos de COVID-19. Nesse caso, a visita SI1 será programada 4 semanas (+1 sem) após a V1 e a visita SI2 ocorrerá 6 semanas (+1 sem) após a V1.

3.8 Tamanho amostral

3.8.1 Cálculo de tamanho amostral e poder estatístico

3.8.1.1 Eficácia

O tamanho amostral está direcionado a acumular suficientes casos para análises de eficácia. As hipóteses nulas (H_0) e alternativa (H_1) para a análise primária do tempo até o caso confirmado de COVID-19 nos grupos de vacinação e placebo são:

H_0: A eficácia da Vacina adsorvida COVID-19 (inativada) produzida pela Sinovac contra o caso confirmado de COVID-19 tem limite inferior do intervalo de confiança de 95% menor ou igual que 30%.

H_1: A eficácia da Vacina adsorvida COVID-19 (inativada) produzida pela Sinovac contra o caso confirmado de COVID-19 tem limite inferior do intervalo de confiança de 95% maior que 30%.

Este estudo será de grupos sequenciais fornecendo oportunidade para análises não cega de eficácia e futilidade revisadas pelo Comitê de Monitoramento de Segurança de Dados (CMDS), quando um 40% do número total de casos seja atingindo. Antes de realizar a análise interina não cega, o CMDS revisará de forma cega a taxa de casos acumulada contra a taxa de ataque assumida nos cálculos e poderá recomendar um incremento do tamanho amostral sob a premissa de que um baixo acumulo é devida a uma taxa de ataque menor que a esperada.

As seguintes premissas e limites foram utilizadas para obter o tamanho amostral:

Taxa de ataque nos 12 meses	2,5%
Eficácia vacinal (EV)	60%
EV mínima a ser demonstrada	30%
Alfa unicadual	2.5%
Poder	90%
Taxa de perda anual	5%
Número de análises interina	1
Limite superior (eficácia) da função de gasto	Parâmetro de Hwang-Shih-DeCani, = -4
Limite inferior (futilidade) da função de gasto	Parâmetro de Hwang-Shih-DeCani, = -4

Recrutamento de participantes	Uniforme durante a duração da inclusão
Razão de alocação	1:1
Tempo desejado para análise primária	12 meses

Esse desenho obteve as seguintes características:

Número de eventos para desencadear a análise interina	61
Tempo esperado para a análise interina	~7 meses
Número de eventos para desencadear a análise primária	151
Valor P para declarar eficácia na análise interina	0,0018
Valor P para declarar eficácia na análise primária	0,0241
Probabilidade, sob a hipótese alternativa, de ultrapassar o limite de eficácia na análise interina	0.2007
Probabilidade cumulativa, sob a hipótese alternativa, de ultrapassar o limite de eficácia na análise primária	0.9
Probabilidade, sob a hipótese alternativa, de ultrapassar o limite de futilidade na análise interina	0.0074
Probabilidade cumulativa, sob a hipótese alternativa, de ultrapassar o limite de futilidade na análise primária	0.1
Probabilidade, sob a hipótese nula, de ultrapassar o limite de eficácia na análise interina	0.0018
Probabilidade cumulativa, sob a hipótese nula, de ultrapassar o limite de eficácia na análise primária	0.0248
Probabilidade, sob a hipótese nula, de ultrapassar o limite de futilidade na análise interina	0.3543
Probabilidade cumulativa, sob a hipótese nula, de ultrapassar o limite de futilidade na análise primária	0.9752

A análise primária deve ser atingida nos 12 meses a partir de que o primeiro participante incluído esteja no período de seguimento duas semanas após a segunda dose. O desenho requere de que aproximadamente 13060 participantes sejam incluídos. É possível que a análise interina aconteça aproximadamente para quando esteja sendo completado o recrutamento, fornecendo ao CDMS uma oportunidade para sugerir um ajuste cego de tamanho amostral, justo antes de proceder à análise interina não cega.

Se na análise final, o critério de sucesso é atingido (p<0,0241), se espera que a razão de risco (*hazard ratio*) estimada seja aproximadamente de 50%, fornecendo uma eficácia vacinal estimada de aproximadamente 50%, com um ajuste apropriado do limite inferior do intervalo de confiança acima de 30%.

3.8.1.2 Segurança

Em relação à segurança, o cálculo de tamanho amostral foi fundamentado no denominador necessário para determinar o risco máximo de ocorrência de uma reação adversa à vacina se essa reação não for relatada no estudo de acordo com o descrito por Hanley e Lippman-Hand [35].

Fórmula 1:

$$N_i = \frac{ln\,\alpha}{\ln(1-M)}$$

Em que: N_i é o tamanho amostral de vacinados para cada faixa etária, N_1 = Adultos (18-59 anos) e N_2 = Idosos (60 anos ou mais)

α é o erro tipo I

Para o referido cálculo foram utilizados os seguintes parâmetros no grupo etário de Adultos (18-59 anos) = N_1:

- Erro tipo I igual a 5% (bicaudal)
- Risco máximo de 1 em 1000, igual a 0,001

Substituindo esses valores na Fórmula 1, obtemos $N_1 \cong 2994$.

Seguindo a proporção vacina:placebo de 1:1, 2994 participantes estarão no braço do placebo neste grupo etário, dentro de um total de Adultos N_1 = 5988. O tamanho mínimo da amostra dos Adultos em 5% para prever a perda de seguimento 5998/0,95≅6304 como a mínima quantidade de adultos (18-59 anos) no estudo.

No grupo etário de Idosos (60 anos ou mais) = N_2, os seguintes parâmetros foram utilizados:

- Erro tipo I igual a 5% (bicaudal)
- Risco máximo de 1 em 500, igual a 0,002

Substituindo esses valores na Fórmula 1, obtemos $N_2 \cong 598$.

Seguindo a proporção vacina:placebo de 1:1, 598 participantes estarão no braço do placebo neste grupo etário, dentro de um total de Idosos N_2 = 1196. No grupo dos Idosos (60 anos ou mais) também é corrigido em 5% para prever a perda de seguimento 1196/0,95≅1260, sendo esse o número mínimo dessa faixa etária no estudo, dos quais 630 receberão a vacina do estudo e 630 receberão o placebo.

3.8.2 *NÚMERO ESTIMADO DE PARTICIPANTES*

Quando considerado o número total de participantes necessários para avaliar a eficácia, 13060 participantes já contempla os números calculados para segurança. Desta forma, o número total obtido para a eficácia será o número retido para o estudo. Espera-se que até 13060 participantes ingressem no estudo, onde até 11800 sejam adultos de 18 a 59 anos e 1260 sejam idosos de 60 anos ou mais. O recrutamento de participantes pode ser modificado conforme recomendação do Comitê de Monitoramento de Segurança de Dados na ocasião da análise interina não cega ou da avaliação cega da taxa de ataque de COVID-19 durante o estudo.

3.9 POPULAÇÃO FONTE DO ESTUDO E ÁREA DE RECRUTAMENTO

A população do estudo será recrutada entre profissionais da área da saúde que trabalham em áreas especializadas no tratamento de COVID-19, pertençam ou não à instituição onde está alocado o centro de pesquisa. As pessoas interessadas poderão entrar em contato pessoalmente com o centro de estudo,

através de um telefonema, mensagem eletrônica, ou com o formulário de contato próprio, disponível na página do estudo na internet.

O estudo será divulgado através de publicidade impressa distribuída ou fixada nas áreas especializadas no tratamento de COVID-19 ou pelo uso de listas de contas eletrônicas ou de dados de contato das próprias unidades de saúde. O Instituto Butantan manterá uma página na internet com as informações de contato de todos os centros participantes da pesquisa para informar a quem desejar participar. Todos os materiais para publicidade do estudo serão encaminhados para aprovação prévia do Comitê de Ética em Pesquisa.

Existe a possibilidade de que funcionários ou estudantes das instituições de saúde ou educativas vinculadas aos centros de pesquisa que cumpram os critérios de inclusão e não tenham critérios de exclusão e se interessem em participar do estudo. Nesse caso, será verificado se o participante em potencial tem algum vínculo de dependência com o investigador principal ou com a disciplina à qual o investigador principal pertence. Nesses casos, fica assegurado o direito de recusa sem quaisquer penalidades.

3.10 Alocação de participantes

3.10.1 Geração da sequência de alocação

Haverá duas listas de randomização, uma para cada faixa etária, elaborada com base no produto em investigação a ser administrado, isto é, a vacina ou placebo em proporção de 1:1. Cada lista de randomização será feita para randomizar até 11800 Adultos e 1260 Idosos, número máximo de pessoas por faixa etária conforme descrito na Seção 3.8.2.

3.10.2 Mecanismo de ocultamento da alocação

Um sistema eletrônico de randomização central será utilizado para designar o produto sob investigação que cada participante deverá receber.

Um membro não cego, qualificado e da equipe do estudo (enfermeiro/farmacêutico) obterá a randomização correspondente, separará o respectivo produto sob investigação, cegará o produto e entregará para a equipe cega. O produto estará em uma seringa que está em um blister rotulado com o nome do patrocinador, código do produto sob investigação, via de administração, dose e data de validade do produto sob investigação. A equipe não cega do estudo não terá contato com os participantes, não terá acesso a dados de identificação ou algum outro envolvimento com o estudo, além de randomizar o participante, separar a seringa contendo placebo ou vacina, conferir se as informações da etiqueta do blister são correspondentes as informações da etiqueta do cartucho e rotular a seringa com o código do ensaio clínico, ID, visita correspondente e nome do Investigador. A opacidade do etiqueta faz com que as diferenças sejam imperceptíveis e o cegamento seja mantido.

3.10.3 Implementação da geração da sequência de alocação

Um estatístico independente, sem nenhum outro envolvimento com o estudo, irá gerar as sequências de randomização através de um programa de computador que alimentará o sistema eletrônico de randomização central. O sistema eletrônico de randomização central é um sistema validado conforme as

normas regulatórias. Este sistema informará ao membro não cego da equipe qual produto sob investigação será destinado a cada participante, sendo que a ordem de designação dos IDs seguirá a ordem de sequência da randomização e o estrato por faixa etária.

3.11 Procedimentos de cegamento

3.11.1 Descrição de quem será cego no estudo

Este estudo foi desenhado como duplo-cego para evitar a introdução de vieses na avaliação de eficácia, eventos adversos e imunogenicidade. A equipe de assistência clínica, o profissional responsável pela vacinação e o participante não saberão qual produto sob investigação será administrado. Apenas os farmacêuticos ou enfermeiros do estudo responsáveis pela randomização, separação e cegamento do produto sob investigação terão acesso às informações não cegas. A alocação do produto sob estudo será revelada unicamente após a conclusão do acompanhamento dos participantes e fechamento da base de dados para garantir a avaliação de segurança do produto a longo prazo. A equipe operacional do patrocinador também permanecerá cega.

Os resultados individuais dos ensaios experimentais de imunologia e virologia serão liberados para os centros de pesquisa somente após a abertura do cegamento com autorização por escrito do patrocinador. Caso necessário, cientistas independentes não envolvidos com a avaliação clínica ou laboratorial dos participantes, poderão analisar dados não cegos de resultados laboratoriais.

3.11.2 Procedimentos para a quebra do cegamento

A quebra precoce do cegamento de um participante, se necessária, será requerida ao patrocinador pelo pesquisador principal, ou seu representante médico, e deverá ocorrer por escrito. Isto poderá ocorrer por questões médicas emergenciais e/ou por requerimento legal e/ou regulatório. O pesquisador principal deverá documentar por escrito as razões para a quebra do cegamento e informá-las ao patrocinador em até dois dias úteis do ocorrido. A quebra do cegamento será feita através de formulário específico enviado pelo patrocinador e será restrita unicamente ao participante para o qual foi preenchido o requerimento. A quebra precoce do cegamento também deverá ser informada no registro individual do participante de pesquisa. O patrocinador deve informar uma quebra precoce do cegamento ao Presidente do Comitê de Monitoramento de Dados e Segurança (descrito na seção 3.15.1) em até dois dias úteis.

O Comitê de Monitoramento de Dados e Segurança pode quebrar o código de cegamento para avaliar os dados de segurança de um ou mais participantes. A justificativa para a quebra do código de cegamento deve ficar registrada na ata da reunião correspondente. O código que for quebrado pelo Comitê só será revelado ao patrocinador e ao investigador se for relevante para a proteção de um ou mais participantes da pesquisa.

3.12 Coleta de dados

Cada participante do estudo terá um registro individual de pesquisa que será usado como documento fonte. Nesse documento deverão ser registradas os dados das consultas médicas e de enfermagem, tais como: coletas de amostras de sangue, resultados laboratoriais entre outros documentos relacionados

com o estudo. Todos os documentos-fonte deverão ser guardados no centro de pesquisa de forma segura visando manter a confidencialidade dos participantes.

O "Diário do Participante", descrito na seção 3.5.5, será considerado documento fonte unicamente para o registro de temperaturas. Os eventos adversos e o uso de medicamentos registrados no "Diário" deverão ser informados no registro individual do participante de pesquisa por um médico do estudo.

Todas as informações relevantes para a análise de dados serão coletadas em Formulários de Relato de Caso (CRF). Informações de identificação como nome, documento de identidade, endereço, telefone, entre outras, não serão registradas em nenhum CRF. A identificação dos participantes no CRF será somente através do ID individual gerado pelo sistema. Este estudo utilizará um sistema de captura eletrônica de dados para o registro das informações nos CRFs. A entrada dos dados deverá ser feita unicamente por membros autorizados da equipe do estudo mediante o uso de senha individual com assinatura eletrônica. Os CRFs eletrônicos serão armazenados em servidor seguro designado pelo patrocinador.

3.13 PROGRAMA DE ANÁLISE E PROCESSAMENTO DE DADOS

Os dados serão registrados direta e exclusivamente pelo centro de pesquisa em CRFs eletrônicos no programa OpenClinica® Enterprise (OpenClinica, Waltham, MA, Estados Unidos), instalado localmente no servidor do patrocinador, validado e utilizado por diversas instituições americanas e europeias. A base de dados para as análises estatísticas será extraída diretamente do programa OpenClinica® Enterprise. O programa de análise estatística principal para o estudo será o R, embora outros programas estatísticos possam ser utilizados.

3.14 PLANO DE ANÁLISE DE DADOS, INCLUINDO METODOLOGIA ESTATÍSTICA

3.14.1 DEFINIÇÕES DE POPULAÇÕES A SEREM ANALISADAS

Para cumprir com os objetivos do estudo, serão avaliados diferentes conjuntos de dados conforme a natureza do desfecho.

População incluída

População incluída é definida como todos os participantes triados que assinaram termo de consentimento livre e esclarecido e são elegíveis para participar no estudo, independentemente de terem sido efetivamente randomizados ou não.

População de segurança

A população de segurança é definida como todos os participantes incluídos que receberam, ao menos, uma dose da vacina de estudo e tem dados de segurança disponíveis. Em caso de erro de alocação, o participante será analisado conforme o produto que tenha recebido efetivamente.

População para avaliação de reatogenicidade

A população para avaliação de reatogenicidade é definida como todos os participantes da população de segurança que recebam e retornem um Diário do participante para avaliação de eventos adversos

solicitados. A população é definida para cada vacinação (primeira ou segunda dose) e precisa que o participante tenha efetivamente recebido a dose correspondente.

População Por-Protocolo (PP)

A população Por Protocolo é definida como todos os participantes randomizados que: cumpriram os critérios de inclusão (Seção 3.2.1) e não preencheram nenhum critério de exclusão (Seção 3.2.2); receberam as duas doses do produto sob investigação, ao qual foram alocados, nas condições de manipulação e administração recomendadas pelo fabricante (Seção 3.4.1.1.2) e não utilizaram medicações restritas conforme o protocolo (Seção 3.4.2).

Os participantes permanecerão na População Por Protocolo até que aconteça um desvio importante (ex. receber outra vacina para COVID-19) e esses contribuíram no tempo de seguimento para as análises por protocolo até o evento de exclusão. Um relatório detalhará os participantes excluídos, o momento de exclusão e a justificativa. Se o participante não tiver nenhum desvio de protocolo relevante, contribuirá até a finalização de seu acompanhamento. A análise Por Protocolo é a análise principal de eficácia do estudo.

População por Intenção de Tratar

A população por Intenção de Tratar incluirá a todos os participantes randomizados que receberem pelo menos uma dose do produto. Os participantes serão analisados no grupo para o qual foram alocados e contribuíram com o tempo de seguimento que estiver disponível para a análise por Intenção de Tratar. A análise por Intenção de Tratar é considerada uma análise secundária de eficácia para este estudo.

Populações para imunogenicidade

As populações para imunogenicidade correspondem a todos os participantes da população Por Protocolo que tenham as amostras correspondentes para a realização das análises correspondentes.

Para a análise de imunidade celular, a população está restrita aos 60 primeiros participantes de cada faixa etária no primeiro centro de pesquisa. Para as análises de imunidade humoral e correlatos de proteção, todos os participantes que tenham amostras disponíveis podem contribuir.

3.14.2 Metodologia Descritiva

Todos os dados coletados serão resumidos e/ou listados. As estatísticas descritivas incluem média, desvio padrão, mediana e valores mínimos e máximos para variáveis continuas; e números e proporções por grupo para as variáveis categóricas. Exceto quando seja especificado de outra forma, um valor de significância de 5% bicaudal será utilizado para testes estatísticos e intervalos de confiança. Os intervalos de confiança exatos serão usados para resumos univariados de variáveis dicotômicas, e intervalos de confiança baseados em score para diferenças entre taxas. Todas as proporções terão como denominador o número de participantes contribuído com dados no tempo especificado dentro do grupo especificado e a população de estudo especificada. Os resumos serão apresentados por grupo e por tempo, quando for relevante.

Os dados basais demográficos, antropométricos e de exposição prévia a SARS-CoV2 serão apresentados de forma descritiva. Em relação a raça ou origem étnica, será considerada a autodeclaração do

participante. As análises poderão ser realizadas conforme faixa etária e grupo, usando o Teste exato de Fisher para variáveis binárias e análise de variância (ANOVA) para as variáveis continuas.

Para listar e resumir medicações usadas pelos participantes será utilizado o Dicionário de Medicações da OMS tabuladas por classificação ATC, grupo de tratamento e nome preferencial. O histórico médico e as reações adversas serão codificadas com MedDRA e tabuladas por Classe de órgão e sistema (SOC), termo preferencial (PT) e grupo de alocação.

A distribuição dos participantes incluindo os números de pessoas incluídas, triadas, randomizadas e vacinadas será resumida e apresentada no diagrama CONSORT, incluindo descontinuação do estudo. As causas de falha na triagem e descontinuação serão descritas.

3.14.3 Análise primário de eficácia

A análise principal de eficácia será uma análise modificada por protocolo calculada com todos os casos confirmados virologicamente de COVID-19 que ocorrerem no período com início duas semanas após a segunda vacinação.

A eficácia vacinal entre os participantes com sintomas de COVID-19 com detecção de ácido nucléico de SARS-CoV-2 em amostra clínica será avaliada usando regressão de riscos proporcionais de Cox, estratificado por faixa etária e sexo, com uma covariável de grupo de alocação para comparar os que receberam vacina com os que receberam placebo. Esse modelo calcula a eficácia vacinal estimada (1 – *hazard ratio*), intervalo de confiança 95% e os valores de p. Também serão criados gráficos de incidência cumulativa com esse modelo. Essa análise será realizada na população Por Protocolo, como análise primaria. Na população por Intenção de Tratar será considerada como análise secundária

O teste de hipótese do desfecho primário de eficácia na população Por Protocolo será baseado nos níveis alfa a serem gastos em cada análise e acompanhada com os intervalos de confiança correspondentes. Se eventos adicionais são acumulados após o evento final necessário para a análise primária, mas antes de fechamento da base de dados, uma análise final será realizada para incorporar esses eventos.

Os desfechos secundários de eficácia serão avaliados da mesma forma que o desfecho primário, prevendo ajustes para a avalição sequencial. A avaliação do desfecho de eficácia por estrato de randomização (faixa etária) será feita de forma análoga.

Para cada desfecho de eficácia, a eficácia vacinal cumulativa por tempo, definida como 100% × (1- razão de incidência cumulativa por unidade de tempo), será colocada em gráfico com os pontos de avaliação temporais e cálculo do intervalo de confiança 95% simultâneo com o método de Parzen, Wei e Ying.

3.14.4 Análises de segurança

As análises de segurança incluirão todos os participantes que receberam o produto sob investigação conforme o produto recebido, por grupo e faixa etária. A análise primária de segurança considerará todos os eventos adversos, solicitados e não solicitados, com relação causal razoável com o produto acontecidos na primeira semana após sua administração de acordo com o grupo etário. A análise secundária estenderá esse período até a quarta semana após a vacinação. Adicionalmente, serão incluídos em análises subsequentes os eventos adversos não solicitados com relação causal razoável com o produto após a quarta semana após a administração do produto até o fim acompanhamento na semana 52. A

frequência e intensidade de eventos e reações adversas serão relatadas para cada dose do produto sob investigação.

Todos os eventos adversos serão codificados e agrupados conforme a metodologia MedDRA (*Medical Dictionary for Regulatory Activities*).

Os eventos adversos solicitados serão analisados pela proporção de participantes com observação de algum evento e conforme o grau e tipo de evento nesse grupo. O conjunto de participantes analisados será correspondente ao conjunto de participantes dos quais existem dados, ex. para observação imediata após a vacinação deve ser confirmado que o participante foi observado durante os 60 minutos após a vacinação. A população para avaliação de reatogenicidade será a analisada para os eventos até a primeira e quarta semana, conforme a disponibilidade do Diário do Participante. Os eventos adversos solicitados registrados serão apresentados de forma resumida conforme sua máxima intensidade e duração por participante, quando relevante. As taxas serão acompanhadas de intervalos de confiança 95% exatos bicaudais. Entre os grupos, as taxas serão comparadas usando Teste exato de Fisher bicaudal e o Teste de Cochran-Mantel-Haenszel conforme seja mais apropriado para comparações pareadas, tanto em geral como por tipo e por intensidade.

Os eventos adversos não solicitados analisados serão aqueles acontecidos dentro dos 28 dias após uma vacinação, exceto quando sejam considerados Eventos Adversos Graves ou que tenham ralação causal razoável com a vacinação. Os eventos adversos não solicitados serão resumidos a nível de participante onde um participante contribui uma só vez para um tipo de evento dado sob a máxima intensidade e/ou causalidade. Tabelas adicionais podem resumir o número de eventos de um determinado tipo observado num grupo, sem considerar o número de participantes que o originou.

Os eventos adversos não solicitados serão resumidos por intensidade e relação causa com a vacinação. No caso de Eventos Adversos Graves, também será registrado critério para serem classificado s como graves. As tabelas apresentarão os eventos adversos não solicitados com frequência de 1% ou mais. Tabelas adicionais mostraram eventos que levaram a descontinuação da vacinação, que tiveram intensidade 3 ou 4 e eventos adversos graves. Entre as faixas etárias, as taxas de eventos adversos com relação causal com a vacinação serão comparadas usando Teste exato de Fisher bicaudal.

Os casos de COVID-19 e de Síndrome Respiratório Agudo Grave serão comparados entre os grupos de alocação tanto em sua frequência como na sua intensidade. O Score de intensidade será comparado pelo teste de Wilcoxon e a frequência de casos graves do total de eventos será comparada para verificar diferença de proporções entre os grupos. Essas comparações têm como finalidade avaliar se existe alguma possibilidade de doença potenciada pela vacina. Um CRF separado coletará os sintomas para determinar doença respiratória e a intensidade de esses sintomas para as comparações entre os grupos assim como por faixa etária e sexo.

Dados de linha de base como presença de comorbidades também serão apresentados entre os grupos. Todos os casos de gravidez também serão descritos, incluindo os desfechos.

3.14.5 ANÁLISES DE IMUNOGENICIDADE

A análise irá descrever os resultados de imunogenicidade de um subgrupo de participantes de cada grupo etário em termos de soroconversão de anticorpos neutralizantes. As médias geométricas dos títulos também serão descritos entre aqueles com soroconversão e serão comparados entre aqueles que adquiriram a infecção e um subgrupo daqueles sem infecção. Estas informações serão elucidadas à luz do

braço correspondente, seja o de vacina ou o de placebo. As análises consideraram a presença de títulos de anticorpos antes da primeira vacinação e o relato documentado de infecção prévia.

As seguintes estatísticas descritivas serão calculadas para cada avaliação e cada grupo:

- Taxas de soroconversão com intervalos de confiança 95% comparando contra o título basal
- Mediana dos títulos com intervalos de confiança 95%
- Gráficos da distribuição cumulativa reversa de títulos / concentrações
- Gráficos de caixas das distribuições de títulos / concentrações

3.14.6 GERENCIAMENTO DE DADOS FALTANTES/AUSENTES

Não serão imputados dados para a análise primária de eficácia por protocolo. Portanto, as análises excluíram participantes com dados faltantes ou não avaliáveis. Se houver uma quantidade de dados faltante excessiva ou houver algum tipo de padrão entre os dados faltantes, ferramentas estatísticas adicionais podem ser implementadas.

3.15 MONITORAMENTO DE DADOS DO ESTUDO

3.15.1 COMITÊ DE MONITORAMENTO DE DADOS E SEGURANÇA

Este estudo terá um Comitê de Monitoramento de Dados e Segurança constituído com base nas recomendações do Ministério de Saúde [36]. Este comitê será nomeado pelo patrocinador e estará composto por três pessoas com uma das seguintes qualificações:

- Médico Infectologista: deve ter experiência na realização de estudos relacionados com o objeto do estudo.
- Epidemiologista: deve ter experiência na realização de estudos ou em vigilância epidemiológica relacionados com objeto do estudo.
- Estatístico: Deve ter graduação ou pós-graduação em estatística. Deve ter experiência em análise de dados na área da saúde.
- Outros profissionais da área da saúde: devem ter experiência no desenho, condução ou na análise de ensaios clínicos.

Os membros do comitê serão totalmente independentes do patrocinador. Não há previsão de pagamento de honorários para os membros do comitê. Funcionários do patrocinador poderão dar apoio às atividades do comitê, mas não poderão fazer parte do mesmo.

O comitê fará recomendações por escrito para continuar, modificar, suspender ou encerrar o estudo. Essas recomendações serão recebidas pelo patrocinador e comunicadas aos investigadores para que eles possam encaminhá-las aos respectivos Comitês de Ética em Pesquisa. O patrocinador poderá discordar das recomendações através de resposta justificada que será comunicada da mesma forma.

3.15.2 ANÁLISES E REVISÃO INTERINA

O Comitê de Monitoramento de Dados e Segurança terá acesso aos dados referentes à segurança dos participantes, inclusive aos resultados dos testes laboratoriais durante todo o estudo e será convocado para a análise de segurança do cego:

- Quando a informação de segurança das duas semanas após a primeira vacinação dos primeiros 450 participantes do grupo de Adultos (18-59 anos) estiver disponível;
- Quando a informação de segurança das duas semanas após a primeira vacinação dos primeiros 450 participantes do grupo dos Idosos (60 anos ou mais) estiver disponível;
- Quando a informação de segurança das quatro semanas após a segunda vacinação de todos os participantes do grupo dos Idosos (60 anos ou mais) estiver disponível;
- Quando a informação de segurança das duas semanas após a primeira vacinação dos primeiros 1350 participantes do grupo dos Adultos (18 a 59 anos) estiver disponível;
- Quando a informação de segurança das quatro semanas após a segunda vacinação de todos os participantes no grupo dos Adultos (18 a 59 anos) estiver disponível.

Após as reuniões de análise de segurança descritas acima, outras reuniões serão acordadas com periodicidade mínima de seis meses, conforme decisão do presidente do comitê.

Caso o Comitê Independente de Monitoramento de Dados e Segurança considere necessário, este poderá quebrar precocemente o cegamento de qualquer participante durante a realização do estudo para acessar a segurança. Após cada reunião, o Comitê Independente de Monitoramento de Dados e Segurança deverá emitir um relatório para o patrocinador indicando a continuidade ou suspensão da administração do produto sob investigação acompanhado ou não de recomendações adicionais para a proteção dos participantes do estudo. Análises interinas de eficácia, segurança e imunogenicidade, sem quebra de cegamento da alocação de participantes individuais, podem fazer parte dos relatórios do Comitê. Após todos os participantes receberem a última vacinação, os relatórios deverão conter as recomendações consideradas necessárias para manter a proteção dos participantes do estudo.

O Comitê Independente de Monitoramento de Dados e Segurança pode realizar análises interinas de eficácia e imunogenicidade não cegas antes da conclusão do estudo quando cumpridas as seguintes condições:

- Quando os dados de segurança relativos à primeira semana após a segunda vacinação de, ao menos, 6000 participantes do grupo de Adultos (18 a 59 anos) estiverem disponíveis;
- O número de casos de COVID-19 virologicamente confirmados estiver acima do estipulado na seção 3.8.1.1, que corresponde a 61 casos.

Caso a análise interina de eficácia demonstre eficácia da vacina conforme os critérios estatísticos da seção 3.8.1.1, os resultados serão anunciados às autoridades éticas e regulatórias e o esquema de vacinação será oferecido na visita I1 para os participantes que receberam placebo. Se os resultados demonstrarem

futilidade conforme os critérios estatísticos da seção 3.8.1.1, o recrutamento do estudo será interrompido se ainda estiver em andamento.

3.15.3 Critérios para suspensão ou interrupção do estudo

Se a reatogenicidade da vacina for considerada inaceitável, ou se houver evidências precoces de eficácia muito alta ou muito baixa, a sua administração será suspensa até que o Comitê Independente de Monitoramento de Dados e Segurança e o patrocinador revisem os dados disponíveis.

A administração do produto sob investigação será retomada se a revisão realizada pelo Comitê Independente de Monitoramento de Dados e Segurança e pelo patrocinador resultar em tal recomendação. Se a administração do produto for encerrada na ocasião das ocorrências acima descritas, o Comitê poderá revisar os dados e fazer recomendações para acompanhamento dos participantes.

Os relatórios com os dados de segurança e as modificações na situação da administração do produto sob investigação (suspensa/retomada) serão submetidos ao Comitê de Ética em Pesquisa de acordo com as políticas institucionais.

Estes critérios são considerados como mínimos e a decisão de suspender a administração do produto sob investigação em qualquer um dos centros de estudo poderá ser tomada, com base em qualquer outro critério relevante no julgamento do investigador e/ou do Comitê de Ética em Pesquisa. Da mesma forma, as autoridades regulatórias ou o Patrocinador poderão suspender a administração do produto caso exista uma preocupação importante quanto à segurança da vacina.

3.16 Avaliação da segurança

3.16.1 Definições

As definições de eventos adversos seguem as recomendações das Boas Práticas Clínicas e do Guia E2A sobre Manejo de Dados Clínicos de Segurança da Conferência Internacional de Harmonização (ICH) [37].

3.16.1.1 Evento Adverso

Neste estudo, evento adverso será definido como qualquer ocorrência médica desfavorável que ocorra em um participante que foi vacinado e que não necessariamente tenha uma relação causal com a administração da vacina ou do placebo. Portanto, um evento adverso pode ser qualquer sinal, sintoma ou doença desfavorável e não intencional (incluindo uma descoberta anormal em exame laboratorial), temporalmente associado com o produto da vacinação, seja ele considerado ou não como relacionado com o produto da vacinação.

3.16.1.2 Evento Adverso Grave

Neste estudo, um Evento Adverso Grave (EAG) será definido como qualquer evento adverso que resulte em qualquer um dos seguintes desfechos [38]:

- Óbito.
- Ameaça à vida: há risco de morte no momento do evento.

- Hospitalização ou prolongamento de hospitalização já existente: hospitalização é um atendimento hospitalar com necessidade de internação. Também inclui um prolongamento da internação devido a um evento adverso.

- Incapacidade significativa ou persistente: é uma interrupção substancial da habilidade de uma pessoa conduzir as funções de sua vida normal.

- Anomalia congênita.

- Qualquer suspeita de transmissão de agente infeccioso por meio de um medicamento.

- Evento clinicamente significante: é qualquer evento decorrente do uso de medicamentos que necessitam intervenção médica, a fim de se evitar óbito, risco à vida, incapacidade significativa ou hospitalização.

Um evento adverso precisa preencher apenas um dos critérios acima para ser considerado como um Evento Adverso Grave. A classificação de um evento adverso em Evento Adverso Grave não depende da sua relação causal com o produto da vacinação.

3.16.1.3 Reação Adversa

Neste estudo, serão considerados como reações adversas todos os eventos adversos que apresentarem uma relação causal razoável com o produto sob investigação.

3.16.1.4 Condições pré-existentes/Piora de condição pré-existente

Condições médicas estáveis vigentes na visita de inclusão do participante devem ser informadas no registro individual do participante de pesquisa e não serão consideradas eventos adversos. A piora de uma condição pré-existente será definida como um evento adverso e deverá ser avaliada como tal. Um evento adverso decorrente da piora de uma condição pré-existente será considerado como resolvido quando a condição pré-existente retornar a sua condição basal registrada na visita de inclusão.

3.16.2 MONITORAMENTO DOS EVENTOS ADVERSOS

O investigador principal ou seu/sua representante médico deverá incluir na sua avaliação de eventos adversos:

- A classificação da sua gravidade (ver seção 3.16.1.2);

- A classificação da sua intensidade (ver seção 3.16.3);

- A classificação da sua associação causal com produto sob investigação (ver seção 3.16.4);

- As medidas tomadas;

- A sua evolução.

Após a vacinação, todos os participantes serão questionados quanto à ocorrência de sinais e sintomas específicos (eventos adversos solicitados) e também quanto à ocorrência de sinais e sintomas adicionais relatados de forma espontânea (eventos adversos não solicitados).

3.16.3 Classificação da intensidade

A intensidade dos eventos adversos clínicos solicitados será classificada através de uma escala numérica de 1 até 4, conforme a TABELA 5 (eventos locais) e a TABELA 6 (eventos sistêmicos), criada com base nos guias *"Toxicity Grading Scale for Healthy Adult e Adolescent Volunteers Enrolled in Preventive Vaccine Clinical Trials"* da Administração de Medicamentos e Alimentos dos Estados Unidos (FDA) e no guia "*Common Terminology Criteria for Adverse Events - Version 5.0"* do Instituto Nacional de Câncer dos Estados Unidos (NCI/NIH).

Tabela 5 Classificação da intensidade dos eventos adversos clínicos locais solicitados

**Evento Adverso**	**Grau 1**	**Grau 2**	**Grau 3**	**Grau 4**
Dor no local da administração do produto sob investigação	Não interfere nas atividades diárias	Uso repetido de analgésico não narcótico >24 horas OU Interfere pouco nas atividades diárias	Qualquer uso de analgésico narcótico OU Impede atividades diárias	Visita ao pronto socorro* OU Hospitalização
Eritema no local da administração do produto sob investigação†	25 – 50 mm	51 – 100 mm	> 100 mm	Necrose OU Dermatite esfoliativa
Inchaço no local da administração do produto sob investigação	25 – 50 mm	51 – 100 mm OU Interfere nas atividades diárias	> 100 mm OU Impede atividades diárias	Necrose
Induração no local da administração do produto sob investigação	25 – 50 mm	51 – 100 mm OU Interfere nas atividades diárias	> 100 mm OU Impede atividades diárias	Necrose
Prurido no local da administração do produto sob investigação	Não interfere nas atividades diárias	Interfere nas atividades diárias	Impede atividades diárias	Visita ao pronto socorro* OU Hospitalização

* Requer 12 horas ou mais de internação em enfermaria ou pronto socorro para manejo do evento adverso
† O valor registrado deve ser o aferido no local de maior diâmetro e como uma variável contínua.

Tabela 6 Classificação da intensidade dos eventos adversos clínicos sistêmicos solicitados e dos sinais e sintomas no caso de febre e suspeita de COVID-19.

**Evento Adverso**	**Grau 1**	**Grau 2**	**Grau 3**	**Grau 4**
Febre	37.8°C – 38.4°C	38.5°C – 38.9°C	39.0°C – 40.0°C	>40°C

**Evento Adverso**	**Grau 1**	**Grau 2**	**Grau 3**	**Grau 4**
Náusea	Não interfere nas atividades diárias<br>OU<br>1 a 2 episódios em 24 horas	Interfere pouco nas atividades diárias<br>OU<br>Mais de 2 episódios em 24 horas	Impede atividades diárias, requer hidratação intravenosa	Visita ao pronto socorro*<br>OU<br>Hospitalização<br>OU<br>Choque hipovolêmico
Vômito	Não interfere nas atividades diárias<br>OU<br>1 a 2 episódios em 24 horas	Interfere pouco nas atividades diárias<br>OU<br>Mais de 2 episódios em 24 horas	Impede atividades diárias, requer hidratação intravenosa	Visita ao pronto socorro*<br>OU<br>Hospitalização<br>OU<br>Choque hipovolêmico
Diarreia	2 – 3 fezes soltas em 24 horas	4 – 5 fezes em 24 horas	6 ou mais fezes liquefeitas ou requer hidratação IV em pronto atendimento	Visita ao pronto socorro*<br>OU<br>Hospitalização
Dor de cabeça	Não interfere nas atividades diárias	Uso repetido de analgésico não narcótico >24 horas ou interfere pouco nas atividades diárias	Qualquer uso de analgésico narcótico ou impede atividades diárias	Visita ao pronto socorro*<br>OU<br>Hospitalização
Fadiga	Não interfere nas atividades diárias	Interfere pouco nas atividades diárias	Impede atividades diárias	Visita ao pronto socorro*<br>OU<br>Hospitalização
Mialgia	Não interfere nas atividades diárias	Interfere pouco nas atividades diárias	Impede atividades diárias	Visita ao pronto socorro*<br>OU<br>Hospitalização
Calafrios	Sensação leve de frio; calafrios, dentes batendo	Sensação moderada de corpo inteiro tremendo, requer uso de opioides	Grave ou prolongado, não responde a opioides	-----
Anorexia	Perda de apetite sem alteração dos hábitos alimentares	Consumo oral alterado sem significativa perda de peso ou má nutrição; suplementos nutricionais orais indicados	Associada a significativa perda de peso ou má nutrição (ex. consumo inadequado de calorias e/ou fluidos orais); alimentação por tubo ou nutrição parenteral completa indicada	-----
Tosse	Sintomas leves; intervenção sem prescrição indicada	Sintomas moderados, intervenção médica indicada; limita atividades instrumentais da vida diária	Sintomas graves; limita atividades de auto cuidado da vida diária	-----

Evento Adverso	Grau 1	Grau 2	Grau 3	Grau 4
Artralgia	Dor leve	Dor moderada; limita atividades instrumentais da vida diária	Dor grave; limita atividades de auto cuidado da vida diária	-----
Prurido	Leve ou localizado. Pode precisar uso de medicação tópica	Intenso ou generalizado; intermitente. Mudanças na pele devidas à coceira (edema, pápulas, escoriações, liquenificação, crostas). Interfere nas atividades diárias. Pode precisar de medicação oral	Intenso ou generalizado; intermitente. Impede autocuidado, atividades diárias ou sono. Pode precisar uso de corticosteroide sistêmico ou imunossupressor.	-----
Erupção cutânea (exantema)†	Presente, mas assintomático	Sintomático (prurido/dor), mas interfere pouco nas atividades diárias	Sintomático, impede atividades diárias	Visita ao pronto socorro* OU Hospitalização
Reação alérgica	Intervenção sistêmica não indicada	Intervenção oral indicada	Broncoespasmo; hospitalização indicada para sequelas clínicas; intervenção intravenosa indicada	Consequências de risco à vida; intervenção urgente indicada

* Requer 12 horas ou mais de internação em enfermaria ou pronto socorro para manejo do evento adverso.
† Especificar se a erupção cutânea é localizada em alguma região do corpo ou se é generalizada.

A intensidade dos eventos adversos clínicos não solicitados será classificada através de uma escala numérica de 1 até 4, conforme a TABELA 7, criada com base no guia "*Toxicity Grading Scale for Healthy Adult e Adolescent Volunteers Enrolled in Preventive Vaccine Clinical Trials*" da Administração de Medicamentos e Alimentos dos Estados Unidos (FDA) e no guia "*Common Terminology Criteria for Adverse Events - Version 5.0*" do Instituto Nacional de Câncer dos Estados Unidos (NIC/NIH).

Tabela 7 Classificação da intensidade dos Eventos Adversos clínicos não solicitados e de outros sinais e sintomas no caso de febre e suspeita de COVID-19

Evento Adverso	Grau 1	Grau 2	Grau 3	Grau 4
Frequência respiratória	17-20 respirações por minuto	21-25 respirações por minuto	>25 respirações por minuto	Intubação
Dispneia	Falta de ar com esforço moderado	Falta de ar com esforço mínimo; limitante para atividades instrumentais da vida diária	Falta de ar em repouso; limitante para atividades de auto cuidado da vida diária	Consequências de risco de vida; indicada intervenção urgente
Congestão nasal	Sintomas leves; intervenção não indicada	Sintomas moderados; intervenção médica indicada	Associada a secreção nasal com sangue ou epistaxe	
Anosmia	Presente	-----	-----	-----

Evento Adverso	Grau 1	Grau 2	Grau 3	Grau 4
Disgeusia / Ageusia	Paladar alterado, mas sem alteração da dieta	Paladar alterado com alteração da dieta (ex., suplementos orais); paladar desagradável ou nocivo; perda do paladar	-----	-----
Doença ou evento adverso clínico (conforme definido pelas normas que se aplicam) e outros sinais e sintomas	Não interfere nas atividades	Interfere pouco nas atividades e não requer intervenção médica	Impede atividades diárias e requer intervenção médica	Visita ao pronto socorro* OU Hospitalização

* Requer 12 horas ou mais de internação em enfermaria ou pronto socorro para manejo do evento adverso.

Todos os casos de óbito terão sua intensidade classificada como Grau 4.

A maior intensidade relatada para um evento adverso, até a sua resolução ou desfecho, será a intensidade utilizada para a realização das análises do estudo.

3.16.4 Classificação da relação causal

Todos os eventos adversos deverão ser classificados pelo pesquisador principal ou pelo seu representante médico quanto à sua relação causal com o produto sob investigação, conforme a classificação adaptada do *"Uppsala Monitoring Centre"* da Organização Mundial da Saúde (WHO-UMC) [39], descrita na TABELA 8.

O patrocinador poderá solicitar esclarecimentos adicionais ao pesquisador principal para justificar a relação causal atribuída ao evento. A relação causal também poderá ser revisada por solicitação do Comitê Independente de Monitoramento de Dados e Segurança mediante justificativa por escrito.

Todas as reações locais após a administração do produto sob investigação serão consideradas como eventos adversos com relação causal certa à vacinação.

Tabela 8 Classificação da relação causal dos Eventos Adversos com o produto sob investigação

**Relação causal razoável**			**Relação causal NÃO razoável**	
Evento Adverso considerado como Reação Adversa			O Evento Adverso não pode ser considerado como Reação Adversa.	
**Certa**	**Provável**	**Possível**	**Improvável**	**Não relacionado**
Evento ou alteração (valor anormal) em exame laboratorial, com relação temporal plausível em relação à administração da intervenção;	Um evento clínico, incluindo uma alteração (valor anormal) em exame laboratorial, com relação temporal razoável em relação à administração da intervenção;	Um evento clínico, incluindo uma alteração (valor anormal) em exame laboratorial, com relação temporal razoável em relação à administração da intervenção;	Um evento clínico, incluindo uma alteração (valor anormal) em exame laboratorial, que devido ao momento da administração da intervenção faz uma relação improvável, mas não impossível;	Um evento clínico, incluindo uma alteração (valor anormal) em exame laboratorial, que devido ao momento da administração da intervenção faz com que não exista uma relação;
Não pode ser explicado por doença concomitante ou outra intervenção ou medicamento;<br><br>O evento é definido farmacologicamente ou fenomenologicamente (i.e., uma desordem objetiva e específica ou um fenômeno farmacologicamente reconhecido);	Improvável que seja atribuído à uma doença concomitante ou à outra intervenção ou medicamento;	Também pode ser explicado por doença concomitante ou outras intervenções ou medicamentos;	Outra doença ou outro medicamento fornecem uma explicação plausível	Outra doença ou outro medicamento fornecem uma explicação plausível
A resposta à interrupção ou retirada é plausível (farmacologicamente, patologicamente);	A resposta à interrupção ou retirada é clinicamente razoável;	Falta de informação ou ainda falta de clareza sobre a retirada ou interrupção do tratamento		
Reexposição satisfatória, se necessário	Reexposição não exigida			

3.16.5 *IDENTIFICAÇÃO E RELATO DOS EVENTOS ADVERSOS*

O monitoramento da segurança neste estudo incluirá avaliações clínicas e laboratoriais dos participantes, abrangendo eventos adversos locais e sistêmicos, solicitados e não solicitados.

Inicialmente, os participantes serão observados no centro de estudo por 60 minutos após cada vacinação e serão instruídos para registrar qualquer reação adversa nas primeiras três semanas após a vacinação, sendo que visitas adicionais poderão ser agendadas, se necessárias. Espera-se que eventos adversos razoavelmente relacionados ocorram com maior frequência nas primeiras duas semanas após a vacinação.

Na visita V2, no dia da segunda vacinação, e nas visitas SI1 e SI2, duas e quatro semanas após a segunda vacinação, os pesquisadores responsáveis pela avaliação clínica e laboratorial dos participantes checarão os registros no “Diário do Participante”, irão questioná-los sobre a ocorrência de possíveis eventos adversos e realizarão um exame físico. Os participantes poderão contatar um médico do estudo 24 horas por dia durante todo o período do estudo através de um número de telefone.

3.16.5.1 Relato dos Eventos Adversos

Todos os eventos adversos que forem identificados durante o estudo devem ser registrados em um documento fonte. Os eventos adversos acontecidos nos primeiros 28 dias após a vacinação e aqueles acontecidos em qualquer momento que tenham relação causal razoável com o produto sob investigação deverão ser relatados ao patrocinador através do formulário de relato de caso (CRF) de evento adverso, em até sete dias corridos do seu conhecimento por algum membro da equipe do estudo. As atualizações sobre um evento adverso devem ser registradas assim que novas informações estiverem disponíveis até o seu desfecho (recuperado, em recuperação, recuperado com sequela, perda de seguimento, não recuperado ou morte).

3.16.5.2 Relato Urgente de Eventos de Interesse Especial

O Relato Urgente de Eventos de Interesse Especial tem como objetivo informar ao patrocinador, a outros investigadores e aos órgãos regulatórios sobre novos dados a respeito de reações graves ou ocorrências que necessitem de um monitoramento especial. A Lista Prioritária de Eventos Adversos de Interesse Especial em vacinas COVID-19 elaborada pela Brighton Collaboration [40] está incluída nos eventos de interesse especial. Neste estudo, devem ser relatados de forma urgente:

- Eventos Adversos Graves (ver seção 3.16.1.2);
- Caso confirmado de COVID-19, incluindo casos graves (ver seções3.16.7.1 e 3.16.7.4);
- Gestação nas primeiras 4 semanas após a última vacinação;
- Convulsão generalizada;
- Síndrome de Guillain-Barré;
- Encefalomielite aguda disseminada;
- Trombocitopenia hematológica;
- Anafilaxia imunológica;
- Vasculites;
- Outros eventos adversos graves locais ou sistêmicos após imunização.

Todos os Eventos de Interesse Especial deverão ser relatados ao Patrocinador em até um (1) dia corrido a partir da data de conhecimento do evento pela equipe do centro de pesquisa. A notificação inicial não deve ser prorrogada mesmo que as informações estejam incompletas. O relato de Eventos Adversos Graves será preenchido preferencialmente através de formulário específico que está integrado no sistema de CRF após avaliação por um médico do estudo. Na impossibilidade de acessar o CRF, a notificação deverá ser feita através de formulário específico enviado por correio eletrônico ou ligação telefônica à equipe da Divisão de Ensaios Clínicos e Farmacovigilância do Patrocinador e será registrada assim que possível no formulário integrado no sistema de CRF acima citado referenciando data e hora de contato da primeira notificação acontecida por qualquer meio.

Para os Eventos Adversos Graves e casos de COVID-19 prováveis ou confirmados, atualizações deverão ser enviadas a cada duas semanas, ou assim que novas informações estiverem disponíveis até o seu desfecho final (recuperado, em recuperação, recuperado com sequela, perda de seguimento, não recuperado ou morte) e se houve ou não, necessidade de hospitalização. Para as gestações em andamento nas primeiras 13 semanas de estudo, as atualizações deverão ser enviadas periodicamente até o seu desfecho (parto, aborto ou óbito fetal) para garantir a verificação da ocorrência ou não de Eventos Adversos Graves. As voluntárias que tiverem gestação detectada nas primeiras 4 semanas após a última vacinação serão encaminhadas para acompanhamento em serviço especializado de Obstetrícia, e acompanhadas até o desfecho da gestação e até 6 semanas após o parto, se necessário.

O CRF com as informações sobre Eventos Adversos Graves deverá ser preenchido e atualizado em todos os casos com as informações avaliadas por um médico do estudo, independentemente da forma de notificação inicial do evento. Quando um Evento Adverso Grave tiver sua relação causal com o produto sob investigação classificada como não razoável, uma etiologia, diagnóstico ou explicação alternativa deve ser fornecida. Neste estudo, os seguintes tipos de hospitalização não geram um Relato Urgente:

a. Qualquer admissão não relacionada com um evento adverso (e.g., parto, cirurgia cosmética ou programada);

b. Qualquer admissão para diagnóstico ou terapia de uma condição clínica que existia antes da administração do produto sob investigação do estudo e que não teve sua frequência ou intensidade aumentada, segundo o julgamento de um dos médicos do estudo;

c. Gestações em que o dia da concepção seja posterior ao Dia 28 pós-imunização; seja determinada por um teste de β-hCG negativo, em urina ou sangue, realizado no Dia 28 ou posterior e anterior à gravidez, ou por cálculo de idade gestacional realizado durante o acompanhamento da gravidez.

3.16.6 PERÍODO DE AVALIAÇÃO DA SEGURANÇA

Todos os eventos adversos solicitados e não solicitados identificados durante as primeiras quatro semanas (28 dias) após a vacinação deverão ser relatados independentemente de sua relação de causalidade com os produtos sob investigação. Os eventos adversos não solicitados com relação causal razoável com o produto de investigação conforme o critério do investigador e/ou o patrocinador deverão ser relatados a qualquer momento durante o estudo. Os eventos adversos graves, independentemente de sua relação causal, devem ser relatados a qualquer momento durante o estudo ou até após estudo.

Gestações em que que seja comprovado que a concepção ocorreu a partir da quarta semana após a última imunização não serão acompanhadas para avaliar eventos adversos graves uma vez que esta concepção

terá acontecido após eliminação da vacina. Os casos de COVID-19 serão relatados durante toda a duração do estudo.

3.16.7 Definições de caso de COVID-19

A definição de vigilância de caso para a doença causada pelo SARS-CoV-2 (COVID-19) que será utilizada neste estudo será aquela declarada pelos guias de FDA [26], conforme segue:

Qualquer pessoa que apresente pelo menos um dos seguintes sintomas por dois dias ou mais poderá fazer teste para detecção de ácido nucleico do SARS-CoV-2 em amostra clínica:

- Febre ou calafrios;
- Tosse;
- Falta de ar ou dificuldade para respirar;
- Fadiga;
- Dores muscular ou do corpo;
- Cefaleia;
- Perda de olfato ou paladar nova;
- Dor de garganta;
- Congestão nasal ou coriza;
- Náusea ou vômito;
- Diarreia.

A definição dos sintomas é subjetiva e depende do que cada participante considera anormal para sua condição rotineira. Recomenda-se avaliar todos os participantes entre o segundo e o sétimo dia de apresentação desses sintomas, embora a coleta é permitida até o decimo quarto dia após o início dos sintomas.

Critério de laboratório

- Detecção de ácido nucleico do SARS-CoV-2 em amostra clínica.

Classificação dos casos

Caso possível:

Qualquer pessoa que preencher os critérios clínicos.

Caso confirmado:

Qualquer pessoa que preencher os critérios de laboratório.

Caso descartado:

Refere-se a todos os possíveis casos de COVID-19 que tiveram dois testes para detecção de ácido nucleico do SARS-CoV-2 em amostra clínica negativos com, pelo menos, dois dias de intervalo entre eles. Para a segunda coleta deve ser respeitado o prazo de coleta de amostra para testes para detecção de ácido nucleico do SARS-CoV-2 de 14 dias após o início dos sintomas, sendo preferencialmente até o sétimo dia. Caso a segunda coleta ultrapasse os 14 dias, será avaliada amostra sorológica até o dia 28 após o início dos sintomas para avaliar possível exposição recente.

Caso grave de COVID

Refere-se a um caso confirmado laboratorialmente de infecção por SARS-CoV-2 que apresente uma ou mais das condições a seguir [26]:

- Sinais clínicos em repouso indicando doença sistêmica grave (frequência respiratória ≥ 30 por minuto, frequência cardíaca ≥ 125 por minuto, Saturação de Oxigênio ≤ 93% em temperatura ambiente ao nível do mar ou PaO2/FiO2 < 300 mm Hg);

- Falha respiratória (definida como necessidade de oxigênio suplementar de alto fluxo, ventilação não invasiva, ventilação mecânica ou oxigenação extracorpórea);

- Evidência de choque (PA Sistólica < 90 mm Hg, PA Diastólica < 60 mm Hg, ou necessidade de vasopressores);

- Disfunção importante aguda renal, hepática ou neurológica;

- Admissão em Unidade de Terapia Intensiva;

- Morte.

Escala de progressão clínica de infecção por SARS-CoV-2

Todos os casos de infecção por SARS-CoV2 serão classificados conforme a escala de progressão clínica proposta pela Organização Mundial da Saúde [41] conforme a Tabela 9. A avaliação de casos hospitalizados (score 4 ou superior) será feita diariamente até a resolução dos sintomas. Nos casos não hospitalizados, serão registrados o máximo score e a duração dos sintomas (score 1-3).

Tabela 9. Escala de progressão clínica de infecção por SARS- CoV-2. Adaptada de proposta da Organização Mundial da Saúde[41]

Descrição	**Score**
Não infectado, RNA viral não detectado	**0**
Assintomático, RNA viral detectado	**1**
Sintomático, independente	**2**
Sintomático, precisa de ajuda	**3**
Hospitalizado*, sem precisar de oxigênio	**4**
Hospitalizado, oxigênio suplementar por máscara ou cânula nasal	**5**

Descrição	Score
Hospitalizado, oxigênio por ventilação não invasiva ou de alto fluxo	**6**
Intubação e ventilação mecânica, $P0_2/FiO_2 \geq 150$ ou $SpO_2/FiO_2 \geq 200$	**7**
Ventilação mecânica $P0_2/FiO_2 < 150$ ($SpO_2/FiO_2 < 200$) ou vasopressores	**8**
Ventilação mecânica $P0_2/FiO_2 < 150$ ($SpO_2/FiO_2 < 200$) e vasopressores, dialise ou oxigenação extracorpórea	**9**
Óbito	**10**

* Se a hospitalização for unicamente por isolamento, registre o status com paciente ambulatorial

3.16.8 Resposta a achados novos ou inesperados e alterações no ambiente do estudo

Todos os eventos adversos serão avaliados por um Comitê Independente de Monitoramento de Dados e Segurança. Caso o número de eventos adversos seja superior ao esperado (ver seção 3.15.3 deste protocolo), a administração do produto sob investigação poderá ser suspensa.

O pesquisador principal e/ou o patrocinador poderão suspender a administração do produto sob investigação para proteger os participantes, caso seja identificado um risco potencial não previsto.

No caso de suspensão da administração do produto sob investigação, os participantes continuarão com suas avaliações de segurança acrescentando-se novos parâmetros que sejam necessários prevenção de possíveis riscos. A continuação ou interrupção definitiva da administração do produto sob investigação só acontecerá após aprovação do Comitê de Ética em Pesquisa.

3.17 Garantia/controle de qualidade de dados

3.17.1 Monitoramento clínico

O monitoramento deste estudo será conduzido de acordo com o Documento das Américas para as Boas Práticas Clínicas e procedimentos operacionais padrão em conformidade com as regulamentações governamentais aplicáveis.

O investigador principal será informado sobre a frequência das visitas de monitoramento, notificado com antecedência antes de cada visita e deverá estar disponível, juntamente com o restante da equipe do centro de pesquisa, durante todas as visitas para o esclarecimento de dúvidas e discussões sobre o monitoramento do estudo.

Os objetivos das visitas de monitoramento serão verificar a pronta comunicação de eventos adversos, a existência do TCLE assinado para cada participante, comparar CRFs e documentos fontes para averiguar a sua integralidade e exatidão garantindo a proteção dos participantes do estudo, checar condução do estudo conforme o protocolo, e avaliar a integralidade e exatidão de outros registros que forem considerados necessários. A documentação do estudo estará disponível para análise, quando solicitada, durante todo o curso do estudo.

Além das visitas de monitoramento, os monitores acompanharão a entrada de dados nos CRFs eletrônicos para detectar de forma precoce qualquer anomalia que requeira atenção antes da realização das visitas seguintes. Será dada ênfase especial aos eventos adversos graves e informações que possam comprometer a segurança dos participantes do estudo. Considerando restrições das autoridades de saúde e de biossegurança em áreas onde estejam localizados os centros de pesquisa, o plano de

monitoramento contemplará a possibilidade de monitoramento centralizado e/ou remoto conforme as circunstâncias do centro de pesquisa e a maior eficiência no processo dentro das diretrizes das autoridades éticas e regulatórias.

3.17.2 Acesso aos documentos fonte

Documentos relacionados ao estudo deverão ser mantidos pelo investigador principal de maneira segura por um período de dois anos após a aprovação final da comercialização da vacina do estudo, ou até que dois anos tenham decorrido desde a interrupção formal do desenvolvimento clínico do produto. O tempo de manutenção desses documentos também deve estar em conformidade com o tempo requisitado pelo Comitê de Ética em Pesquisa e pela ANVISA, devendo prevalecer o tempo mais longo. É obrigação do patrocinador informar ao investigador principal, por escrito, o momento em que não houver mais necessidade de manter estes documentos.

Nenhum documento do estudo deverá ser destruído antes do prazo estabelecido por preceitos legais e pelas normas éticas e regulatórias do Brasil sem o acordo prévio por escrito entre o patrocinador, o Comitê de Ética em Pesquisa, Comitê Independente de Monitoramento de Dados e Segurança e a ANVISA.

Nenhum registro médico de instituição externa será solicitado a menos que exista necessidade de esclarecer a história clínica do participante e só será solicitado após o consentimento livre e esclarecido do participante por escrito.

Todos os documentos fonte estarão disponíveis mediante requisição para a avaliação de monitores e auditores nomeados pelo patrocinador e para inspetores de autoridades regulatórias competentes de acordo com as regulamentações e disposições legais vigentes.

4 Ética e publicações

4.1 Aprovações para a realização da pesquisa

4.1.1 Declaração do marco regulatório do estudo

O protocolo está regulado pelas Resoluções 466/12 [42]e 441/2011 [43] do Conselho Nacional de Saúde, a Declaração de Helsinque em sua versão mais recente e as Guias sobre Boas Práticas Clínicas da Conferência Internacional de Harmonização, a RDC 9/2015 da ANVISA [44]. Qualquer outra norma aplicável que venha ser incorporada à normatividade brasileira no decorrer do protocolo será aplicada a este estudo.

4.1.2 Aprovações do estudo

Cada investigador principal conduzirá o estudo em conformidade com o protocolo acordado com o patrocinador e aprovado por seu Comitê de Ética em Pesquisa e pela Agência Nacional de Vigilância Sanitária do Brasil – ANVISA. A versão original do protocolo está em português.

4.2 EMENDAS AO PROTOCOLO

Caso necessário, representantes do patrocinador e investigadores poderão promover emendas no protocolo em comum acordo. As emendas ao protocolo deverão ser encaminhadas para aprovação dos respectivos Comitês de Ética em Pesquisa antes de sua implementação. A ANVISA será notificada de cada emenda assim que seja obtida a aprovação dos Comitês de Ética nos termos da regulação vigente. O estudo está registrado em bases de dados públicas que serão atualizados assim que a emenda obtiver todas as aprovações necessárias.

Nenhum investigador está autorizado a realizar qualquer alteração neste protocolo sem obter autorização do patrocinador e do Comitê de Ética em Pesquisa exceto para eliminar risco(s) imediato(s) aos participantes. Em caso de emergência para eliminar risco dos participantes, a emenda para informar aos Comitês de Ética e a ANVISA será encaminhada com a maior brevidade.

4.3 CONSENTIMENTO LIVRE E ESCLARECIDO

4.3.1 PROCEDIMENTOS PARA A IMPLEMENTAÇÃO E DOCUMENTAÇÃO DO CONSENTIMENTO LIVRE E ESCLARECIDO

Para obter e documentar o consentimento livre e esclarecido, o investigador principal e sua equipe deverão cumprir as normas e requisitos regulatórios aplicáveis, as Boas Práticas Clínicas e os princípios éticos.

O Termo de Consentimento Livre e Esclarecido deverá ser aprovado pelo Comitê de Ética em Pesquisa antes da sua utilização. O participante terá a oportunidade de ler, fazer perguntas e esclarecer suas dúvidas antes de confirmar sua intenção de participar do estudo e receberá uma cópia do TCLE assinado. O participante poderá retirar seu consentimento a qualquer momento, mesmo após o início dos procedimentos do estudo, sem prejuízo para seu atendimento na instituição, caso este atendimento seja decorrente da sua participação no estudo. Os direitos e o bem-estar dos participantes serão protegidos enfatizando-se que a qualidade do seu atendimento médico não será afetada caso ele não aceite participar no estudo.

4.3.2 ARMAZENAMENTO DE AMOSTRAS BIOLÓGICAS E USO DE BIORREPOSITÓRIOS

Todo o material biológico coletado no decorrer do estudo poderá, com a permissão do participante, ser guardado para a sua utilização em pesquisas futuras em biorrepositório específico do estudo. Esse material pode ser utilizado para validação de resultados e aprimoramento dos testes no decorrer do estudo e estabelecer comparações longitudinais dos participantes através do tempo de acompanhamento.

As amostras armazenadas também poderão ser usadas para obter dados para investigação de eventos adversos quando houver indicação médica. O prazo de retenção do material será até a finalização do processamento de todas as amostras do estudo, exceto quando mediar autorização específica para retenção por um período maior para realização de sub-estudos conforme as normas éticas. O participante poderá retirar sua permissão a qualquer momento, sendo, nesse caso, colocado o material a seu dispor.

A ampliação do prazo de armazenamento estará sujeita à aprovação ética conforme às disposições normativas vigentes. Este material biológico não será comercializado ou utilizado para a elaboração de produtos comerciais. Todo o material biológico será armazenado e será identificado com o número de identificação recebido pelo participante após a sua inclusão no estudo. Amostras poderão ser exportadas ao laboratório onde foi desenvolvida a vacina na China para validação de resultados e aprimoramento dos testes. Os pesquisadores brasileiros terão acesso irrestrito às amostras exportadas e serão respeitadas as normas brasileiras sobre pesquisa em seres humanos e armazenamento de amostras, inclusive no concernente à proibição de comercialização ou de depósito de patente de descobertas nessas amostras.

A caracterização extensa da resposta imunológica gerada pela vacina é fundamental para uma compreensão adequada do seu perfil de segurança e imunogenicidade. O armazenamento de amostras de sangue dos participantes incluídos neste estudo irá possibilitar a realização de projetos de pesquisa adicionais que auxiliarão no aprimoramento desta vacina ou no desenvolvimento futuro de outras vacinas de COVID-19.

Qualquer novo projeto de pesquisa que seja realizado no futuro com o material armazenado será submetido para aprovação da Comissão de Ética em Pesquisa. Todos os procedimentos de manejo do biorrepositório seguirão as orientações da Resolução N° 441 do Conselho Nacional de Saúde [43].

4.4 Descrição dos riscos (físicos, sociais e psicológicos) para o indivíduo ou grupo e métodos para minimizar estes riscos

Neste estudo, os riscos para os participantes estão associados à punção venosa e à imunização. As participantes do sexo feminino serão advertidas sobre os riscos desconhecidos da vacina utilizada neste estudo para o feto e serão aconselhadas a utilizar métodos de contracepção eficazes durante a sua participação no estudo, quando necessário.

4.4.1 Detalhamento dos riscos do estudo

4.4.1.1 Punção venosa

Os riscos associados à punção venosa incluem a necessidade ocasional de mais de uma punção durante a realização da coleta, dor e hematoma no local da punção venosa. A ocorrência de desmaio ou infecção no local da punção são raros. Para minimizar esses riscos, a coleta de sangue será realizada por pessoal treinado, experiente, utilizando material individual, descartável e técnica asséptica de coleta.

De acordo com a Portaria n° 1.353 de 14 de Junho de 2011 do Ministério da Saúde, o volume de sangue aceitável para coleta é de nove (9) mL/kg de peso para homens e de oito (8) m/kg de peso para mulheres, sendo que um indivíduo pode realizar uma doação de sangue até três vezes por ano. Portanto, o volume de sangue coletado durante o estudo pode se considerar como seguro para os participantes.

4.4.1.2 Vacinação

Possíveis reações adversas locais incluem dor, inchaço, induração, prurido ou eritema que podem perdurar até dois a três dias após a vacinação, além de inflamação em linfonodos que drenam o local de

administração. Reações sistêmicas como dor, cefaleia e febre de forma similar como acontece com outras vacinas podem acontecer após o uso de vacinas similares às utilizadas neste estudo.

Assim como acontece com qualquer vacina em fase de investigação, existe a possibilidade teórica de riscos sobre os quais não temos conhecimento no momento. Os participantes serão informados caso novas informações se tornem disponíveis ou qualquer novo risco seja identificado. É importante salientar que o estudo em primatas não humanos não resultou em quaisquer problemas de segurança relativos à exposição ao SARS-CoV-2 após vacinação, como por exemplo, de qualquer exacerbação da doença entre os animais vacinados. Desta forma, a possibilidade de uma exacerbação de COVID-19 dependente de anticorpos permanece como sendo apenas um risco teórico das vacinas sem nenhuma comprovação até o momento.

4.4.2 Descrição dos benefícios antecipados ao participante do estudo

Os participantes não receberão qualquer benefício direto pela participação neste estudo. Existe a possibilidade de os participantes desenvolverem imunidade contra o vírus SARS-CoV-2 que seja protetora contra a COVID-19.

Espera-se que as informações obtidas com o presente estudo confirmem a eficácia e segurança da vacina, viabilizando seu registro para uso pela população.

4.4.3 Descrição da relação entre os riscos potenciais e benefícios antecipados

Considerando os resultados de estudos semelhantes que avaliaram vacinas de vírus inativados, não se espera que ocorram Eventos Adversos Graves associados à Vacina do Estudo. Mesmo assim, a equipe do estudo procurará ativamente diminuir os riscos decorrentes das coletas de sangue e detectar precocemente todos os casos de COVID-19 para tomar medidas adequadas de isolamento preventivo.

Atualmente, a COVID-19 encontra-se amplamente distribuída, apresenta uma alta incidência no Brasil e os profissionais de saúde se encontram em risco elevado. Portanto, os esforços para encontrar uma vacina preventiva contra a COVID-19 representam um grande benefício em potencial para esta população.

4.4.4 Descrição de custos e justificativa de reembolsos ou compensações que serão usados

A participação no estudo não terá nenhum custo para os participantes. Os participantes não receberão qualquer compensação em dinheiro para participar deste estudo.

Os custos com o transporte ou estacionamento para participar das visitas programadas serão ressarcidos a cada visita e um lanche leve poderá ser oferecido após cada coleta de sangue.

4.5 Confidencialidade

Todas as informações relacionadas com o estudo serão armazenadas de forma segura no centro de pesquisa, em arquivos trancados com chave com acesso restrito ao pessoal do estudo. Todas as amostras de laboratório, relatórios, formulários administrativos e para coleta de dados serão identificados apenas pelo número de randomização dos participantes com o objetivo de manter a sua confidencialidade.

Informações que permitam a identificação do participante só serão acessíveis, dentro do centro de pesquisa respectivo, aos membros da equipe de pesquisa encarregados do cuidado do participante, aos monitores clínicos e auditores nomeados pelo patrocinador para supervisionar as atividades do centro e aos inspetores das autoridades regulatórias e éticas dentro do marco da legislação vigente.

4.6 Declaração de conflito de interesses

Todos os pesquisadores principais do protocolo assinarão declarações de conflito de interesses antes do início do estudo. O Instituto Butantan, patrocinador da pesquisa e produtor da vacina, é uma instituição pública da Secretaria do Estado da Saúde de São Paulo e não há possibilidade de retorno financeiro para os investigadores ou funcionários do Instituto na forma de royalties, ações ou dividendos pelas vendas da vacina, caso a sua comercialização seja aprovada.

4.7 Acesso aos dados

As bases de dados originais com a informação coletada durante o estudo, sem identificadores dos participantes, serão custodiadas pelo patrocinador.

Os pesquisadores terão livre acesso às informações coletadas em seus respectivos centros no decorrer do estudo. Os investigadores poderão solicitar análises adicionais e/ou acesso ao conjunto de dados de todos os centros desde que mantida a confidencialidade e os procedimentos previstos em protocolo.

Análises adicionais durante a execução do estudo que modifiquem os objetivos e desfecho do presente protocolo podem ser consideradas emendas e deverão ser aprovadas pelo patrocinador e as autoridades éticas e regulatórias correspondentes.

Os dados serão disponibilizados a pedido das autoridades regulatórias dentro do marco legal vigente.

4.8 Seguros e cuidado de emergência

Na ocorrência de eventos adversos associados à participação no estudo, o voluntário receberá assistência médica integral na instituição através da qual ele foi recrutado. O presente estudo está amparado por uma apólice de seguros para cobrir prejuízos aos participantes relacionados com o uso do produto sob investigação nos termos desse protocolo.

4.9 Comunicado aos participantes de seus resultados individuais

Os participantes do estudo receberão seus resultados laboratoriais de testes validados assim que estiverem disponíveis, inclusive de testes realizados para confirmar um caso de suspeita de COVID-19. Os testes experimentais e aqueles para avaliar a resposta de anticorpos à vacinação só ficarão disponíveis após a quebra do cegamento. Se necessário, o participante poderá ser convocado para a entrega dos resultados e esclarecimentos sobre as ações que sejam relevantes para sua saúde de acordo com esses resultados.

4.9.1 Divulgação dos resultados ao público

A divulgação completa do estudo será realizada através de publicações científicas revisadas por pares. A decisão de autoria das publicações seguirá os Requerimentos Uniformes para Encaminhamento de Manuscritos a Revistas Biomédicas do Comitê Internacional de Editores de Revistas Médicas disponível no site http://www.icmje.org.

Um comitê ad hoc com representantes do patrocinador, do fabricante e dos pesquisadores se encarregará de discutir as propostas de divulgação de resultados. Membros desse comitê escreverão o rascunho do manuscrito da publicação e encaminharão para os possíveis coautores para aportes e aprovação. O patrocinador deverá ser consultado com quatro semanas de antecedência do encaminhamento de qualquer resultado derivado deste estudo para divulgação em evento científico ou publicação. O estudo está registrado em base de dados pública desde antes de iniciar o recrutamento e seus resultados serão resumidos nesse mesmo registro: Registro em ClinicalTrials.gov: NCT04456595. Nenhum resultado do estudo poderá ser divulgado na mídia sem que seja apresentado anteriormente em publicação revisada por pares.

4.9.2 COMUNICADO AOS PARTICIPANTES DOS ACHADOS DO ESTUDO

Todos os participantes do estudo receberão um resumo da publicação científica principal revisada por pares e redigido em linguagem apropriada com os principais resultados da pesquisa. Esse resumo estará sujeito à aprovação prévia do Comitê de Ética em Pesquisa da instituição que executa o estudo. Será divulgado ainda um comunicado de imprensa com os principais achados relatados nessa publicação científica.

4.9.3 ACESSO DE TERCEIROS AOS DADOS DO ESTUDO

O patrocinador considerará solicitações justificadas de acesso às bases de dados para análises adicionais por parte de terceiros quando for finalizado o estudo e forem realizadas as análises descritas neste protocolo e no plano de análise de dados do estudo. As solicitações poderão ser aceitas em forma parcial ou total. Para decidir sobre essas solicitações, o patrocinador deverá avaliar a proteção da confidencialidade dos participantes e a qualidade técnica da proposta.

5 REFERÊNCIAS

1. de Wilde AH, Snijder EJ, Kikkert M, van Hemert MJ. Host Factors in Coronavirus Replication. In 2017. p. 1–42. Available from: http://link.springer.com/10.1007/82_2017_25

2. Callow KA, Parry HF, Sergeant M, Tyrrell DAJ. The time course of the immune response to experimental coronavirus infection of man. Epidemiol Infect [Internet]. 1990 Oct 15;105(2):435–46. Available from: https://www.cambridge.org/core/product/identifier/S0950268800048019/type/journal_article

3. Cao W-C, Liu W, Zhang P-H, Zhang F, Richardus JH. Disappearance of Antibodies to SARS-Associated Coronavirus after Recovery. N Engl J Med [Internet]. 2007 Sep 13;357(11):1162–3. Available from: http://www.nejm.org/doi/abs/10.1056/NEJMc070348

4. Lin Q, Zhu L, Ni Z, Meng H, You L. Duration of serum neutralizing antibodies for SARS-CoV-2: Lessons from SARS-CoV infection. J Microbiol Immunol Infect [Internet]. 2020 Mar; Available

from: https://linkinghub.elsevier.com/retrieve/pii/S168411822030075X

5. Payne DC, Iblan I, Rha B, Alqasrawi S, Haddadin A, Al Nsour M, et al. Persistence of Antibodies against Middle East Respiratory Syndrome Coronavirus. Emerg Infect Dis [Internet]. 2016 Oct;22(10):1824–6. Available from: http://wwwnc.cdc.gov/eid/article/22/10/16-0706_article.htm

6. Zhu N, Zhang D, Wang W, Li X, Yang B, Song J, et al. A Novel Coronavirus from Patients with Pneumonia in China, 2019. N Engl J Med [Internet]. 2020 Feb 20;382(8):727–33. Available from: http://www.nejm.org/doi/10.1056/NEJMoa2001017

7. Mousavizadeh L, Ghasemi S. Genotype and phenotype of COVID-19: Their roles in pathogenesis. J Microbiol Immunol Infect [Internet]. 2020 Mar; Available from: https://linkinghub.elsevier.com/retrieve/pii/S1684118220300827

8. Andersen KG, Rambaut A, Lipkin WI, Holmes EC, Garry RF. The proximal origin of SARS-CoV-2. Nat Med [Internet]. 2020 Apr 17;26(4):450–2. Available from: http://www.nature.com/articles/s41591-020-0820-9

9. Forster P, Forster L, Renfrew C, Forster M. Phylogenetic network analysis of SARS-CoV-2 genomes. Proc Natl Acad Sci [Internet]. 2020 Apr 28;117(17):9241–3. Available from: http://www.pnas.org/lookup/doi/10.1073/pnas.2004999117

10. van Doremalen N, Bushmaker T, Morris DH, Holbrook MG, Gamble A, Williamson BN, et al. Aerosol and Surface Stability of SARS-CoV-2 as Compared with SARS-CoV-1. N Engl J Med [Internet]. 2020 Apr 16;382(16):1564–7. Available from: http://www.nejm.org/doi/10.1056/NEJMc2004973

11. Huang C, Wang Y, Li X, Ren L, Zhao J, Hu Y, et al. Clinical features of patients infected with 2019 novel coronavirus in Wuhan, China. Lancet [Internet]. 2020 Feb;395(10223):497–506. Available from: https://linkinghub.elsevier.com/retrieve/pii/S0140673620301835

12. Chow N, Fleming-Dutra K, Gierke R, Hall A, Hughes M, Pilishvili T, et al. Preliminary Estimates of the Prevalence of Selected Underlying Health Conditions Among Patients with Coronavirus Disease 2019 — United States, February 12–March 28, 2020. MMWR Morb Mortal Wkly Rep [Internet]. 2020 Apr 3;69(13):382–6. Available from: http://www.cdc.gov/mmwr/volumes/69/wr/mm6913e2.htm?s_cid=mm6913e2_w

13. Zhu J, Ji P, Pang J, Zhong Z, Li H, He C, et al. Clinical characteristics of 3,062 COVID-19 patients: a meta-analysis. J Med Virol [Internet]. 2020 Apr 15; Available from: http://doi.wiley.com/10.1002/jmv.25884

14. Lechien JR, Chiesa-Estomba CM, De Siati DR, Horoi M, Le Bon SD, Rodriguez A, et al. Olfactory and gustatory dysfunctions as a clinical presentation of mild-to-moderate forms of the coronavirus disease (COVID-19): a multicenter European study. Eur Arch Oto-Rhino-Laryngology [Internet]. 2020 Apr 6; Available from: http://link.springer.com/10.1007/s00405-020-05965-1

15. Bialek S, Gierke R, Hughes M, McNamara LA, Pilishvili T, Skoff T. Coronavirus Disease 2019 in Children — United States, February 12–April 2, 2020. MMWR Morb Mortal Wkly Rep [Internet]. 2020 Apr 10;69(14):422–6. Available from: http://www.cdc.gov/mmwr/volumes/69/wr/mm6914e4.htm?s_cid=mm6914e4_w

16. Dong Y, Mo X, Hu Y, Qi X, Jiang F, Jiang Z, et al. Epidemiology of COVID-19 Among Children in China. Pediatrics [Internet]. 2020 Mar 16;e20200702. Available from: http://pediatrics.aappublications.org/lookup/doi/10.1542/peds.2020-0702

17. Huang L, Zhang X, Zhang X, Wei Z, Zhang L, Xu J, et al. Rapid asymptomatic transmission of COVID-19 during the incubation period demonstrating strong infectivity in a cluster of youngsters aged 16-23 years outside Wuhan and characteristics of young patients with COVID-19: A prospective contact-tracing study. J Infect [Internet]. 2020 Jun;80(6):e1–13. Available from: https://linkinghub.elsevier.com/retrieve/pii/S0163445320301171

18. Leal FE, Mendes-Correa MC, Buss LF, Costa SF, Bizario JCS, Souza SRP, et al. A primary care approach to the COVID-19 pandemic: clinical features and natural history of 2,073 suspected cases in the Corona Sao Caetano programme, Sao Paulo, Brazil. medRxiv [Internet]. 2020 Jan 1;2020.06.23.20138081. Available from: http://medrxiv.org/content/early/2020/06/23/2020.06.23.20138081.abstract

19. Verity R, Okell LC, Dorigatti I, Winskill P, Whittaker C, Imai N, et al. Estimates of the severity of coronavirus disease 2019: a model-based analysis. Lancet Infect Dis [Internet]. 2020 Mar; Available from: https://linkinghub.elsevier.com/retrieve/pii/S1473309920302437

20. Long Q-X, Tang X-J, Shi Q-L, Li Q, Deng H-J, Yuan J, et al. Clinical and immunological assessment of asymptomatic SARS-CoV-2 infections. Nat Med [Internet]. 2020 Jun 18; Available from: http://www.nature.com/articles/s41591-020-0965-6

21. Lin L, Lu L, Cao W, Li T. Hypothesis for potential pathogenesis of SARS-CoV-2 infection–a review of immune changes in patients with viral pneumonia. Emerg Microbes Infect [Internet]. 2020 Jan 1;9(1):727–32. Available from: https://www.tandfonline.com/doi/full/10.1080/22221751.2020.1746199

22. Lin J-T, Zhang J-S, Su N, Xu J-G, Wang N, Chen J-T, et al. Safety and immunogenicity from a phase I trial of inactivated severe acute respiratory syndrome coronavirus vaccine. Antivir Ther [Internet]. 2007;12(7):1107–13. Available from: http://www.ncbi.nlm.nih.gov/pubmed/18018769

23. Folegatti PM, Bittaye M, Flaxman A, Lopez FR, Bellamy D, Kupke A, et al. Safety and immunogenicity of a candidate Middle East respiratory syndrome coronavirus viral-vectored vaccine: a dose-escalation, open-label, non-randomised, uncontrolled, phase 1 trial. Lancet Infect Dis [Internet]. 2020 Apr 20; Available from: http://www.ncbi.nlm.nih.gov/pubmed/32325038

24. Thanh Le T, Andreadakis Z, Kumar A, Gómez Román R, Tollefsen S, Saville M, et al. The COVID-19 vaccine development landscape. Nat Rev Drug Discov [Internet]. 2020 May 9;19(5):305–6. Available from: http://www.nature.com/articles/d41573-020-00073-5

25. Lambert P-H, Ambrosino DM, Andersen SR, Baric RS, Black SB, Chen RT, et al. Consensus summary report for CEPI/BC March 12–13, 2020 meeting: Assessment of risk of disease enhancement with COVID-19 vaccines. Vaccine [Internet]. 2020 Jun;38(31):4783–91. Available from: https://linkinghub.elsevier.com/retrieve/pii/S0264410X2030709X

26. Development and Licensure of Vaccines to Prevent COVID-19: Guidance for Industry [Internet]. Silver Spring; 2020. Available from: www.fda.gov/media/139638/download

27. Hotez PJ, Corry DB, Strych U, Bottazzi ME. COVID-19 vaccines: neutralizing antibodies and the alum advantage. Nat Rev Immunol [Internet]. 2020 Jul 4;20(7):399–400. Available from: http://www.nature.com/articles/s41577-020-0358-6

28. Hotez PJ, Bottazzi ME, Corry DB. The potential role of Th17 immune responses in coronavirus immunopathology and vaccine-induced immune enhancement. Microbes Infect [Internet]. 2020 May;22(4–5):165–7. Available from: https://linkinghub.elsevier.com/retrieve/pii/S1286457920300721

29. Bottazzi ME, Strych U, Hotez PJ, Corry DB. Coronavirus Vaccine-Associated Lung Immunopathology-What Is The Significance? Microbes Infect [Internet]. 2020 Jun; Available from: https://linkinghub.elsevier.com/retrieve/pii/S1286457920301258

30. Gao Q, Bao L, Mao H, Wang L, Xu K, Yang M, et al. Development of an inactivated vaccine candidate for SARS-CoV-2. Science (80-) [Internet]. 2020 May 6;eabc1932. Available from: https://www.sciencemag.org/lookup/doi/10.1126/science.abc1932

31. Zhang Y-J, Zeng G, Pan H-X, Li C-G, Kan B, Hu Y-L, et al. Immunogenicity and Safety of a SARS-CoV-2 Inactivated Vaccine in Healthy Adults Aged 18-59 years: Report of the Randomized, Double-blind, and Placebo-controlled Phase 2 Clinical Trial. medRxiv [Internet]. 2020; Available from: https://www.medrxiv.org/content/early/2020/08/10/2020.07.31.20161216

32. Grifoni A, Weiskopf D, Ramirez SI, Mateus J, Dan JM, Moderbacher CR, et al. Targets of T Cell Responses to SARS-CoV-2 Coronavirus in Humans with COVID-19 Disease and Unexposed Individuals. Cell [Internet]. 2020 Jun;181(7):1489-1501.e15. Available from: https://linkinghub.elsevier.com/retrieve/pii/S0092867420306103

33. Curtis KM, Tepper NK, Jatlaoui TC, Berry-Bibee E, Horton LG, Zapata LB, et al. U.S. Medical Eligibility Criteria for Contraceptive Use, 2016. MMWR Recomm Reports [Internet]. 2016 Jul 29;65(3):1–103. Available from: http://www.cdc.gov/mmwr/volumes/65/rr/rr6503a1.htm

34. Rüggeberg JU, Gold MS, Bayas J-M, Blum MD, Bonhoeffer J, Friedlander S, et al. Anaphylaxis: Case definition and guidelines for data collection, analysis, and presentation of immunization safety data. Vaccine [Internet]. 2007 Aug;25(31):5675–84. Available from: https://linkinghub.elsevier.com/retrieve/pii/S0264410X07002642

35. Hanley JA. If Nothing Goes Wrong, Is Everything All Right? JAMA [Internet]. 1983 Apr 1;249(13):1743. Available from: http://jama.jamanetwork.com/article.aspx?doi=10.1001/jama.1983.03330370053031

36. Brasil M da S. Diretrizes Operacionais para o Estabelecimento e o Funcionamento de Comitês de Monitoramento de Dados e de Segurança. 2008.

37. ICH. Clinical Safety Data management: Definitions and Standards for Expedited Reporting [Internet]. Genebra: International Conference on Harmonisation of Technical Requirements ror Registration of Pharmaceuticals for Human Use; 1994 [citado 2011 Set 29]. E2A .

38. Agência Nacional de Vigilância Sanitária. Gerência de Farmacovigilância, Ministério da Saúde B. Guias de farmacovigilância para detentores de registro de Medicamentos. Ministério da Saúde Bras{\$\$’\$\$i}lia; 2010.

39. UMC/WHO. The use of the WHO-UMC system for standardised case causality assessment [Internet]. Uppsala:The Uppsala Monitoring Centre. [citado 2011 Set 29]. Disponível em: http://www.who-umc.org/Graphics/24734.pdf.

40. Law B, Sturkenboom M. D2.3 Priority List of Adverse Events of Special Interest: COVID-19 [Internet]. Decatur, GA; 2020. Available from: brightoncollaboration.us/wp-content/uploads/2020/06/SPEAC_D2.3_V2.0_COVID-19_20200525_public.pdf

41. Marshall JC, Murthy S, Diaz J, Adhikari NK, Angus DC, Arabi YM, et al. A minimal common outcome measure set for COVID-19 clinical research. Lancet Infect Dis [Internet]. 2020 Aug;20(8):e192–7. Available from: https://linkinghub.elsevier.com/retrieve/pii/S1473309920304837

42. Brasil M da S, de Saúde CN. Resolução nº466, de 12 de dezembro de 2012. Diretrizes e normas

regulamentadoras de pesquisas envolvendo seres humanos. Diário Of da União da República Fed do Bras. 2013;150(112).

43. CNS/MS. Resolução CNS Nº 441 [Internet]. Brasília: Conselho Nacional de Saúde; 2011 [citado 2011 Set 29]. Disponível em: http://conselho.saude.gov.br/resolucoes/2011/Reso441.pdf.

44. Ministério da Saúde B, ANVISA. RDC 09/2015.In: Agência Nacional de Vigilância Sanitária editor. Brasília DF. 114. 2011.

Clinical Research Protocol	COV-02-IB<br><br>Phase III double-blind, randomized, placebo-controlled clinical trial for the evaluation of efficacy and safety in health professionals of the adsorbed vaccine COVID-19 (inactivated) produced by Sinovac
Study acronym	PROFISCOV

English Version: 2.0, 24 August 2020

CONFIDENTIAL

The confidential information contained in this document is provided to you as an investigator or member of a study research team or reviewer within the study approval process. Your acceptance of this document is characterized by an agreement in which you undertake not to disclose the information contained herein to other parties without the written authorization of the Instituto Butantan or its authorized representatives.

Instituto Butantan
Av. Vital Brasil, 1500, Butantã
São Paulo – SP 05503-900 – Brasil
Phone: (11) 3723 2121 – Fax: (11) 2627 9375

CONTENTS

1 ADMINISTRATIVE INFORMATION 8
1.1 Title of Protocol 8
1.2 Registration information 8
1.3 Version history 11
1.4 Financing 11
1.5 Study responsibilities 11
1.5.1 Contributions to the Protocol 11
1.5.2 Role of the sponsor and funders 12
1.5.3 Study committees 12

2 INTRODUCTION 12
2.1 Literature review / Current state of knowledge on COVID-19 12
2.1.1 Coronavirus infections in humans 12
2.1.2 The emergence of SARS-CoV-2 14
2.1.3 Clinical presentations 15
2.1.4 Epidemiology of COVID-19 16
2.1.5 Immune response to the SARS-CoV-2 virus 17
2.1.6 Development of vaccines against COVID-19 18
2.1.7 Development of the adsorbed vaccine COVID-19 (inactivated) produced by Sinovac (CoronaVac) 18
2.2 Objectives 23
2.2.1 Primary objectives 23
2.2.2 Secondary objectives 23
2.2.3 Exploratory objectives 24
2.3 General study design 25

3 METHODS 26
3.1 Study centers 26
3.2 Participants 26
3.2.1 Inclusion criteria 26
3.2.2 Exclusion criteria 26
3.3 Endpoints 28
3.3.1 Primary endpoints 28
3.3.2 Secondary endpoints 28
3.3.3 Exploratory endpoints 29
3.4 Interventions **30**
3.4.1 Final composition and formulations of the adsorbed vaccine covid-19 (inactivated) produced by Sinovac 30
3.4.2 Permitted, not recommended and prohibited concurrent treatment interventions or regimes 31
3.4.3 Risk of pregnancy for female participants 32
3.4.4 Procedures to verify adherence to treatment 33
3.5 Procedures for the participant 33
3.5.1 Monitoring the participants 33
3.5.2 Study procedures 33
3.5.3 Description of the study procedures per visit 33
3.5.4 Vaccination 36
3.5.5 Monitoring of adverse events and use of the “participant's diary” 36
3.5.6 Procedures in case of fever and suspected COVID-19 37

3.5.7 Procedure in case of loss of visit or contact 38
3.6 Diagram of study procedures 38
3.7 Criteria and procedures for suspension or exclusion of participants 39
3.8 Sample size 40
3.8.1 Calculation of sample size and statistical power 40
3.8.2 Estimated number of participants 42
3.9 Study population and recruitment area 42
3.10 Allocation of participants 43
3.10.1 Generation of allocation sequence 43
3.10.2 Allocation concealment mechanism 43
3.10.3 Implementation of the allocation sequence generation 43
3.11 Blinding procedures 44
3.11.1 Description of who will be blind in the study 44
3.11.2 Procedures for breaking the blind 44
3.12 Data collection 44
3.13 Data processing and analysis 45
3.14 Data analysis plan, including statistical methodology 45
3.14.1 Definitions of populations to be analyzed 45
3.14.2 Descriptive methodology 46
3.14.3 Primary efficacy analysis 47
3.14.4 Safety analysis 47
3.14.5 Immunogenicity analysis 48
3.14.6 Management of missing data 49
3.15 Monitoring study data 49
3.15.1 Safety and Data Monitoring Committee 49
3.15.2 Interim analysis 49
3.15.3 Criteria for suspension or interruption of the study 50
3.16 Safety assessment 51
3.16.1 Definitions 51
3.16.2 Monitoring of adverse events 52
3.16.3 Classification of severity 52
3.16.4 Classification of the causal relationship 56
3.16.5 Identification and reporting of adverse events 57
3.16.6 Safety assessment period 59
3.16.7 COVID-19 case definition 60
3.16.8 Response to new or unexpected findings and changes in the study environment 62
3.17 Data quality assurance / control 62
3.17.1 Clinical monitoring 62
3.17.2 Access to source documents 63

4 ETHICS AND PUBLICATIONS 63
4.1 Approvals for conducting the research 63
4.1.1 Statement of the study's regulatory framework 63
4.1.2 Study approvals 63
4.2 Amendments to the protocol 63
4.3 Voluntary informed consent 64
4.3.1 Procedures for implementing and documenting free and informed consent 64
4.3.2 Storage of biological samples and use of biorepositories 64
4.4 Description of risks (physical, social and psychological) for the individual or group and methods to minimize these risks 65
4.4.1 Detailing the risks of the study 65
4.4.2 Description of anticipated benefits for the study participant 66

4.4.3 Description of the relationship between potential risks and anticipated benefits 66
4.4.4 Description of costs and justification for refunds or compensation 66
4.5 Confidentiality 66
4.6 Declaration of conflict of interest 67
4.7 Access to data 67
4.8 Insurance and emergency care 67
4.9 Communicating to participants about their individual results 67
4.9.1 Disclosure of results to the public 67
4.9.2 Communicated to participants of study findings 68
4.9.3 Third party access to study data 68
5 REFERENCES 68

Tables Index

Table 1 Estimates of mortality and hospitalization rates by age group among SARS-CoV-2 infected cases in China [19] 17

Table 2. Incidence of adverse reactions in the phase II study of the adsorbed vaccine COVID-19 (inactivated) [31] 20

Table 3. Seroconversion rates for neutralizing antibodies and IgG ELISA [31] 21

Table 4 Diagram of study procedures 39

Table 5 Classification of severity of solicited local clinical adverse events 53

Table 6 Classification of the severity of the solicited systemic clinical adverse events and of the signs and symptoms in the case of fever and suspected COVID-19 53

Table 7 Classification of the severity of unsolicited clinical Adverse Events and other signs and symptoms in the case of fever and suspected COVID-19 55

Table 8 Classification of the causal relationship of Adverse Events with the investigational product 57

Table 9. Scale of clinical progression of SARS-CoV-2 infection. Adapted from a WHO proposal [41] 61

Figures Index

Figure 1 36-month follow-up of SARS-CoV-1 antibody titers [4] 13

Figure 2 Schematic representation of the SARS-CoV-2 genome (A) and phylogeny (B) [8] 14

Figure 3 Distribution of PCR and serology results by time interval between symptom onset and sample collection in 496 cases of COVID-19 in a community surveillance program [18] 16

1 ADMINISTRATIVE INFORMATION

1.1 TITLE OF THE PROTOCOL

COV-02-IB - Phase III double-blind, randomized, placebo-controlled clinical trial for the evaluation of efficacy and safety in health professionals of the adsorbed vaccine COVID-19 (inactivated) produced by Sinovac - PROFISCOV

1.2 REGISTRATION INFORMATION

Category	***Information***
Primary Record	ClinicalTrials.gov NCT04456595
Registration Date	02-Jul-2020
Sources of financial or material support	Fundação Butantan, Instituto Butantan
Primary sponsor	Instituto Butantan
Contact for public and scientific issues	Ricardo Palacios, MD, PhD<br>Medical Director of Clinical Research<br>Instituto Butantan<br>Av. Vital Brasil, 1500<br>São Paulo, SP, Brazil CEP 05503-900<br>Phone: (11) 37232121<br>E-mail: ricardo.palacios@butantan.gov.br
Scientific title	Phase III double-blind, randomized, placebo-controlled clinical trial for the evaluation of efficacy and safety in health professionals of the adsorbed vaccine COVID-19 (inactivated) produced by Sinovac
Public title	Phase III Clinical Trial for Evaluation of Efficacy and Safety in Health Professionals of the Inactivated COVID-19 Vaccine Produced by Sinovac
Study code	COV-02-IB
Study Acronym	PROFISCOV
Recruitment countries	Brazil
Health conditions or problems	COVID-19
Interventions	
Name	Vaccine (CoronaVac) Adsorbed COVID-19 (inactivated)
Description	Dose 600 SU / dose<br>Via: Intramuscular (deltoid)
Name	Placebo

Inclusion criteria

- Adults over 18 years of age or older;
- Healthcare professionals who work in direct contact care of people with possible or confirmed COVID-19 cases;
- Demonstrate intention to participate in the study, documented by the participant's signing the Voluntary Informed Consent Form.

Exclusion criteria

- For females: pregnancy (confirmed by positive β-hCG test), breastfeeding and / or expressing intention to have sexual practices with reproductive potential without using contraceptive methods during the three months following vaccination;
- Evidence of uncontrolled neurological, cardiac, pulmonary, hepatic or renal disease, according to anamnesis or physical examination. Significant changes in treatment or hospitalizations due to worsening of the condition in the last three months are indicators of uncontrolled disease;
- Diseases with impaired immune system including: neoplasms (except basal cell carcinoma), congenital or acquired immunodeficiencies and uncontrolled autoimmune diseases according to anamnesis or physical examination. Significant changes in treatment or hospitalizations due to worsening of the condition in the last three months are indicators of uncontrolled disease.
- Behavioral, cognitive or psychiatric illness that, in the opinion of the principal investigator or his medical representative, affects the participant's ability to understand and collaborate with the requirements of the study protocol;
- Alcohol or drug abuse in the last 12 months prior to inclusion in the study that has caused medical, professional or family problems, as indicated by clinical history;
- History of severe allergic reaction or anaphylaxis to the vaccine or components of the study vaccine;
- History of asplenia;
- Participation in another clinical trial with product administration under investigation during the six months prior to its inclusion in the study or scheduled participation in another clinical trial in the two years following inclusion;
- Previous participation in a COVID-19 vaccine evaluation study or previous exposure to a COVID-19 vaccine;
- Use of immunosuppressive therapies six months prior to inclusion in the study or scheduled to be used within two years of inclusion. Immunosuppressive therapies considered include antineoplastic chemotherapy, radiation therapy, immunosuppressants to induce tolerance to transplants, among others.
- Have received an immunosuppressive dose of corticosteroids in the last three months prior to inclusion in the study or scheduled administration of an immunosuppressive dose of corticosteroids for the three months following inclusion in the study. The dose of corticosteroids considered immunosuppressive is equivalent to prednisone at a dose of 20 mg / day for adults, for more than a week. The continuous use of topical or nasal corticosteroids is not considered immunosuppressive;
- Have received blood products (transfusions or immunoglobulins) in the last three months before inclusion in the study, or scheduled administration of blood products or immunoglobulin in the two years following inclusion in the study;
- Suspected or confirmed fever within 72 hours prior to vaccination or axillary temperature greater than 37.8 ° C * on the day of vaccination (inclusion may be postponed until the participant completes 72 hours without fever);
- Possible or confirmed case of COVID-19 on the day of vaccination (vaccination can be postponed until the participant completes 72 hours without symptoms or a diagnosis is ruled out);

- Have received vaccination with live attenuated virus in the last 28 days or inactivated vaccine in the last 14 days prior to inclusion in the study, or have immunization scheduled for the first 28 days after inclusion in the study;
- History of bleeding disorders (for example, deficiency of clotting factors, coagulopathy, platelet dysfunction), or previous history of bleeding or significant bruising after IM injection or venipuncture;
- Any other condition that, in the opinion of the principal investigator or his medical representative, could jeopardize the safety or rights of a potential participant or that would prevent him from complying with this protocol.

**The temperature measured with a skin thermometer using a temporal scanner is considered equivalent to the axillary temperature.*

Study Type: Phase III clinical trial, randomized, multicenter, double blind and placebo controlled to evaluate the efficacy and safety of the adsorbed vaccine COVID-19 (inactivated) produced by Sinovac.

Allocation type: Randomized controlled clinical study 1: 1
Recruitment status: Ongoing
Date of 1st recruitment: 21 July. 2020
Target sample size: 13,060 participants

Primary endpoints:

- **Efficacy:** The primary efficacy endpoint is the incidence of symptomatic cases of virologically confirmed COVID-19 two weeks after the second vaccination. The virological diagnosis will be confirmed by detection of SARS CoV-2 nucleic acid in clinical samples.
- **Safety:** The primary safety endpoint is the frequency of solicited and unsolicited local and systemic adverse reactions during the period of one week after vaccination stratified by age group (adults 18-59 years and elders 60 years of age or more). Adverse reactions are defined as adverse events that have a reasonable causal relationship to vaccination.

Secondary endpoint:

Efficacy:

1. Incidence of cases of COVID-19 confirmed virologically after administration of the adsorbed vaccine COVID-19 (inactivated) produced by Sinovac, two weeks after the first vaccination.
2. Incidence of symptomatic and asymptomatic SARS-CoV-2 infections detected serologically and / or virologically, two weeks after the second vaccination. Serological confirmation of SARS-CoV-2 infections will be by a four-fold increase in the level of IgG titers in validated serological assays.
3. Incidence of severe cases of COVID-19 confirmed virologically two weeks after the administration of the second dose of the adsorbed vaccine COVID-19 (inactivated).

Safety:

4. Frequency of adverse reactions to the vaccine, local and systemic, solicited and unsolicited, after each of the two doses of the vaccination schedule within the period of four weeks after vaccination stratified by age groups (adults 18- 59 years old, and elderly participants aged 60 years or older).

5. Frequency of severe COVID-19 cases in participants who received at least one dose of the experimental product.
6. Frequency of Adverse Events of Special Interest (AESI) in participants who received at least one dose of the experimental product.

Immunogenicity:

7. Seroconversion rates in the second week after each vaccination according to age group (adults 18-59 years old and elderly participants 60 years of age or older).
8. The cell-mediated immune response in a subgroup of participants before each vaccination and two and four weeks after the second vaccination according to age group (Adults 18-59 years and Elders 60 years of age or older).
9. The frequency of detection of antibodies against SARS-CoV-2 before and two weeks after the administration of the second dose of the adsorbed vaccine COVID-19 (inactivated) produced by Sinovac in adult (18-59 years of age) and elderly (60 years of age or older) participants. The evaluation will be carried out on all research participants using validated ELISA and / or Chemiluminescence methodology.

1.3 VERSION HISTORY

Version 1.0 Date 30-Jun-2020
Initial version submitted to the regulatory agency

Version 1.1 Date 3-Jul-2020
Version incorporating regulatory agency considerations

Version 2.0 Date 24-Aug-2020
Design change and inclusion of participants independently of previous SARS-CoV-2 exposure

English Version 2.0
Date 24-Aug-2020
Translation of Version 2.0

1.4 FINANCING

This study is funded by the Fundação Butantan.

1.5 RESPONSIBILITIES FOR THE PROTOCOL

The names and contact information of those responsible for the protocol are detailed in Annex A.

1.5.1 CONTRIBUTIONS TO THE PROTOCOL

This research protocol was written by the Instituto Butantan team composed of Ricardo Palacios, Elizabeth González Patiño, Roberta de Oliveira Piorelli, Monica Tili Reis Pessoa Conde and Ana Paula Batista. Drs Gang Zeng and Qianqian Xin, from Sinovac, and Dr Esper Kallas, from the Faculty of Medicine of the University of São Paulo, also contributed to the protocol. The principal investigators at each center were also consulted on the content of

the protocol. Jorge Flores, Christopher Gast and Christopher F. Ockenhouse from PATH in the United States participated in the review of the protocol, in particular the study design.

1.5.2 ROLE OF SPONSOR AND FINANCERS

Funding entities will have no influence on the design and execution, analysis, interpretation of data or publication of the results of the study.

The Instituto Butantan, linked to the Secretary of State for Health of São Paulo, assumes the responsibilities of sponsoring the study in accordance with Good Clinical Practices. The sponsor will manage the financial resources to ensure the adequate performance of the study and the data entry platform. The sponsor will also obtain the necessary approvals from the National Health Surveillance Agency. Sinovac will supply the product under investigation.

Data analysis will be carried out by a team from the Instituto Butantan. Researchers will have access to the original databases and will be able to request or perform additional complementary analyses according to a statistical analysis plan to be finalized before freezing the database.

The publication of the results of the study will be made by mutual agreement between the sponsor, Sinovac and the researchers, without prejudice to the researchers' right to present the study and its conclusions in journals and scientific meetings.

1.5.3 STUDY COMMITTEES

The study team by the sponsor will be composed of the sponsor's representative, the sponsor's medical specialists and the study monitoring coordination. This team will be in charge of administering the study and the sponsor-related responsibilities. The sponsor's medical experts will conduct a medical review of adverse events by the sponsor.

An Independent Data and Safety Monitoring Committee will be constituted for the study as described in Section 3.15.1.

2 INTRODUCTION

2.1 LITERATURE REVIEW / CURRENT STATUS OF KNOWLEDGE

2.1.1 CORONAVIRUS INFECTIONS IN HUMANS

Coronaviruses are RNA viruses with a relatively large genome, 30kB, ubiquitously present in nature in several animal species, causing diseases in domestic and industrial animals. Before the appearance of COVID-19, six coronaviruses capable of causing infection in humans were identified. In the 1960s, two coronaviruses causing common cold were identified, HCoV-OC43 and HCoV-229E. In the present century, two more coronaviruses were added as agents associated with the common cold, HCoV-NL63 and HCoV-HKU1. All of them, except the last one, have described genetic proximity relationships with bat coronaviruses, that may have to do with their origin. The two remaining coronaviruses, also identified in the present century were SARS-CoV-1, associated with outbreaks of Severe Acute Respiratory Syndrome

that started in 2002, and the MERS-CoV discovered in 2012, also associated with severe illness, in people in contact with camels and health workers from places that treated these patients [1].

The human immune response to coronaviruses appears to be heterogeneous and transient. In a human controlled infection model of HCoV-229E, the infection was successful in 10 participants with lower IgG and IgA neutralizing antibody titers, but not in the other 5 participants with preexisting immunity. After experimental infection, these titers increased [2]. In the case of SARS-CoV-1, a drop in levels of both IgA and neutralizing antibodies of the IgG type was demonstrated over a 36-month follow-up period (Figure 1), although the clinical significance of this drop is not known, as there is no evidence of reinfection. Furthermore, it was found that one of the sequelae of the clinical picture, femoral necrosis, was associated with lower antibody titers [3]. The drop in antibodies may be even greater in patients with convalescent SARS-CoV-1 four and six years later, with loss of neutralizing antibody response, which remains present in only 50% and 8.7%, of individuals, respectively [4]. A drop in neutralizing antibodies has also been described over time in a limited number of MERS-CoV patients. Likewise, its clinical significance is also unknown and it was not possible to document re-exposure in those cases either [5]. The noted heterogeneity of the coronavirus immune response in humans could also occur in SARS-CoV-2 and would be relevant to the potential development of immunobiologicals.

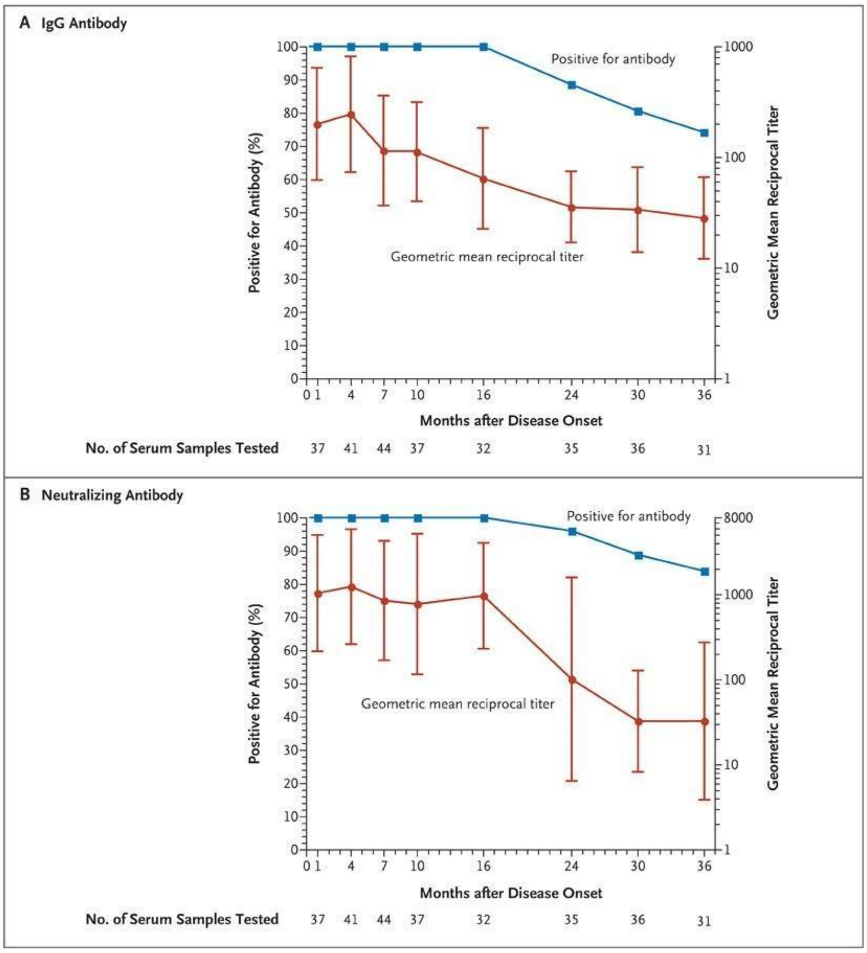

FIGURE 1 FOLLOW-UP OF SARS-COV-1 ANTIBODY TITERS OVER 36 MONTHS *[4]*

2.1.2 THE EMERGENCY OF SARS-CoV-2

At the end of December 2019, an aggregate of pneumonia patients was identified in association with a market in the city of Wuhan, in Hubei Province, China. From samples collected from bronchoalveolar lavage from three of these patients, a coronavirus different from those known to infect humans was identified. The new coronavirus was identified as a beta coronavirus, as well as SARS-CoV-1, with which it has more genetic proximity (Figure 2) [6]. This virus would later be called SARS-CoV-2

SARS-CoV-2 is a spherical or pleomorphic virus composed of a single-stranded RNA associated with a nucleoprotein inside a protein capsule covered by an envelope with spiked glycoproteins, or Spike in English. This Spike protein is fundamental for its replication, in a similar way to SARS-CoV-1, and the neutralizing antibodies are mainly directed against it [7].

The origin of SARS-CoV-2 is zoonotic with genetic proximity to betacoronavirus from bats, including RaTG13, found in the intermediate horseshoe bat (Rhinolophus affinis), the closest genetically. But the most relevant feature of this SARS-CoV-2 is the Angiotensin-Converting Enzyme Receptor 2 (ACE2) Binding Domain in the Spike glycoprotein that allows the virus to enter cells in various human tissues. This domain is very similar to that found in coronaviruses that infect the Malaysian pangolin (Manis javanica) [8]. The mutations so far appear to be limited, although some researchers propose that they would have a differentiation into three types (A, B and C) with a point change in amino acids [9].

The transmission of SARS-CoV-2 infection is mainly by droplets and fomites like many other respiratory viruses and can remain viable for several hours and even days on different surfaces [10]. Replication in the upper airways facilitates person-to-person transmission and contamination of surfaces.

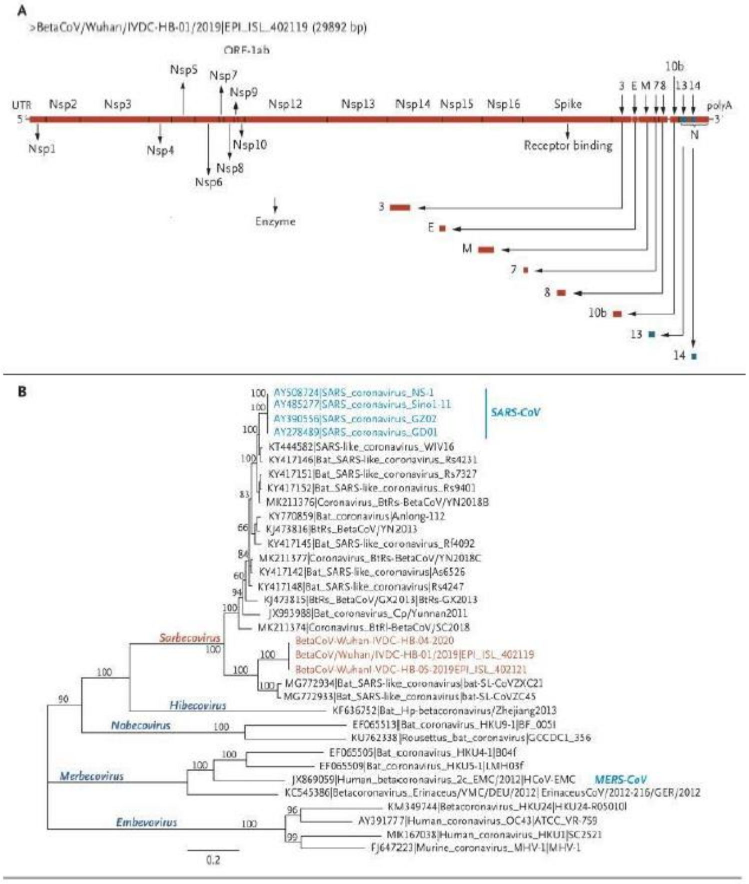

FIGURE 2 SCHEMATIC REPRESENTATION OF THE SARS-COV-2 GENOME (A) AND PHYLOGENY (B) *[8]*

2.1.3 CLINICAL PRESENTATION

Since the initial reports of cases of the disease in the city of Wuhan, the most severe picture of the clinical spectrum of COVID-19 has been consistent and well characterized. The presentation is that of a febrile syndrome with associated respiratory symptoms such as cough, which develops into bilateral pneumonia with diffuse impairment which can progress to acute respiratory distress syndrome, cardiac injury and multiple organ failure. This severe clinical evolution was more frequent in older patients and people with underlying medical conditions such as hypertension, diabetes and cardiovascular disease, among others [11]. The association with underlying medical conditions has been confirmed in other case series [12].

COVID-19 cases present predominantly with fever and may be accompanied by fatigue, non-productive cough or with sputum. Other symptoms include myalgia, anorexia, chest tightness and dyspnea. Less frequent symptoms are nausea and vomiting, diarrhea, headache, pain in the pharynx, chills and abdominal pain [13]. Among the mild and moderate symptoms, it is worth mentioning the changes in smell and taste, which were more common among women [14]. In children, symptoms of COVID-19 appear to be milder and less frequent, including fever and cough compared to adults [15]. The clinical course in children also appears to be more benign with few patients progressing to severe forms [16]. Routine identification of asymptomatic cases is not currently performed, although there are some reports mainly associated with the epidemiological investigation of index case contacts, demonstrating the occurrence of transmission efficiently even in young people [17].

In a recent COVID-19 community surveillance study, the median days among those who had virological confirmation by RT-PCR was 5 days (P25-75 4 -7 days), with an increase in the number of cycles for detecting the virus, which indicates a decrease in viral load in the sample, as the duration of symptoms increased [18].

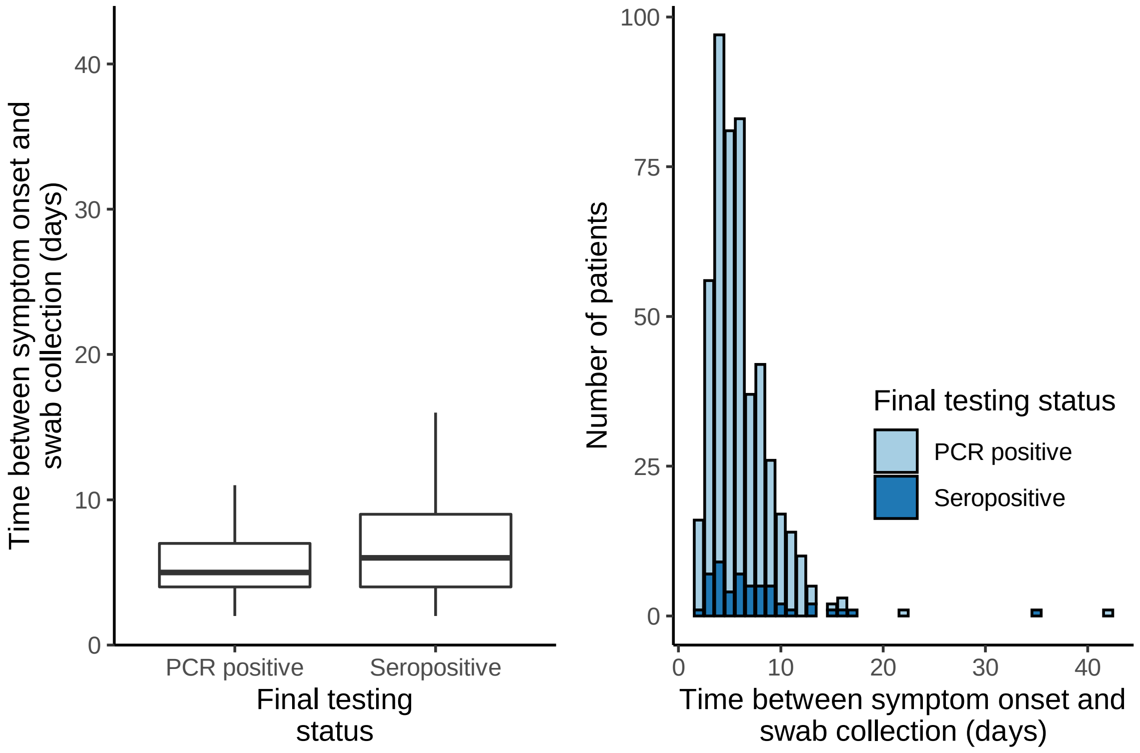

FIGURE 3 DISTRIBUTION OF RT-PCR AND SEROLOGY RESULTS ACCORDING TO THE TIME BETWEEN SYMPTOM ONSET AND SAMPLE COLLECTION IN 496 COVID-19 CASES IN A COMMUNITY SURVEILLANCE PROGRAM *[18]*

2.1.4 EPIDEMIOLOGY OF COVID-19

In June 2020, the number of confirmed cases of COVID-19 in the world exceeded 10 million people diagnosed and with more than half a million deaths. These data are updated daily by the World Health Organization on a specific website (https://covid19.who.int/) that also allows assessing the geographical expansion of the pandemic, which currently affects almost all countries and territories in the world. The Americas region is the hardest hit with almost half of the reported cases and deaths. The United States and Brazil are the hardest hit countries in the region and in the world in absolute numbers.
Considering the limitations that the pandemic imposes on the health system and the unavailability of tests for the diagnosis of all suspected cases, it was necessary to prioritize tests for symptomatic cases and, among them, those of greater clinical severity. For this reason, the actual number of SARS-CoV-2 infections and milder cases of COVID-19 is not yet precisely determined. However, some mathematical models allow estimates to be made in relation to cases that need hospitalization and that evolve to death, as shown in Table 1 [19].

In Brazil, the first case of COVID-19 was detected on February 25, 2020 and since then there have been reports of cases in all federative units being reported by June close to 1,400,000 cases and almost sixty thousand deaths, a number that have been increasing daily.

TABLE 1 ESTIMATES OF MORTALITY AND HOSPITALIZATION RATES BY AGE GROUP AMONG SARS-COV-2 INFECTED CASES IN CHINA *[19]*

Age group	Adjusted case fatality ratio (confirmed SARS-CoV-2 infection)	Infection fatality ratio (estimated SARS-CoV-2 infection)	Proportion of infected individuals hospitalized (estimated SARS-CoV-2 infection)
**0-9 years**	0·00260% (0·000312–0·0382)	0·00161% (0·000185–0·0249)	0·00% (0·00–0·00)
**10-19 years**	0·0148% (0·00288–0·0759)	0·00695% (0·00149–0·0502)	0·0408% (0·0243–0·0832)
**20-29 years**	0·0600% (0·0317–0·132)	0·0309% (0·0138–0·0923)	1·04% (0·622–2·13)
**30-39 years**	0·146% (0·103–0·255)	0·0844% (0·0408–0·185)	3·43% (2·04–7·00)
**40-49 years**	0·295% (0·221–0·422)	0·161% (0·0764–0·323)	4·25% (2·53–8·68)
**50-59 years**	1·25% (1·03–1·55)	0·595% (0·344–1·28)	8·16% (4·86–16·7)
**60-69 years**	3·99% (3·41–4·55)	1·93% (1·11–3·89)	11·8% (7·01–24·0)
**70-79 years**	8·61% (7·48–9·99)	4·28% (2·45–8·44)	16·6% (9·87–33·8)
**80 years and above**	13·4% (11·2–15·9)	7·80% (3·80–13·3)	18·4% (11·0–37·6)
**Total**	1·38% (1·23–1·53)	0·657% (0·389–1·33)	

2.1.5 IMMUNE RESPONSE AGAINST SARS-CoV-2 VIRUS

The population-wide immune response against SARS-CoV-2 is not yet well established, although preliminary results indicate that it resembles other coronaviruses in terms of heterogeneity
and duration. One of the studies that showed this variability better compared 37 symptomatic patients and 37 asymptomatic patients in the acute period and in convalescence 8 weeks later [20]. In this study, it was found that viral excretion was more prolonged among asymptomatic patients. On the other hand, the antibody response, both IgG and IgM, was significantly higher among symptomatic people. In 93% of

asymptomatic patients and 97% of symptomatic patients, there was a significant drop in IgG titers after 8 weeks, a fall of 71% and 76%, respectively. There was an analogous reduction in the neutralizing antibody test. In the convalescent sample, 40% of asymptomatic and 13% of symptomatic individuals started to test negative for IgG despite the previous infection confirmed virologically. These results demonstrate more dramatic IgG drops than those documented with SARS-CoV-1. Milder cases of SARS-CoV-2 infection appear to be restricted to the upper respiratory tract, with less opportunity to stimulate a systemic immune response, in contrast to more symptomatic cases where the infection could compromise more organs and generate an amplified immune response.

In severe cases, the drop in lymphocytes appears to play a fundamental role. The invasion of SARS-CoV-2 to different organs that have ACE2 receptors does not seem to be controlled by the neutralizing antibody response, possibly due to a specific drop in B lymphocyte levels. There is no explanation for this drop, but IL-6 seems to have a important role in the pathogenesis which has motivated the experimental use of monoclonal antibodies against this cytokine to treat severe forms of COVID-19. It is also worth noting that the levels of neutrophils and D-dimer are higher in those patients who evolve to death, which indicates an exaggerated inflammatory response with intravascular coagulation, manifested even with thrombotic and vasculitis. The recovery of the antibody production capacity could be one of the key factors for recovery in severe cases of COVID-19 [21].

2.1.6 DEVELOPMENT OF VACCINES AGAINST COVID-19

Since the emergence of COVID-19, several efforts have been made to develop a vaccine with speed never seen before. Different technologies have been used, and in less than 6 months, different vaccine candidates had already reached the clinical stage. Some of them, such as the vaccine developed by Sinovac, benefited from previous development against SARS-CoV-1 that had reached clinical phase I in 2004 [22], while others were adaptations of developments against MERS-CoV, as is the case of the vaccine using the ChAdOx1 vector that had its clinical phase I in 2018 [23]. In general, the most used technologies are inactivated vaccines, viral vectors (adenovirus, measles and others) that can be replicating or not, nucleic acids (DNA and RNA), protein and subunit by recombination or synthesis, and virus-like particles (VLP). Most vaccines are based solely on Spike protein or regions of that protein, in particular RBD [24]. A very limited number of vaccine candidates have other viral proteins represented which represents a potential risk for those that only generate a response against the Spike protein or its regions in case of mutations or that the response to that protein is insufficient. Hence part of the interest in having inactivated vaccines and other options that represent different viral proteins.

One of the theoretical risks of vaccines against COVID-19 comes from histopathological findings of challenge animal models after immunization with vaccines against other coronaviruses, SARS-CoV-1 and MERS-CoV. In these cases, immunized animals that were experimentally exposed to the respective coronavirus had an inflammatory reaction in the pulmonary parenchyma of eosinophilic predominance. This finding raised concerns about the possibility of potentiation of respiratory disease associated with vaccine in vaccines for coronavirus [25]. So far, this phenomenon has not been reported in vaccine candidates for COVID-19. However, it is recommended that before starting clinical trials with humans, challenge experiments are carried out on experimental animals to verify the absence of this anomalous pulmonary inflammatory response in experimental SARS-CoV-2 infection after immunization [26]. It should be noted that the aluminum hydroxide adjuvant is postulated to have a protective effect against this phenomenon, in addition to stimulating a potent humoral response of neutralizing antibodies [25,27–29]

2.1.7 DEVELOPMENT OF THE COVID-19 ADSORBED VACCINE (INACTIVATED) PRODUCED BY SINOVAC (CORONAVAC)

The adsorbed vaccine COVID-19 (inactivated) is developed by the company Sinovac Life Sciences Co., Ltd. (hereinafter called Sinovac). The adsorbed vaccine COVID-19 (inactivated) is derived from the new coronavirus SARS-CoV-2 (strain CZ02) and grown in an African green monkey kidney cell (Vero Cell), followed by culture, harvesting, inactivation, concentration, purification and adsorption of aluminum hydroxide. It contains the new coronavirus (SARS-CoV-2), hydroxide aluminum, disodium hydrogen phosphate, sodium dihydrogen phosphate and sodium chloride. The vaccine is free of preservatives. The final packaging is a vial or syringe filled with an extractable volume of 0.5 mL. Low, medium and high dosages there are 300SU, 600SU and 1200SU, respectively The COVID-19 adsorbed vaccine (inactivated) is indicated for active immunization against the disease caused by the new coronavirus.

2.1.7.1 Summary of Development of Vaccine Strains

The CZ02 strain is isolated from a throat swab from patients infected with SARS-CoV-2. After being isolated, the strain is identified by nucleotide sequencing. The three-layer seed lots were established and tested following the Manufacturing and Quality Control requirements for the adsorbed vaccine COVID-19 (inactivated), the results obtained so far were in accordance with the quality parameters. The prepared seed lots can meet the demands of the development of the adsorbed vaccine COVID-19 (inactivated).

2.1.7.2 Non-clinical studies

Several safety trials have been carried out in animals, including (single dose toxicity, active systemic anaphylaxis test and repeated dose toxicity test), as well as studies of the efficacy and immunogenicity of the vaccine.

2.1.7.2.1 Safety studies

Single-dose toxicity tests were performed on rats, active systemic anaphylaxis tests on guinea pigs, repeated dose toxicity tests on rats and Macaca fascicularis and no abnormal clinical reactions were observed in these tests, the body weight in all groups of animals showed a normal tendency to increase during the study period indicating that the vaccine has good safety in animals.

2.1.7.2.2 Immunogenicity studies

Immunogenicity tests in rats and mice were conducted to assess the neutralizing antibody and IgG levels of different SARS-CoV-2 vaccines (with and without adjuvant) at different points in time, using different immunization schedules, different immunization routes (intraperitoneal immunization of mice and intramuscular immunization of rats) with different doses of vaccines in order to determine the vaccine formulation, dosage and immunization schedule. The results demonstrated favorable immunogenicity of the SARS-CoV-2 vaccine with adjuvant, and at the dosage of 300 SU / dose, 600 SU / dose and 1200 SU / dose, and the two-dose immunization schedule was selected for the clinical study.

2.1.7.2.3 Viral challenge studies

In a viral challenge study conducted in rhesus macaques, twelve animals were randomized into 4 groups to receive 2 medium or high doses of the vaccine (Day 0.14) or 3 doses of adjuvant (alum, Day 0.7, 14) or the same volume of saline. The viral challenge started from Day 22 and Day 23 after vaccination of groups with medium or high dose of the vaccine, respectively. The results indicated that the inactivated SARS-CoV-2 vaccine has a significant protective effect. In addition, no antibody-dependent enhancement (from English, antibody-dependent enhancement or ADE) was observed during the study [30].

2.1.7.3 Clinical studies

2.1.7.3.1 Studies in adults

Objective: To evaluate the safety, tolerability and preliminary immunogenicity of different doses of vaccine administered in different immunization schedules in adults to determine the appropriate dosage and immunization schedule for advanced clinical evaluations.

Design: Phase I / II randomized, double-blind, placebo-controlled clinical study of 144 healthy adults in Phase I and 600 healthy adults in Phase II aged 18-59 years assigned to receive 2 doses of vaccine at a medium (3 ug. equivalent to 600 SU) or high dose (6 ug, equivalent to 1,200 SU) and discharge or placebo in an emergency schedule (Day 0.14) or routine schedule (Day 0.28) to assess safety and immunogenicity.

Safety: In the Phase I study the incidence rates for solicited adverse reactions in the high dose, medium dose and placebo groups were 37.50%, 25.00% and 8.33%, respectively.

The incidence rates of adverse reactions in the Phase 2 are described in Table 2, separately for the high dose, medium dose and placebo groups. The reaction rates were higher in the two vaccine groups than in the placebo group, however, no statistically significant difference was found between the medium and high dosage groups. The adverse reactions were mostly Grade 1 and no serious adverse reactions occurred [31].

Table 2. Incidence of adverse reactions in the phase II study of the adsorbed vaccine COVID-19 (inactivated) [31]

	0 & 14 days schedule			0 & 28 days schedule		
**Adverse Reactions**	**Medium dose (N=120**	**High dose (N=120)**	**Placebo (N=60)**	**Medium dose (N=120**	**High dose (N=120)**	**Placebo (N=60)**
**Severity**	33.3	35	21.7	19.2	19.2	18.3
Grade 1	32.5	35	21.7	19.2	19.2	18.3
Grade 2	3.3	0.83	0	0	2.5	0
**Solicited**	32.5	33.3	21.7	19.2	19.2	16.7
Systemic	15	12.5	15	11.7	10	10
Fever (axillary temp)	3.3	0.8	1.7	3.3	3.3	1.7
Acute allergic reaction	0	0.8	0	0.8	0	0
Mucosal and skin reactions	0	0	0	0	0	0
Diarreha	5	0.8	1.7	0.8	2.5	1.7
Anorexia	0.8	0	0	0	0.8	0
Vomiting	0.8	0	0	0	0	0
Nausea	1.7	2.5	0	1.7	0	0
Myalgia	2.5	1.7	1.7	1.7	3.3	5
Headache	1.7	3.3	1.7	2.5	0.8	0
Coughing	0	1.7	0	0.8	0	0
Fatigue	3.3	5.8	10	8.3	2.5	3.3
Local	21.7	25.8	10	10	12.5	10
Pain	20.8	25	10	10	10.8	10
Induration	0	0.8	0	0	0	0
Edema	1.7	2.5	0	0	0.8	1.7
Redness	1.7	1.7	0	0	0.8	0
Erithema	0	0	0	0	0	0

Pruritus	0.8	0.8	0	0	0.8	0
**Unsolicitted**	2.5	2.5	0	0	0.8	1.7
Neruologic	0.8	0.8	0	-	--	--
Somnolence	0	0.8	0	--	--	--
Dizziness	0.8	0	0	--	--	--
Systemic and site of injection	1.7	1.7	0	0	0.8	1.7
Chest pain	0	0.8	0	--	--	--
Depigmentation	0.8	0	0	0	0.8	1.7
Hiposthesia	0.8	0.8	0	--	--	--
Cardiac	0	0.8	0	--	--	--
Palpitations	0	0.8	0	--	--	--

Immunogenicity: among Phase I participants, the seroconversion rates in the high dose, medium dose and placebo groups were 50.00%, 45.83% and 0.00%, respectively. The medium geometric titer - GMT in the high dose, medium dose and placebo groups were 7.7, 5.6 and 2.0, respectively. The seropositivity rates for IgG antibody in the high dose, medium dose and placebo groups were 95.83%, 75.00% and 8.33%, respectively, while IgM seropositivity was 45.83%, 20.83% and 0.00%, respectively. Participants in the placebo group failed to elicit an immune response.

In the Phase II study, the seroconversion rates in the high dose, medium dose and placebo groups were 98.32%, 92.37% and 3.33%, respectively. The GMT in the high dose, medium dose and placebo groups were 34.5, 27.6 and 2.3, respectively. The GMT the high and medium dose groups were comparable.

Conclusion: In conclusion, all data from Phase I / II clinical trials indicated favorable safety and immunogenicity with the two-dose scheme of the adsorbed vaccine COVID-19 (inactivated). The GMT in the high and medium dose groups were comparable. Based on the results, we did not observe an explicit cellular immunity induced by the adsorbed vaccine COVID-19 (inactivated), and further studies should be performed. There were no significant changes in inflammatory factors, which indicates a small risk of immunopathology induced by the adsorbed vaccine COVID-19 (inactivated).

TABLE 10. SEROCONVERSION RATES AS MEASURED BY NEUTRALIZATION AND ELISA IGG [31]

Test	0 & 14 days schedule			0 & 28 days schedule		
	Medium dose	High dose	Placebo	Medium dose	High dose	Placebo
**Neutralizing Antibodies**						
14 days after vaccination	92.4<br>(86.0, 96.5)	98.3<br>(94.1, 99.8)	3.3<br>(0.4, 11.5)	--	--<br>--	
28 days after vaccination	94.1<br>(88.2, 97.6)	99.2<br>(95.4, 100.0)	0.0 (0.0, 6.0)	97.4<br>(92.7, 99.5)	100<br>(96.9, 100.0)	0<br>(0.0, 6.1)
**ELISA IgG Antibodies**						
Pre-vaccination	2.6 (0.5, 7.4)	2.5 (0.5, 7.3)	1.8 (0.1, 9.6)	1.7 (0.2, 6.0)	0.9 (0.0, 4.7)	1.7 (0.0, 9.1)
14 days after vaccination	96.5	100	0	--	--	--

	(93.9, 99.8)	(96.9, 100.0)	(0.0, 6.4)			
28 days after vaccination	97.4	100	0	99.2	100	6.8
	(92.5, 99.5)	(96.9, 100.0)	(0.0, 6.3)	(95.3, 100.0)	(96.9, 100.0)	(1.9, 16.5)

2.1.7.3.2 Studies in the elderly

Objective: To evaluate the safety and preliminary immunogenicity of different doses of the vaccine in a healthy population, over 60 years of age.

Design: A phase I / II randomized, double-blind, placebo-controlled clinical study with a healthy population of 72 participants in Phase I and a healthy population of 350 participants in Phase II is evaluating the safety and immunogenicity in participants over 60 years of age who were assigned to receive two doses of vaccine or placebo on a routine schedule (Days 0 and 28).

Results: This clinical study is ongoing.

2.1.7.4 Vaccine-induced cellular immune response

In the Phase I clinical study in healthy adults, the vaccine-induced cellular immune response was assessed using a specific T-cell assays (IFN-γ, IL-6, IL2 and TNF- α) at 14 days after vaccination.

The study included 72 participants. No specific T cell responses were observed before vaccination. Fourteen (14) days after the first vaccination, positive IFN-γ rates in the high dose, medium dose and placebo groups were 20.83%, 45.83% and 8.33%, respectively. There were no statistically significant differences after the first dose in the levels of IL-6 and IL2. Regarding TNF-α, there was a two-fold increase in the high-dose group. The totality of the data did not provide evidence of a dose-response relationship. Based on these results, no conclusive cellular immunity induced by the adsorbed vaccine COVID-19 (inactivated) was observed and further studies should be performed.

2.2 OBJECTIVES

2.2.1 PRIMARY OBJECTIVES

1. To evaluate the efficacy of two doses of the adsorbed vaccine COVID-19 (inactivated) produced by Sinovac in symptomatic individuals, with virological confirmation of COVID-19, two weeks after the second vaccination, aged 18 years or older who work as healthcare professionals in direct contact care of people with possible or confirmed COVID-19 cases
2. To describe the occurrence of adverse reactions associated with the administration of each of two doses of the adsorbed vaccine COVID-19 (inactivated) produced by Sinovac up to one week after vaccination in Adults (18-59 years of age) and Elderly (60 years of age or more) who work as healthcare professionals in direct contact care of people with possible or confirmed COVID-19 cases.

2.2.2 SECONDARY OBJECTIVES

Efficacy evaluations

1. To evaluate the efficacy of two doses of the adsorbed vaccine COVID-19 (inactivated) produced by Sinovac in symptomatic individuals, with virological confirmation of COVID-19, two weeks after the second vaccination, aged 18 years or who work as healthcare professionals in direct

contact care of people with possible or confirmed COVID-19 cases according to previous exposure to SARS-CoV-2.

2. To evaluate the efficacy of at least one dose of the adsorbed vaccine COVID-19 (inactivated) produced by Sinovac in symptomatic individuals with virological confirmation of COVID-19 aged 18 years or older who work as healthcare professionals in direct contact care of people with possible or confirmed COVID-19 cases from two weeks after the first vaccination.
3. To evaluate the efficacy of two doses of a COVID-19 adsorbed vaccine (inactivated) produced by Sinovac in asymptomatic and symptomatic infections by SARS-CoV-2 detected serologically or virologically, in individuals aged 18 years or older who work as healthcare professionals in direct contact care of people with possible or confirmed COVID-19 cases from two weeks after the first vaccination.
4. To evaluate the efficacy of the adsorbed vaccine COVID-19 (inactivated) produced by Sinovac in severe cases of COVID-19 confirmed virologically in adults in individuals aged 18 years or older who work as healthcare professionals in direct contact care of people with possible or confirmed COVID-19 cases from two weeks after receiving the second vaccination.

Safety evaluations

1. To describe the onset of solicited and unsolicited adverse reactions associated with the administration of each of the two doses COVID-19 adsorbed vaccine (inactivated) produced by Sinovac within four weeks after vaccination adult (18-59 years of age) and elderly (60 years of age or older) participants.
2. To describe the occurrence of severe cases of COVID-19 in participants who have received at least one dose of the adsorbed vaccine COVID-19 (inactivated) produced by Sinovac.
3. To describe the occurrence of Adverse Events of Special Interest in participants who have received at least one dose of the adsorbed vaccine COVID-19 (inactivated) produced by Sinovac.

Immunogenicity evaluations

1. To evaluate the immune response to vaccination in a subset of participants two weeks after the administration of each dose of the adsorbed vaccine COVID-19 (inactivated) produced by Sinovac in adult (18-59 years of age) and elderly (60 years of age or older) participants.
2. To evaluate the immune response to cell-mediated vaccination in a subset of participants before each vaccination and at two and four weeks (and potentially other timepoints) after administration of the second dose of the adsorbed vaccine COVID-19 (inactivated) produced by Sinovac in adult (18-59 years of age) and elderly (60 years of age or older) participants.
3. To evaluate the presence of antibodies against SARS-CoV-2 before and two weeks after the administration of the second dose of the adsorbed vaccine COVID-19 (inactivated) produced by Sinovac in adults (18-59 years of age) and elderly (60 years of age or older) participants.

2.2.3 EXPLORATORY OBJECTIVES

1. To evaluate the immune response in incident COVID-19 cases occurred in the study.
2. To examine the level of antibodies induced by the adsorbed vaccine COVID-19 (inactivated) produced by Sinovac in additional laboratory assays to investigate a possible correlate of protection.
3. To characterize the immune response up to one year of the study in a subgroup of participants.
4. To determine potential interactions between vaccination and other underlying medical conditions, for example, diabetes, obesity, dyslipidemia, hypertension and cardiovascular disease.
5. To describe the immune response in participants with previous SARS-CoV-2 infection who received the adsorbed vaccine COVID-19 (inactivated) produced by Sinovac.

6. To evaluate the efficacy of two doses of a COVID-19 adsorbed vaccine (inactivated) produced by Sinovac in asymptomatic and symptomatic recurrent infections by SARS-CoV-2 detected serologically and virologically, two weeks after the second vaccination in people with previous documented infection.
7. To evaluate the efficacy of two doses of a COVID-19 adsorbed vaccine (inactivated) produced by Sinovac in cases of serious adverse event due to all causes and by COVID-19, from two weeks after the second vaccination.
8. To evaluate the efficacy of two doses of a adsorbed vaccine COVID-19 (inactivated) produced by Sinovac in cases of death from all causes and by COVID-19, from two weeks after the second vaccination

2.3 GENERAL STUDY DESIGN

This is a Phase III, randomized, multicenter, endpoint driven, double-blind, placebo-controlled clinical trial to assess the efficacy and safety of the adsorbed vaccine COVID-19 (inactivated) produced by Sinovac.

Healthy participants and / or participants with clinically controlled disease, of both genders, 18 years of age or older, working as who work as healthcare professionals in direct contact care of people with possible or confirmed COVID-19 cases. Participation of pregnant women and those who are breastfeeding, as well as those intending to become pregnant within three months after vaccination will not be allowed. Participants will only be included after signing the voluntary Informed Consent Form and ensuring they undergo screening evaluation and conform with all the inclusion and exclusion criteria.

The adsorbed vaccine COVID-19 (inactivated) produced by Sinovac (product under investigation) will be compared to placebo. Voluntary participants will be randomized to receive two intramuscular doses of the investigational product or the placebo, in a 1: 1 ratio, stratified by age group (18 to 59 years and 60 years or more) and will be monitored for one year by active surveillance of COVID-19. Two databases will be established according to the age groups: one for adults (18-59 years) and one for the elderly (60 years of age or older).

The criterion to determine that the vaccine efficacy is satisfactory will be to reach a protection level of at least 50%, in accordance to the criterion proposed by the World Health Organization and the FDA. Success in this criterion will be defined by sequential monitoring with adjustment of the lower limit of the 95% confidence interval above 30% for the primary efficacy endpoint.

In order to describe induced immune responses and explore a potential humoral immunity maker correlated with vaccine protection, a comparison will be made between the immune response developed by participants who developed the disease (cases) and a subgroup of 10% of participants who did not develop the disease (controls). If necessary, the subgroup can be expanded to better support any conclusions. All participants will have samples collected before, and two weeks after and four weeks after each dose of the vaccine to allow for the selection described above. The immune response will be assessed by neutralizing antibody titers and ELISA tests for SARS-CoV-2 proteins. These tests will be repeated on samples from weeks 13, 26, 39 and 52.

In addition, a smaller subgroup of participants, the first 60 by age group at the first research center, will be assessed for markers of cellular immunity before each vaccination and two and four weeks after the second vaccination. The collected cells will be stimulated by “megapools” of SARS-CoV-2 peptides

recognizable by HLA class I and II [32] to detect their cytokine response. These tests can be repeated on samples from weeks 13, 26, 39 and 52.

3 METHODS

3.1 STUDY CENTERS

The study will be carried out in clinical research centers accessible to health professionals working in specialized areas for the care of patients with COVID-19. The study centers will act in an integrated way allowing the monitoring of the participants and obtaining the data as described in this protocol. The contact details of the study centers can be found in Annex A.

3.2 PARTICIPANTS

Healthy participants and / or participants with clinically controlled comorbidities, of both sexes and aged 18 years or older who work as health professionals in units specialized in the treatment of COVID-19 may be included in the study. The entry criteria (inclusion and exclusion) in the study should be checked before the administration of each of the two doses of the immunization schedule. Participants must meet all the inclusion criteria and not meet any of the exclusion criteria, described below:

3.2.1 INCLUSION CRITERIA

a. Adults 18 years of age or older;
b. Healthcare professionals who work in direct contact care of people with possible or confirmed COVID-19 cases;
c. Agree to periodic contacts by telephone, electronic means and home visits;
d. Demonstrate intention to participate in the study, documented by the participant's signing the Voluntary and Informed Consent Term.

3.2.2 EXCLUSION CRITERIA

a. For females: Pregnancy (confirmed by positive β-hCG test), breastfeeding and / or expressing intention to have sexual practices with reproductive potential without using contraceptive methods in the three months following vaccination;
b. Evidence of uncontrolled neurological, cardiac, pulmonary, hepatic or renal disease, according to anamnesis or physical examination. Significant changes in treatment or hospitalizations due to worsening of the condition in the last three months are indicators of uncontrolled disease;
c. Diseases with impaired immune system including: neoplasms (except basal cell carcinoma), congenital or acquired immunodeficiencies and autoimmune diseases not controlled according to anamnesis or physical examination. Significant changes in treatment or hospitalizations due to worsening of the condition in the last three months are indicators of uncontrolled disease;
d. Behavioral, cognitive or psychiatric illness that, in the opinion of the principal investigator or his medical representative, affects the participant's ability to understand and collaborate with the requirements of the study protocol;
e. Any alcohol or drug abuse in the last 12 months prior to inclusion in the study that has caused medical, professional or family problems, as indicated by clinical history;
f. History of severe allergic reaction or anaphylaxis to the vaccine or components of the study vaccine;
g. History of asplenia;

h. Participation in another clinical trial with product administration under investigation during the six months prior to its inclusion in the study or scheduled participation in another clinical trial in the two years following inclusion;
i. Previous participation in a COVID-19 vaccine evaluation study or previous exposure to a COVID-19 vaccine;
j. Use of immunosuppressive therapies six months prior to inclusion in the study or its scheduled use within two years of inclusion. Immunosuppressive therapies will be considered: antineoplastic chemotherapy, radiation therapy, immunosuppressants to induce tolerance to transplants, among others.
k. Have received an immunosuppressive dose of corticosteroids in the last three months prior to inclusion in the study or scheduled administration of an immunosuppressive dose of corticosteroids for the three months following inclusion in the study. The dose of corticosteroids considered immunosuppressive is equivalent to prednisone at a dose of 20 mg / day for adults, for more than a week. The continuous use of topical or nasal corticosteroids is not considered immunosuppressive;
l. Have received blood products (transfusions or immunoglobulins) in the last three months before inclusion in the study, or scheduled administration of blood products or immunoglobulin in the two years following inclusion in the study;
m. Suspected or confirmed fever within 72 hours prior to vaccination or axillary temperature greater than 37.8 ° C * on the day of vaccination (inclusion may be postponed until the participant completes 72 hours without fever);
n. Possible or confirmed case of COVID-19 on the day of vaccination (vaccination can be postponed until the participant completes 72 hours without symptoms or the diagnosis is ruled out);
o. Have received vaccine with live attenuated virus in the last 28 days or inactivated vaccine in the last 14 days prior to their inclusion in the study, or have immunization scheduled for the first 28 days after their inclusion in the study;
p. History of bleeding disorders (for example, deficiency of clotting factors, coagulopathy, platelet dysfunction), or previous history of bleeding or significant bruising after IM injection or venipuncture;
q. Any other condition that r. Any other condition that, in the opinion of the principal investigator or his medical representative, could jeopardize the safety or rights of a potential participant or that would prevent him from complying with this protocol.

** Temperature measured with a skin thermometer using a temporal scanner is considered equivalent to the axillary temperature.*

3.3 ENDPOINTS

3.3.1 PRIMARY ENDPOINTS

1. The primary efficacy endpoint is the incidence of symptomatic cases of virologically confirmed COVID-19 two weeks after the second vaccination. The virological diagnosis will be confirmed by detection of SARS-CoV-2 nucleic acid in a clinical sample.
2. The primary safety endpoint is the frequency of solicited and unsolicited local and systemic adverse reactions during the period of one week after vaccination according to age group in adult (18-59 years old) and elder (60 years of age or older) subjects. Adverse reactions are defined as adverse events that have a reasonable causal relationship to vaccination.

3.3.2 SECONDARY ENDPOINTS

Secondary efficacy endpoints:

1. The incidence of symptomatic cases of virologically confirmed COVID-19 two weeks after the second vaccination according to previous exposure to SARS-CoV-2. Previous exposure to SARS-

CoV-2 will be demonstrated by detection of nucleic acid of SARS-CoV-2 in previous test or on the vaccination day, or positive antibody test against SARS-CoV-2 on the vaccination day.

2. The incidence of cases of virologically confirmed COVID-19 after administration of the adsorbed (inactivated) COVID-19 vaccine produced by Sinovac, two weeks after the first vaccination.
3. The incidence of SARS-CoV-2 infections, symptomatic or asymptomatic, detected serologically and / or virologically, two weeks after the second vaccination. Serological confirmation of SARS-CoV-2 infections will require a fourfold increase in the level of IgG titers in validated serological assays.
4. The incidence of severe cases of COVID-19 confirmed virologically two weeks after the administration of the second dose of the adsorbed vaccine COVID-19 (inactivated).

Secondary safety endpoint:

5. The frequency of adverse reactions to the vaccine, local and systemic, solicited and unsolicited, after each of the two doses of the vaccination schedule within the period of four weeks after vaccination, according to the age group, adults (18-59 years old) and elder (60 years or older) subjects.
6. The frequency of severe COVID-19 cases in participants who received at least one dose of the experimental product.
7. The frequency of cases of Adverse Events of Special Interest in participants who received at least one dose of the experimental product.

Secondary immunogenicity endpoint:

8. Seroconversion rates in the second week after each vaccination according to age group (Adults 18-59 years old and Elderly people 60 years of age or older).
9. Cell-mediated immune response in a subgroup of participants before each vaccination and two and four weeks after the second vaccination according to age group (Adults 18-59 years and Elders 60 years of age or older).
10. The frequency of detection of antibodies against SARS-CoV-2 before and two weeks after the administration of the second dose of the adsorbed vaccine COVID-19 (inactivated) produced by Sinovac in Adults (18-59 years of age) and Elderly (60 years of age) age or older). The evaluation will be carried out in all research participants through validated ELISA and / or Chemiluminescence methodology.

3.3.3 EXPLORATORY ENDPOINTS

1. Antibody titers among incident COVID-19 cases.
2. Antibody titers to SARS-CoV-2 in additional laboratory assays, including novel approaches). Samples from study subjects may be shared with specialized immunology laboratories.
3. Immune response in up to one year in a subgroup of participants.
4. Incidence of cases of COVID-19 confirmed virologically, two weeks after vaccination, in participants with underlying medical conditions, for example, diabetes, obesity, dyslipidemia, hypertension and cardiovascular disease.
5. Immune response and frequency of COVID-19 cases in participants with previous SARS-CoV-2 infection who received the adsorbed (inactivated) COVID-19 vaccine produced by Sinovac.
6. The incidence of SARS-CoV-2 recurrent infections detected serologically and / or virologically, from two weeks after the second vaccination in people with previous infection documented by virological or serological diagnosis. Serological confirmation of SARS-CoV-2 infections will be by a four-fold increase in the level of IgG titers in validated serological assays.

7. The incidence of serious adverse events by all causes and by COVID-19 confirmed virologically from two weeks after the administration of the second dose of the adsorbed vaccine COVID-19 (inactivated).
8. The density of incidence of cases of death from all causes and by COVID-19 confirmed virologically from two weeks after the administration of the second dose of the adsorbed vaccine COVID-19 (inactivated).

3.3 INTERVENTIONS

3.4.1 FINAL COMPOSITION AND FORMULATIONS OF THE COVID-19 ADSORBED VACCINE (INACTIVATED) PRODUCED BY SINOVAC

The vaccine developed by Sinovac Research & Development Co., Ltd. is a preparation made from the new coronavirus (strain CZ02) grown in a kidney cell line of an African green monkey (Cell Vero). Following cultivation and harvesting, the viral suspension is inactivated, concentrated, purified and adsorbed by aluminum hydroxide. It contains the SARS-CoV-2 coronavirus, aluminum hydroxide, disodium hydrogen phosphate, sodium dihydrogen phosphate, sodium chloride. Its commercial name is: CoronaVac 0.5mL / dose containing 600SU of SARS-CoV-2 virus antigen. The vaccine is injected intramuscularly, and the vaccination schedule is two doses, with an interval of two weeks between them.

The SARS-CoV-2 virus is inactivated twice to ensure biosafety. Inactivation is carried out with the addition of β-propiolactone in the virus collection liquid in the proportion of 1: 4000 (V / V) during 12-24 hours of inactivation at 2-8 ° C. The liquid collected from inactivation is then discarded with β-propiolactone in the proportion of 1: 4000 and formaldehyde solution in the proportion of 1: 2000 for further treatment at 2-8 ° C for 2-8 hours, obtaining the liquid with SARS-CoV-2 inactivated.

The inactivation process parameter defined for inactivating 9 batches of inactivation liquid and performing the inactivation check must be followed. The results demonstrate that following the defined inactivation process, the SARS-CoV-2 virus can be completely inactivated which demonstrates that the inactivation process is stable.

3.4.1.1 Adsorbed (inactivated) COVID-19 vaccine produced by SINOVAC

The vaccine contains inactivated SARS-CoV-2 virus, aluminum hydroxide, disodium hydrogen phosphate, sodium dihydrogen phosphate and sodium chloride.

3.4.1.1.1 Pharmaceutical Form and Packaging

The final product will be supplied in a pre-filled syringe containing 0.5 ml of solution for injection that corresponds to a dose of the vaccine.

3.4.1.1.2 Dose / Administration / Storage

The vaccine storage temperature, as indicated on the vial label, is 2 to 8 ° C. The vaccine dose corresponds to 0.5 ml. The recommended site of administration is the deltoid muscle of the arm by intramuscular injection. The immunization schedule is two doses with an interval of two weeks.

3.4.1.1.3 Vaccine lots

In this study, a single vaccine production batch will be used.

3.4.1.2 Indications for use / Contraindications / Warnings / Precautions

Until licensed for a specific indication, the COVID-19 adsorbed vaccine (inactivated) produced by Sinovac is for use in research and should be administered only to subjects participating in a clinical study conducted in accordance with applicable Federal Regulations and the criteria for inclusion and exclusion specified in a Research Protocol previously approved by the responsible ethical and regulatory bodies.

As many of the effects related to the administration of this experimental vaccine are unknown, there may be an increased risk of the occurrence of adverse events if the subjects are given other experimental drugs or under investigation. For this reason, it is essential that the subjects receiving the vaccine viruses do not participate in any other studies with products under investigation while conducting this study.

3.4.1.3 Final composition and formulation of the placebo

3.4.1.3.1 Composition

The placebo contains aluminum hydroxide, disodium hydrogen phosphate, sodium dihydrogen phosphate, 0.5mL sodium chloride / dose, intramuscular injection, two doses administered two weeks apart.

3.4.1.3.2 Pharmaceutical Form and Packaging

The final product will be supplied in a pre-filled syringe containing 0.5 ml of solution for injection that corresponds to a dose of the placebo.

3.4.1.3.3 Dose / Administration / Storage

The storage temperature of the placebo, as indicated on the bottle label, is 2 to 8 ° C. The vaccine dose corresponds to 0.5 ml. The recommended site of administration is the deltoid muscle of the arm by intramuscular injection. The placebo immunization schedule is two doses with an interval of two weeks.

3.4.1.4 Product label

The labels of the investigational product will be prepared according to the current regulation. Samples of these labels can be found in Annex B.

3.4.2 PERMITTED, NOT RECOMMENDED AND PROHIBITED CONCOMITANT INTERVENTIONS OR TREATMENT SCHEMES

There are no drugs prohibited in this protocol. However, the use of the following products is not recommended for four weeks (28 days) following the administration of the product under investigation:

a. Any drug or vaccine under investigation other than that used in this study;
b. Chronic administration (≥14 days) of systemic corticosteroids
c. Any licensed vaccine;
d. Immunoglobulins and / or any blood products.

If the use of any of these products in the first four weeks (28 days) after vaccination is essential, this must occur after medical advice and the event must be noted in the individual record of the research participant.

If this use occurred after the first dose and before the second dose, the second dose will not be administered.

If the research center team identifies the need for additional immunization of the participant, according to the recommendations of the Ministry of Health, the participant may be referred to be vaccinated after the period of four weeks (28 days) after the vaccination provided for in this protocol.

The use of other vaccines, even if experimental, aimed at preventing COVID-19 is not recommended during the follow-up period of the study. If so, it should be noted in the participant's individual record

3.4.3 RISK OF PREGNANCY FOR FEMALE PARTICIPANTS

All female participants will be considered with the potential to become pregnant, except those who have not yet had their first period, have a documented history of hysterectomy, tubal ligation or are in menopause (12 months of amenorrhea after their last menstrual period).

For female participants with the potential to become pregnant, the study doctor will assess the need to start using a contraceptive method before being included in the study and maintaining its use until week 4 (day 28) after receiving the last vaccination. Contraceptive methods will not be mandatory if the volunteer, or her legal representative, declares her exempt from the risk of becoming pregnant, either for not engaging in sexual practices or for exercising them in a non-reproductive manner, until week 4 after the last vaccination.

This discussion should cover the efficacy of the different contraceptive methods according to the following classification proposed by the Medical Eligibility Criteria for Contraceptive Use at the United States Centers for Disease Control and Prevention [33], Most effective methods (less than one episode of pregnancy per hundred women-years):

- implant;
- intrauterine copper device
- levonorgestrel intrauterine system;
- vasectomy;
- female sterilization;
- effective methods (6 to 12 episodes of pregnancy per 100 woman-years):
- monthly injectables;
- combined or progestogen pills;
- vaginal ring;
- adhesive;
- diaphragm with spermicide

Ineffective methods (18 or more episodes of pregnancy per 100 women-years):

- the male condom;
- the female condom;
- coitus interrupted;
- sponge;
- abstinence fertile periods
- spermicide isolated.

This study may refer volunteers to family planning who wish to do.

3.4.4 PROCEDURES FOR VERIFYING TREATMENT ADHESION

The treatment of this research protocol is the administration of the product under investigation that will be carried out in the research centers by a member of the study team.

3.5 PROCEDURES FOR PARTICIPANTS

3.5.1 FOLLOW-UP OF PARTICIPANTS

In all, each participant will be followed blindly for a period of one year after their inclusion in the study with the objective of assessing the occurrence of cases of COVID-19 and their immune response throughout this period.

3.5.2 STUDY PROCEDURES

After the study is released, potential participants who present themselves will be received by the local team of researchers and will be asked about their age group, place of residence and underlying diseases. Participation in observational studies (eg cohorts) will not be an impediment to becoming a volunteer in this study because there are no interventions in such studies that may interfere with the present clinical trial.

If there is no initial impediment to participation in the study, the researchers will provide general information about the study and apply the Informed Consent Form (ICF) in accordance with current legislation. The informed consent process is described in Section 4.3.1. If there is a temporary condition that constitutes an exclusion criterion, the potential participant can be called up again for the inclusion visit when that condition does not exist.

A participant will be considered to be included in the study at the time they are vaccinated (Day 0).

3.5.3 DESCRIPTION OF THE STUDY PROCEDURES BY VISIT

3.5.3.1 Vaccination visits (V1 and V2)

In vaccination visits (V1 and V2), the inclusion and exclusion criteria and their eligibility will be checked through clinical history and physical examination in V1 and will be reviewed in V2. Once considered eligible, the participant will be vaccinated on this same visit and this will be considered Day 0. V1 can be divided, making it possible to present and sign the informed consent form, as well as obtaining some demographic, anthropometric and medical history data up to three days before vaccination. Vaccination visit 2 (V2) is scheduled to occur two weeks (+2 window week) after vaccination visit 1 (V1). The vaccination visit is mandatory for all volunteers.

Procedures:

a. Present the study to the potential participant, perform the informed consent procedure and document the decision to participate in the IC (only V1 can be performed up to three days before vaccination);
b. Obtain demographic data, weight, height, waist circumference and determine pre-existing medical conditions and use of concomitant medications (only V1 can be performed up to three days before vaccination);
c. Obtain vital signs and conduct physical exam;
d. Check clinical inclusion and exclusion criteria;
e. Collect blood samples (additional samples will be needed for the first 60 participants of each age group at the first research center);
f. Collect sample for laboratory detection of SARS-CoV-2 (only V1);
g. Perform vaccination (see section 3.5.4);
h. Provide the participant with the “Participant's Diary” and one (1) thermometer;
i. Guide the participant about the technique for measuring their body temperature and measuring the temperature;
j. Observe the participant for 60 minutes after vaccination [34] and perform an assessment of the solicited adverse events;
k. Guide on searching the research center in case of fever or other symptoms of COVID-19;
l. Participants of childbearing potential must undergo the following procedures before vaccination:

m. Urine test for detection of β-hCG;
n. Review of the risk of pregnancy (see section 3.4.3);

3.5.3.2 Safety and Immune Response Visits (SI1 and SI2)

The intent of safety and immune response visits (SI1 and SI2) is to assess the occurrence of adverse reactions and collect samples to assess the immune response. These views should be scheduled at Week two and four after the second vaccination, with a window of up to one week after the visit, SI1: V2 + 2 weeks (+ 1 week) and SI2: V2 + 4 weeks (+ 1 week).

Procedures:

a. Update contact information, if necessary;
b. Obtain vital signs and anamnesis, with special attention to any clinical complaint, and physical examination to look for solicited and unsolicited adverse events;
c. Review and collection of the “Participant's Diary” (see section 3.5.5);
d. Collect blood sample (additional samples will be needed for the first 60 participants of each age group at the first research center);
e. Reinforce the importance of contacting the study team in case of fever or other symptoms of COVID-19;

For participants with the potential to become pregnant:

f. Urine test for detection of β-hCG (only SI1);

3.5.3.3 Immune Response Visits (I1, I2, I3 and I4)

The purpose of these visits is to collect and verify immune markers. Visit I1 is scheduled for 13 weeks (+1 week) after V1; Visit I2 for 26 weeks (+2 weeks) after V1; Visit I3 for 39 weeks (+3 weeks) after V1; and, Visit I4 for 52 weeks (+4 weeks) after view V1.

Procedures:

a. Update of contact information;
b. Perform anamnesis;
c. Collect blood sample (additional samples will be needed for the first 60 participants of each age group at the first research center):
d. Reinforce the importance of contacting the study team in case of symptoms of COVID-19;

3.5.3.4 Follow-up Contacts (C)

The purpose of the follow-up contacts (C) is to verify the occurrence of adverse events and cases of COVID-19 among the participants. These contacts may be made electronically, by telephone or in person, at the discretion of the study team and the participant who will inform the team about the contact forms they prefer. Electronic and telephone means include, but are not limited to, sending text or audio messages by telephone or internet through a computer program, application for tablet or smart phone (smartphone). Contact can also be made by home visit or the research center, if necessary.

Contacts will be made between the third and fifth day after each vaccination and thereafter every week for the first 13 weeks after vaccination and every two weeks for the remainder of the study. You will not need to make contact in the weeks when a visit to the research center is scheduled.
Any contact made, or attempted contact with the participant, must be registered in the Individual Participant Register.

Procedures:

a. Update of contact information;
b. Actively check for febrile events and suspected cases of COVID-19;

c. Check for unsolicited adverse events;
d. Reinforce the importance of contacting the study team in case of fever or other symptoms of COVID-19;

3.5.4 VACCINATION

The study doctor will authorize the inclusion of the participant on the day of vaccination. The unblinded pharmacist or nurse will request random allocation through the electronic system, as described in section 3.10.2. The pharmacist or nurse must separate and prepare to deliver the investigational product corresponding to the allocation. Unblinded personnel should not have contact with study participants or access the participant's identification data. It is recommended to apply each dose of the vaccine in a different arm.

3.5.5 FOLLOW-UP OF ADVERSE EVENTS IN THE TWO WEEKS AFTER VACCINATION AND USE OF THE "PARTICIPANT'S DIARY"

Participants will be instructed to complete a total of three "Participant Diaries" of fourteen days each to cover the period of up to four weeks after each vaccination. These diaries will contain relevant information about the study and in it the participants must record their body temperature, daily, for 14 days from each vaccination and any malaise or symptom, as well as medications used. On the 14th day after the second vaccination, the participant will receive the diary to fill in the events from the 15th to the 28th day after the second vaccination. In summary, participants must complete three 14-day diaries, one after the first vaccination and the other two diaries to cover the 28 days after the second vaccination.

The study team will provide 1 (one) thermometer to the participants and instruct them on the proper technique for the axillary measurement of their temperature and request that they be performed every day at approximately the same time. Participants will also be instructed to check their axillary temperature if they think they have a fever. In the case of a measurement compatible with fever (temperature above 37.8ºC*), it must be confirmed by a new measurement after an interval of 20 minutes.

Two weeks after vaccination, the study team will review the temperature records and other information written in the "Participant's Diary" to assess the occurrence of fever and adverse events (as described in section 3.5.5). The doctor will make a clinical assessment of potential adverse events and describe in the participant's individual record the events that occurred in the period considering severity and causal relationship with the product under investigation. It will also record in the individual record of the research participant the highest temperature recorded in the interval between the last visit and the current visit. The "Participant Diary" will be collected by the study team on this visit.
*** The temperature measured with a skin thermometer using a temporal scanner is considered equivalent to the axillary temperature.*

3.5.6 PROCEDURES IN THE EVENT OF FEVER AND SUSPICION OF COVID-19

The participant should be constantly advised to seek out the study team whenever he or she has a fever or other symptoms related to COVID-19, to assess whether it is a possible case (see section 3.16.7). The study team is responsible for establishing a routine that allows this participant to be evaluated as soon as possible as soon as he contacts the team.

In all possible cases, clinical samples for the detection of SARS-CoV-2 and a blood sample for serology (8.5 mL) will always be collected. In addition, a study physician must perform and record the participant's clinical evaluation. It should be noted that this assessment can be made from the second day of symptoms. The clinical presentation of COVID-19 can vary widely and can occur concurrently with other diseases,

which justifies the recommendation to collect laboratory samples to check COVID-19 in all possible cases, so that the diagnosis of COVID-19. All possible cases must be followed up to the resolution of all symptoms and the duration and severity of each of the signs and symptoms must be documented. TABLE 6 and TABLE 7 can be used to classify the severity of these signs and symptoms (see section 3.16.3). Every case must be monitored to check the severity of the condition according to the scale of clinical progression proposed in Table 9. Particular attention should be paid to the early detection of alarm signs and symptoms associated with severe COVID-19 in order to offer treatment in a timely and decrease the severity of clinical complications. Clinical case management will follow the guidelines of local health authorities according to clinical severity. Hospitalized cases must be monitored daily to verify their evolution according to the scale of clinical progression. Outpatient cases will only have the maximum severity and duration of symptoms recorded.

Local health authorities can request sample collection for the diagnosis of COVID-19. In this case, another battery of samples will be collected, different from the samples needed for the study. If another battery of samples is collected to meet local legal requirements, the results of these tests should be noted if the participant agrees to provide this information. An aliquot of samples collected for the study may also be provided for local analysis. In any case, the results of the study will be available for participants to take preventive action and report this data to local health authorities.

The performance of the tests of participants with a possible case of COVID-19 varies according to the evaluation day after the onset of symptoms. If the suspicion persists after obtaining a negative test result, a new clinical sample should be collected with an interval of at least two days. Oxygen saturation will be measured in all cases to complement the clinical evaluation. A blood sample (8.5 mL) should also be collected to assess serological response parameters.

3.5.7 PROCEDURE IN THE EVENT OF LOSS OF VISIT OR CONTACT

If the volunteer does not attend any of the scheduled visits or it is not possible to establish the scheduled contact within the time set for the window, the research team will continue to try to establish contact with the participant to check if there were any adverse events that prevented the participant from going to visit or contact the contact and check if there is any case of fever. When contact is reestablished with the volunteer who missed a scheduled visit, an extra visit can be scheduled by collecting samples corresponding to the closest visit. After this visit, the follow-up will return to the routine established in the study schedule. Attempts to contact must be documented and must be carried out at least four weeks apart. Study teams will cease contact attempts in the absence of any effective contact after a 13-week trial period.

In case of suspected adverse event, when contact is resumed, the participant may be summoned for evaluation on an extra visit. If there is no safety concern, the participant can be assessed on the next scheduled visit. Reports of adverse events to the Ethics Committee may be presented on a monthly basis, in a consolidated manner and following the guidelines of each Committee.

3.6 STUDY PROCEDURES DIAGRAM

The period of stay in the study for each participant will be approximately one year after vaccination, which will be considered as Day 0 of the study (visit V1).

During the study, face-to-face visits and contacts are provided for the monitoring of each participant (Table 4).

TABLE 4. DIAGRAM OF STUDY PROCEDURES

**Type of Visit**	**Enrolment, Vaccination 1**	**Vaccination 2**	**Safety and immunity**	**Monitoring of COVID-19**				
				**Safety and immunity**	**Immunity**			
**Visit**	**V1**	**V2**	**SI1**	**SI2**	**I1**	**I2**	**I3**	**I4**
Visit Schedule	0	V1 + 2 w (+2 w)	V2 + 2 w (+1w)	V2 + 4 w (+1 w)	V1+ 13 w (+2 w)	V1 +26 w (+ 2 w)	V1 + 39w (+ 3 w)	V1 + 52w (+ 4 w)
**Procedures**								
Informed Consent*	X							
Review of inclusion and exclusion criteria	X	X						
Medical History*	X	X						
Physical examination	X	X		X				
Vital signs	X	X	X	X				
Weight, height and waist circumference*	X							
Review of risk of pregnancy **	X	X						
Anamnesis of solicited adverse events	X	X		X				
Update contact information		X	X	X	X	X	X	X
Monitoring of COVID-19 and unsolicited adverse reactions		X	X	X	X	X	X	X
**Intervention**								
Vaccination	X	X						
**Diaries**								
Handing of diaries and thermometers	X	X	X					
Instruct how to measure the temperature every day	X	X	X	X				
Review of diaries		X	X	X				
**Safety assessment**								
β-hCG in urine **	X	X	X	X				
**Assessment for SARS-CoV-2**								
Test for detection of SARS-CoV-2 nucleic acid	X							
Qualitative test to detect anti-SARS-CoV-2 antibodies by chemiluminescence	X							
Samples for immunity testing	X	X	X	X	X	X	X	X
**Volume of blood collected (mL)**								X
All participants	**17**	**8.5**	**8.5**	**8.5**	**8.5**	**8.5**	**8.5**	**8.5**

Type of Visit	Enrolment, Vaccination 1	Vaccination 2	Safety and immunity	Monitoring of COVID-19				
				Safety and immunity	Immunity			
**Visit**	**V1**	**V2**	**SI1**	**SI2**	**I1**	**I2**	**I3**	**I4**
Visit Schedule	0	V1 + 2 w (+2 w)	V2 + 2 w (+1w)	V2 + 4 w (+1 w)	V1+ 13 w (+2 w)	V1 +26 w (+ 2 w)	V1 + 39w (+ 3 w)	V1 + 52w (+ 4 w)
**Procedures**								
Additional samples for the first 60 participants of each group of the first site	**51**	**42.5**	**42.5**	**42.5**	**42.5**	**42.5**	**42.5**	**42.5**

* Can be performed up to three days before the first vaccination
** For women of childbearing potential.

3.7 CRITERIA AND PROCEDURES FOR PARTICIPANT SUSPENSION OR EXCLUSION

At any time, if any participant wishes to leave the study or if the study doctor considers it necessary for a participant to be excluded, the exclusion visit will include the following procedures:

- If the exclusion occurs within the second week after vaccination, carry out the SI1 visit procedures;
- If the exclusion occurs after the SI1 visit, carry out the procedures for the next visit;

A previously excluded participant can resume the study procedures if the cause that justified their exclusion is resolved (e.g., participant who returns to the city after temporary change). If the cause of the exclusion was the participant's own desire, that participant can only resume the study procedures after a new documented informed consent procedure through the signing of a new Informed Consent Form.

If a study participant is unable to receive the second dose of the vaccine for any reason, he / she will be invited to remain in the study for the following visits and for surveillance of COVID-19 cases. In this case, the SI1 visit will be scheduled 4 weeks (+1 wk) after V1 and the SI2 visit will take place 6 weeks (+1 wk) after V1.

3.8 SAMPLE SIZE

3.8.1 SAMPLE SIZE CALCULATION AND STATISTICAL POWER

3.8.1.1 Efficacy

The sample size selected aims to accumulate sufficient number of cases for analysis of efficacy. The null (H0) and alternative (H1) hypotheses for the primary analysis of time to confirmed cases of COVID-19 in the vaccine and placebo groups are as follows:

> H0: The efficacy of the adsorbed vaccine COVID-19 (inactivated) produced by Sinovac against confirmed cases of COVID-19 has a lower limit of the 95% confidence interval less than or equal to 30%.
>
> H1: The efficacy of the adsorbed vaccine COVID-19 (inactivated) produced by Sinovac against confirmed cases of COVID-19 has a lower limit of the 95% confidence interval greater than 30%.

This study is designed as group sequential, providing an opportunity for unblinded analyzes of efficacy and futility review by the Data Safety Monitoring Committee (DSMC) when 40% of the total number of

cases is reached. Before performing the interim unblinded analysis, the DSMC will blindly review the accumulated case rate against the attack rate assumed in the calculations and may recommend an increase in the sample size under the premise that insufficient case accrual is due to a rate of attack less than expected.

The following assumptions and limits were used to obtain the sample size:

Attack rate over 12 months	2.50%
Vaccine efficacy (VE)	60.00%
Minimum VE to be demonstrated	30.00%
One-sided alpha	2.50%
Power	90.00%
Annual drop-out rate	5.00%
Number of interim analyses	100.00%
Spending function upper (efficacy) bound	Hwang-Shih-DeCani parameter, = -4
Spending function lower (futility) bound	Hwang-Shih-DeCani parameter, = -4
Recruitment of participants	uniform for the duration of enrolment
Allocation ratio	1: 1
Desired time for primary analysis	12 months

This design yields the following characteristics:

Number of events to trigger interim analysis	61
Expected time for interim analysis	~ 7 months
Number of events to trigger the primary analysis	151
P-value to declare efficacy in the interim analysis	0.0018
P-value for declaring efficacy in primary analysis	0.0241
Probability, under the alternative hypothesis, of exceeding the limit of efficacy in the interim analysis	0.2007
Cumulative probability, under the alternative hypothesis, of exceeding the limit of efficacy in primary analysis	0.9
Probability, under the alternative hypothesis, of exceeding the limit of futility in the interim analysis	0.0074
Cumulative probability, under the alternative hypothesis, of exceeding the limit of futility in primary analysis	0.1
Probability, under the null hypothesis, of exceeding the efficacy limit in the interim analysis	0.0018
Cumulative probability, under the null hypothesis, of exceeding the limit of efficacy in the primary analysis	0.0248
Probability, under the null hypothesis, of exceeding the limit of futility in the interim analysis	0.3543

Cumulative probability, under the null hypothesis, of exceeding the limit of futility in the primary analysis	0.9752

The primary analysis must be conducted after the last participant included completes a follow-up period of 12 months starting two weeks after the second dose. The design requires approximately 13,060 participants to be included. It is possible that the interim analysis will take place around the time when recruitment is being completed, providing the DMSC with an opportunity to suggest a blind adjustment of the sample size, just before proceeding to the interim unblinded analysis.

If in the final analysis, the success criterion is reached (p <0.0241), the estimated hazard ratio is expected to be approximately 50%, providing an estimated vaccine efficacy of approximately 50%, with an adjusted lower limit of the confidence interval above 30%.

3.8.1.2 Safety

The sample size calculation for safety was based on the denominator needed to determine the maximum risk of an adverse vaccine reaction occurring if the reaction is not reported in the study as described by Hanley and Lippman-Hand [35] .

Formula 1:

$$N_i = \frac{ln\,\alpha}{\ln(1-M)}$$

Where: Ni is the sample size of vaccinees for each age group, N1 = Adults (18-59 years) and N2 = Elderly (60 years or more) and α is the type I error

For this calculation, the following parameters were used in the age group of Adults (18-59 years) =

N1:

Type I error equal to 5% (two-tailed)

Maximum risk of 1 in 1000, equal to 0.001

Substituting these values in Formula 1, we obtain N1≅2,994

Following the 1:1 vaccine: placebo ratio, 2,994 participants will be on the placebo arm in this age group, out of a total of Adults N1 = 5,988. The minimum sample size of Adults by 5% to predict loss of follow-up 5,998 / 0.95≅6304 as the minimum number of adults (18-59 years) in the study.

In the Elderly age group (60 years or more) = N2, the following parameters were used:

Type I error equal to 5% (two-tailed)

Maximum risk of 1 in 500, equal to 0.002

Substituting these values in Formula 1, we obtain N2≅598

Following the 1: 1 vaccine: placebo ratio, 598 participants will be on the placebo arm in this age group, out of a total of Elderly N2 = 1,196. This sample size is corrected to predict the loss to follow-up of 5%,

yielding 1,196 / 0.95≅1,260, this being the minimum number of participants needed for this age group in the study, of which 630 will receive the study vaccine and 630 will receive the placebo.

3.8.2 ESTIMATED NUMBER OF PARTICIPANTS

The total number of participants needed to evaluate efficacy, 13,060 participants, satisfies the needed sample size calculated to evaluate safety (7,184). Therefore, the total number obtained for efficacy will be the number retained for the study. Up to 13,060 participants are expected to enter the study, with up to 11,800 participants aged 18 to 59 years and 1,260 elderly participants aged 60 and over. The recruitment of participants may be modified as recommended by the Data Safety Monitoring Committee at time of the interim unblinded analysis or blind assessment of the COVID-19 attack rate during the study.

3.9 POPULATION SOURCE OF STUDY AND RECRUITMENT AREA

The study population will be recruited from health professionals who work in areas specialized in the treatment of COVID-19, whether or not they belong to the institution where the research center is located. Interested persons will be able to contact the study center personally, through a phone call, electronic message, or with the contact form available on the study's website.

The study will be disseminated through printed advertising distributed or fixed in areas specialized in the treatment of COVID-19 or through the use of electronic account lists or contact data of the health units themselves. The Butantan Institute will maintain a website with contact information for all centers participating in the survey to inform anyone who wishes to participate. All materials for advertising the study will be forwarded for prior approval by the Research Ethics Committee.

There is a possibility that employees or students from health or educational institutions linked to research centers may be interested in participating in the study. In those cases, the study team will check whether the potential participant has any dependency relationship with the principal investigator or with the discipline to which the principal investigator belongs. In such cases, the right to refuse without any penalties is guaranteed.

3.10 PARTICIPANT ALLOCATION

3.10.1 ALLOCATION SEQUENCE GENERATION

There will be two randomization lists, one for each age group, based on the investigational products to be administered, i.e., vaccine or placebo at a 1: 1 ratio. Each randomization list will be made to include up to 11,800 18-59 year old adult and 1,260 elderly (60 and older) participants, the maximum number of participants needed per age group as described in Section 3.8.2.

3.10.2 ALLOCATION BLINDING MECHANISM

An electronic central randomization system will be used to designate the investigational product that each participant must receive.

A unblinded, qualified member of the study team (nurse / pharmacist) will obtain the corresponding randomization, separate the respective investigational product, blind the product and deliver it to the blinded team. The investigational product will be in a syringe that is in a blister labeled with the name of

the sponsor, code of the investigational product, route of administration, dose and expiration date. The unblinded study team members will not have contact with the participants, will not have access to identification data or any other involvement in the study, besides randomizing the participant, separating the syringe containing placebo or vaccine, checking if the information on the blister label and the cartridge label correspond and that the syringe is labeled with the clinical trial code, ID, corresponding visit and name of the Investigator. The opacity of the label makes the differences between the investigational products inconspicuous, and thus, the blinding is maintained.

3.10.3 ALLOCATION SEQUENCE GENERATION

An independent statistician, with no other involvement in the study, will generate the randomization sequences through a computer program that will feed the electronic central randomization system. The electronic central randomization system is a system validated in accordance with regulatory standards. This system will inform the unblinded team member which investigational product will be destined for each participant, and the order of designation of the IDs will follow the order of sequence of randomization and the stratum by age group.

3.11 BLINDING PROCEDURES

3.11.1 DESCRIPTION OF WHO WILL BE BLINDED IN THE STUDY

This trial is designed as a double-blind study to avoid introducing bias in the evaluation of efficacy, safety and immunogenicity. The clinical care team, the professionals responsible for the vaccination and the participants will not know which investigational product will be administered. Only pharmacists or nurses in the study who are responsible for the randomization, separation and blinding of the investigational product will have access to unblinded information. The allocation of the product under study will be revealed only after the completion of the monitoring of the participants and closing of the database to guarantee the unbiased evaluation of the product in the long term. The sponsor's operational team will also remain blind.

The individual results of the experimental immunology and virology tests will be released to the research centers only after the opening of the blind with written authorization from the sponsor. If necessary, independent scientists not involved with the clinical or laboratory evaluation of the participants, will be able to analyze unblinded data of laboratory results.

3.11.2 PROCEDURES FOR BREAKING THE BLIND

Early breaking of a participant's blind, if necessary, may be requested of the sponsor by the principal investigator, or his/her medical representative, and must occur in writing. The request may be motivated by emergency medical issues and / or legal and / or regulatory requirements. The principal investigator must document in writing the reasons for breaking the blind and inform them to the sponsor within two business days of the event. The blind will be broken through a specific form sent by the sponsor and will be restricted only to the participant for whom the application was completed. The early blind break should also be reported in the individual record of the research participant. The sponsor must report all early blind breaks to the Chairman of the Data and Safety Monitoring Committee (described in section 3.15.1) within two business days.

The Data and Safety Monitoring Committee can break the blinded code to assess the safety data of one or more participants. The justification for breaking the blinding code must be recorded in the corresponding meeting minutes. The code that is broken by the Committee will only be revealed to the sponsor and the investigator if it is relevant to the protection of one or more participants in the research.

3.12 DATA COLLECTION

Each study participant will have an individual research record that will be used as a source document. In this record, data from medical and nursing consultations should be recorded, such as: blood sample collections, laboratory results, among other documents related to the study. All source documents must be kept in the research center securely in order to maintain the confidentiality of the participants.

The “Participant's Diary”, described in section 3.5.5, will be considered a source document only for recording temperatures. Adverse events and the use of drugs recorded in the “Diary” must be reported in the individual record of the research participant by a study doctor.

All information relevant to the data analysis will be collected on Case Report Forms (CRF). Identifying information such as name, identity document, address, telephone number, among others, will not be recorded in any CRF. The identification of the participants in the CRF will only be through the individual ID generated by the system. This study will use an electronic data capture system to record information on CRFs. Data entry should be done only by authorized members of the study team using an individual password with an electronic signature. Electronic CRFs will be stored on a secure server designated by the sponsor.

3.13 DATA ANALYSIS AND PROCESSING PROGRAM

The data will be recorded directly and exclusively by the electronic CRF research center in the OpenClinica® Enterprise program (OpenClinica, Waltham, MA, United States), installed locally on the sponsor's server, validated and used by several American and European institutions. The database for statistical analysis will be extracted directly from the OpenClinica® Enterprise program. The main statistical analysis program for the study will be R, although other statistical programs can be used.

3.14 DATA ANALYSIS PLAN, INCLUDING STATISTICAL METHODOLOGY

3.14.1 DEFINITIONS OF POPULATION TO BE ANALYZED

To meet the objectives of the study, different data sets will be evaluated according to the nature of the endpoint.

Included population

Included population is defined as all screened participants who signed an informed consent form and are eligible to participate in the study, regardless of whether they were effectively randomized or not.

Safety population

The safety population is defined as all included participants who received at least one dose of the study vaccine and have safety data available. In case of allocation error, the participant will be analyzed according to the product he / she has actually received.

Reactogenicity Population

The population for the evaluation of reactogenicity is defined as all participants in the safety population who received and returned a participant's Diary to assess requested adverse events. The population is defined for each vaccination (first or second dose) and requires that the participant has actually received the corresponding dose.

Per-Protocol Population (PP)

The Per Protocol population is defined as all randomized participants who: met the inclusion criteria (Section 3.2.1) and did not meet any exclusion criteria (Section 3.2.2); received the two doses of the product under investigation, to which they were allocated, under the conditions of handling and administration recommended by the manufacturer (Section 3.4.1.1.2) and did not use restricted medications as required by the protocol (Section 3.4.2).

Participants will remain in the Per Protocol Population unless a major deviation occurs (e.g., receiving another vaccine for COVID-19) and these have contributed to the follow-up time for protocol analyzes until the exclusion event. A report will detail the excluded participants, the moment of exclusion and the justification. If the participant has no relevant protocol deviation, he will contribute until the end of his follow-up. Per Protocol analysis is the main analysis of the study's efficacy.

Intent to Treat Population

The Intent to Treat population will include all randomized participants who receive at least one dose of the product. Participants will be analyzed in the group to which they were allocated and contributed with the follow-up time that is available for the Intention to Treat analysis. Intention to Treat analysis is considered a secondary analysis of efficacy for this study.

Populations for immunogenicity

The populations for immunogenicity correspond to all participants of the population Per Protocol that have the corresponding samples to carry out the corresponding analyzes.

For the analysis of cellular immunity, the population is restricted to the first 60 participants of each age group in the first research center. For the analysis of humoral immunity and protection correlates, all participants who have available samples can contribute.

3.14.2 DESCRIPTIVE METHOOLOGY

All collected data will be summarized and / or listed. Descriptive statistics include mean, standard deviation, median and minimum and maximum values for continuous variables; and numbers and proportions by group for categorical variables. Unless otherwise specified, a two-tailed significance value of 5% will be used for statistical tests and confidence intervals. The exact confidence intervals will be used for univariate summaries of dichotomous variables, and score-based confidence intervals for differences between rates. All proportions will have as denominator the number of participants contributed with data within the specified time within the specified group and the specified study population. Abstracts will be presented by group and by time, when relevant.

Baseline demographic, anthropometric and previous exposure to SARS-CoV2 data will be presented descriptively. Regarding race or ethnic origin, the participant's self-declaration will be considered.

Analyzes can be performed according to age group and group, using Fisher's exact test for binary variables and analysis of variance (ANOVA) for continuous variables.

To list and summarize medications used by participants, the WHO Medication Dictionary tabulated by ATC classification, treatment group and preferred name will be used. Medical history and adverse reactions will be coded with MedDRA and tabulated by Class of organ and system (SOC), preferred term (PT) and allocation group.

The distribution of participants including the numbers of people included, screened, randomized and vaccinated will be summarized and presented in the CONSORT diagram, including discontinuation of the study. The causes of failed screening and discontinuation will be described.

3.14.3 PRIMARY EFFICACY ANALYSIS

The main analysis of efficacy will be a protocol-modified analysis calculated with all virologically confirmed cases of COVID-19 occurring in the period beginning two weeks after the second vaccination.

Vaccine efficacy among participants with symptoms of COVID-19 with detection of SARS-CoV-2 nucleic acid in a clinical sample will be assessed using Cox proportional hazards regression, stratified by age and sex, with an allocation group covariate to compare those who received vaccine with those who received placebo. This model calculates the estimated vaccine efficacy (1 - hazard ratio), 95% confidence interval and p values. Cumulative incidence charts will also be created with this model. This analysis will be performed in the population by protocol, as a primary analysis. In the population for Intent to Treat it will be considered as a secondary analysis

The hypothesis test of the primary efficacy endpoint in the Per Protocol population will be based on the alpha levels to be spent on each analysis and followed up with the corresponding confidence intervals. If additional events are accumulated after the final event necessary for the primary analysis, but before the database is closed, a final analysis will be carried out to incorporate these events.

Secondary efficacy endpoints will be assessed in the same way as the primary endpoint, providing for adjustments to the sequential assessment. The evaluation of the efficacy endpoint by stratum of randomization (age group) will be done in a similar way.

For each efficacy endpoint, the cumulative vaccine efficacy per time, defined as 100% × (1- cumulative incidence ratio per time unit), will be plotted with the time evaluation points and simultaneous 95% confidence interval calculation with the method of Parzen, Wei and Ying.

3.14.4 SAFETY ANALYSIS

The safety analyzes will include all participants who received the investigational product according to the product received, by group and age group. The primary safety analysis will consider all adverse events, solicited and unsolicited, with a reasonable causal relationship with the product that occurred in the first week after its administration according to the age group. The secondary analysis will extend this period until the fourth week after vaccination. In addition, unsolicited adverse events with a reasonable causal relationship with the product will be included in subsequent analyzes after the fourth week after administration of the product until the end of follow-up at week 52. The frequency and severity of adverse events and reactions will be reported for each dose investigational product.

All adverse events will be coded and grouped according to the MedDRA (Medical Dictionary for Regulatory Activities) methodology.

The solicited adverse events will be presented as the proportion of participants with observation of any event by group, and according to the type and severity grade of event in that group. The set of participants analyzed will correspond to the set of participants from which data exist, e.g., for immediate observation after vaccination it must be confirmed that the participant was observed during the 60 minutes after vaccination. The population for reactogenicity assessment will be analyzed for the events occurring during the first and until the fourth week post-vaccination, according to the availability of the Participant's Diary. The solicited adverse events recorded will be presented in a summarized form according to their maximum severity and duration per participant, when relevant. The rates will be accompanied by exact two-tailed 95% confidence intervals. Between groups, rates will be compared using two-tailed Fisher's exact test for paired comparisons, both in general and by type and severity.

Unsolicited adverse events analyzed will be those that occurred within 28 days after a vaccination, except when they are considered Serious Adverse Events or that have a reasonable causal relationship with the vaccination. Unsolicited adverse events will be summarized at the participant level where a participant contributes only once to a type of event given under maximum severity and / or causality. Additional tables can summarize the number of events of a certain type observed in a group, without considering the number of participants that originated it.

Unsolicited adverse events will be summarized by severity and by causal relationship with vaccination. In the case of Serious Adverse Events, criteria will also be recorded that meet the SAE classification. The tables will show the unsolicited adverse events with a frequency of 1% or more. Additional tables showed events that led to discontinuation of vaccination, which had severity 3 or 4 and serious adverse events. Among age groups, the rates of adverse events with a causal relationship to vaccination will be compared using two-tailed Fisher's exact test and Cochran-Mantel-Haenszel Test according to what is more appropriate.

The cases of COVID-19 and Severe Acute Respiratory Syndrome will be compared between the allocation groups both in frequency and severity. The severity score will be compared by the Wilcoxon test and the frequency of severe cases from the total of events will be compared to verify the difference in proportions between the groups. These comparisons are intended to assess whether there is any possibility of disease enhanced by the vaccine. A separate CRF will collect symptoms to determine respiratory disease and the severity of those symptoms for comparisons between groups as well as by age and sex.

Baseline data such as the presence of comorbidities will also be presented between groups. All cases of pregnancy will also be described, including endpoints.

3.14.5 IMMUNOGENICITY ANALYSIS

The analysis will describe the immunogenicity results of a subgroup of participants from each age group in terms of seroconversion of neutralizing antibodies. The geometric means of the titers will also be described among those with seroconversion and will be compared between those who acquired the infection and a subgroup of those without infection. This information will be elucidated in the light of the corresponding arm, whether the vaccine or the placebo. The analyzes considered the presence of antibody titers before the first vaccination and the documented report of previous infection.

The following descriptive statistics will be calculated for each assessment and each group:

• Seroconversion rates with 95% confidence intervals compared to baseline titer

• Median of titles with 95% confidence intervals

• Graphs of the cumulative reverse distribution of bonds / concentrations

• Box graphs of title / concentration distributions

3.14.6 MISSING / MISSING DATA MANAGEMENT

No data will be allocated for the primary analysis of efficacy per protocol. Therefore, the analyzes excluded participants with missing or unevaluable data. If there is an excessive amount of missing data or there is some kind of pattern between the missing data, additional statistical tools can be implemented.

3.15 STUDY DATA MONITORING

3.15.1 DATA MONITORING AND SAFETY COMMITTEE

This study will have a Data and Safety Monitoring Committee constituted according to the recommendations of the Brazilian Ministry of Health [36]. This committee will be appointed by the sponsor and will consist of three people with one of the following qualifications:

- Infectologist doctor: must have experience in carrying out studies related to the object of the study.
- Epidemiologist: must have experience in conducting studies or in epidemiological surveillance related to the object of the study.
- Statistician: Must have an undergraduate or graduate degree in statistics. Must have experience in data analysis in the health field.
- Other health professionals: they must have experience in the design, conduction or analysis of clinical trials.

The committee members will be completely independent from the sponsor. There is no provision for payment of fees for members of the committee. Sponsor employees will be able to support the committee's activities, but they will not be able to be part of it.

The committee will make written recommendations to continue, modify, suspend or terminate the study. These recommendations will be received by the sponsor and communicated to the researchers so that they can forward them to the respective Research Ethics Committees. The sponsor may disagree with the recommendations through a justified response that will be communicated in the same way.

3.15.2 ANALYSIS AND INTERIM REVIEW

The Data and Safety Monitoring Committee will have access to data on the safety of the participants, including the results of laboratory tests throughout the study and will be convened periodically for safety blinded analyses when:

- safety information for the two weeks after the first vaccination of the first 450 participants in the Adult group (18-59 years) is available;
- safety information for the two weeks after the first vaccination of the first 450 participants in the Elderly group (60 years or older) is available;

- safety information for the four weeks after the second vaccination of all participants in the Elderly group (60 years or older) is available;
- safety information of the two weeks after the first vaccination of the first 1350 participants in the Adults group (18 to 59 years old) is available;
- safety information for the four weeks after the second vaccination of all participants in the Adults group (18 to 59 years old) is available.

After the safety review meetings described above, other meetings will be agreed at least every six months, as decided by the committee chairman.

If the Independent Data and Safety Monitoring Committee deems it necessary, it may break the blind of any participant early during the study to assess safety.

After each meeting, the Independent Data and Safety Monitoring Committee must issue a report to the sponsor indicating the continuity or suspension of the administration of the investigational product, accompanied or not by additional recommendations for the protection of study participants. Interim analyzes of efficacy, safety and immunogenicity, without breaking the allocation code of individual participants, may be part of the Committee's reports. After all participants have received the last vaccination, the reports should contain the recommendations considered necessary to maintain the protection of the study participants.

The Independent Data and Safety Monitoring Committee may perform interim analyzes of efficacy and immunogenicity that are not blinded before the conclusion of the study when the following conditions are met:

- When safety data for the first week after the second vaccination of at least 6000 participants in the Adult group (18 to 59 years) are available;
- The number of virologically confirmed COVID-19 cases is above the stipulated in section 3.8.1.1, which corresponds to 61 cases.

If the interim analysis of efficacy demonstrates vaccine efficacy according to the statistical criteria in section 3.8.1.1, the results will be announced to the ethical and regulatory authorities and the vaccination schedule will be offered at visit I1 for participants who received a placebo. If the results show futility in accordance with the statistical criteria in section 3.8.1.1, recruitment of the study will be stopped if it is still in progress.

3.15.3 CRITERIA FOR SUSPENSION OR INTERRUPTION OF THE STUDY

If the vaccine's reactogenicity is found to be unacceptable, or if there is early evidence of very high or very low efficacy, its administration will be suspended until the Independent Data and Safety Monitoring Committee and the sponsor review the available data.

Administration of the investigational product will be resumed if the review conducted by the Independent Data and Safety Monitoring Committee and the sponsor results in such a recommendation. If the administration of the product is terminated at the time of the events described above, the Committee may review the data and make recommendations for monitoring the participants.

The reports with safety data and changes in the situation of the administration of the investigational product (suspended / resumed) will be submitted to the Research Ethics Committee in accordance with institutional policies.

These criteria are considered to be minimal and the decision to suspend the administration of the investigational product in any of the study centers may be taken, based on any other relevant criterion in the judgment of the investigator and / or the Research Ethics Committee. Likewise, regulatory authorities or Sponsor may suspend administration of the product if there is a major concern about the safety of the vaccine.

3.16 SAFETY ASSESSMENTS

3.16.1 DEFINITIONS

The definitions of adverse events follow the recommendations of Good Clinical Practice and the E2A Guide on the Management of Clinical Safety Data of the International Harmonization Conference (ICH) [37].

3.16.1.1 Adverse Event

In this study, an adverse event will be defined as any unfavorable medical occurrence that occurs in a participant who has been vaccinated and who does not necessarily have a causal relationship with the administration of the vaccine or placebo. Therefore, an adverse event can be any unfavorable and unintended sign, symptom or disease (including an abnormal finding in laboratory examination), temporarily associated with the vaccination product, whether or not it is considered to be related to the vaccination product.

3. 16.1.2 Serious Adverse Event

In this study, a Serious Adverse Event (SAE) will be defined as any adverse event that results in any of the following outcomes [38]:

- Death.
- Threat to life. There is a risk of death at the time of the event.
- Hospitalization or extension of hospitalization.
- Significant or persistent disability: a substantial disruption of a person's ability to conduct normal life functions.
- Congenital anomaly.
- Any suspicion of transmission of an infectious agent by means of a medication.
- Clinically significant event: any event resulting from the use of drugs that require medical intervention, in order to avoid death, risk to life, significant disability or hospitalization.

An adverse event needs to fulfill only one of the above criteria to be considered a Serious Adverse Event. The classification of an adverse event as a Serious Adverse Event does not depend on its causal relationship with the vaccination product.

3.16.1.3 Adverse reaction

In this study, adverse reactions that have a reasonable causal relationship with the product under investigation will be considered as adverse events.

3.16.1.4 Pre-existing conditions / Worsening of pre-existing conditions

Stable medical conditions in effect at the participant's inclusion visit should be reported in the individual record of the research participant and will not be considered adverse events. The worsening of a pre-existing condition will be defined as an adverse event and should be assessed as such. An adverse event

resulting from the worsening of a pre-existing condition will be considered as resolved when the pre-existing condition returns to its baseline condition recorded at the inclusion visit.

3.16.2 MONITORING ADVERSE EVENTS

The principal investigator or his / her medical representative should include in his / her assessment of adverse events:

- Whether it is a serious adverse event (see section 3.16.1.2);
- The classification of its severity grade (see section 3.16.3);
- The classification of its causal association with the investigational product (see section 3.16.4);
- The measures taken to manage the case;
- Its evolution.

After vaccination, all participants will be asked about the occurrence of specific signs and symptoms (solicited adverse events) and also about the occurrence of additional signs and symptoms spontaneously reported (unsolicited adverse events).

3.16.3 SEVERITY GRADING

The severity of solicited adverse events will be classified using a numerical scale from 1 to 4, according to TABLE 5 (local reactions) and TABLE 6 (systemic reactions), created based on the guides "Toxicity Grading Scale for Healthy Adult and Adolescent Volunteers Enrolled in Preventive Vaccine Clinical Trials "from the United States Food and Drug Administration (FDA) and in the" Common Terminology Criteria for Adverse Events - Version 5.0 "guide by the United States National Cancer Institute (NCI / NIH).

TABLE 5 CLASSIFICATION OF THE SEVERITY OF THE SOLICITED LOCAL CLINICAL ADVERSE REACTIONS

**Adverse Event**	**Grade 1**	**Grade 2**	**Grade 3**	**Grade 4**
Pain at the site of the investigational product administration	Does not interfere with daily activities	Repeated use of non-narcotic pain reliever > 24 hours OR interferes with activity	Any use of narcotic pain reliever or prevents daily activity	Visit to the emergency room* OR Hospitalization
Erythema at the site of investigational product administration†	25 – 50 mm	51 – 100 mm	> 100 mm	Necrosis OR Exfoliative dermatitis
Swelling at the site of investigational product administration†	25 – 50 mm	51 – 100 mm OR interferes with activity	> 100 mm OR prevents daily activity	Necrosis
Induration at the site of investigational product administration†	25 – 50 mm	51 – 100 mm OR interferes with activity	> 100 mm OR prevents daily activity	Necrosis

Adverse Event	Grade 1	Grade 2	Grade 3	Grade 4
Pruritus at the site of investigational product administration	Does not interfere with daily activities	Interferes with activity	Prevents daily activity	Visit to the emergency room* OR Hospitalization

* Requires 12 hours or more of admission to hospital ward or emergency room for adverse event management
† The recorded value must be the one measured at the site with the largest diameter and as a continuous variable.

TABLE 6 CLASSIFICATION OF SEVERITY OF THE SOLICITED SYSTEMIC CLINICAL ADVERSE EVENTS AND OF THE SIGNS AND SYMPTOMS IN CASE OF FEVER AND SUSPICION OF COVID-19

Adverse Event	Grade 1	Grade 2	Grade 3	Grade 4
Fever	37.8 – 38.4°C	38.5 – 38.9°C	39.0 – 40.0°C	>40°C
Nausea	Does not interfere with daily activities OR 1 to 2 episodes in 24 hours	Interferes slightly with daily activities OR More than 2 episodes in 24 hours	Prevents daily activities, requires intravenous hydration	Visit to the emergency room* OR Hospitalization for hypovolemic shock
Vomiting	Does not interfere with daily activities OR 1 to 2 episodes in 24 hours	Interferes slightly with daily activities OR More than 2 episodes in 24 hours	Prevents daily activities, requires intravenous hydration	Visit to the emergency room* OR Hospitalization OR Hypovolemic shock
Diarrhea	2 – 3 loose stools in 24 hours	4 – 5 stools in 24 hours	6 or more watery stools or requires outpatient IV hydration	Visit to the emergency room* OR Hospitalization
Headache	Does not interfere with daily activities	Repeated use of non-narcotic pain reliever > 24 hours OR Interferes slightly with daily activities	Significant; any use of narcotic pain reliever or prevents daily activity	Visit to the emergency room* OR Hospitalization
Fatigue	Does not interfere with daily activities	Some interference with activity	Significant; prevents daily activity	Visit to the emergency room* OR Hospitalization

Adverse Event	Grade 1	Grade 2	Grade 3	Grade 4
Myalgia	Does not interfere with daily activities	Some interference with activity	Significant; prevents daily activity	Visit to the emergency room* OR Hospitalization
Chills	Feeling slightly cold; chills, teeth chattering	Moderate whole body shivering, requires use of opioids	Severe or prolonged, does not respond to opioids	-----
Anorexia	Loss of appetite without alteration in eating habits	Oral intake altered without significant weight loss or malnutrition; oral nutritional supplements indicated	Associated with significant weight loss or malnutrition (e.g., inadequate oral caloric and/or fluid intake); tube feeding or Total parenteral nutrition indicated	-----
Cough	Mild symptoms; nonprescription intervention indicated	Moderate symptoms, medical intervention indicated; limiting instrumental activities of daily living	Severe symptoms; limiting self care activities of daily living	-----
Arthralgia	Mild pain	Moderate pain; limiting instrumental activities of daily living	Severe pain; limiting self care activities of daily living	-----
Pruritus	Mild or localized; topical intervention indicated	Widespread and intermittent; skin changes from scratching (e.g., edema, papulation, excoriations, lichenification, oozing/crusts); oral intervention indicated; limiting instrumental activities of daily living	Widespread and constant; limiting self care activities of daily living or sleep; systemic corticosteroid or immunosuppressive therapy indicated	-----
Skin rash (exanthema)†	Present, but asymptomatic	Symptomatic (pruritus/pain), but interferes only slightly with daily activities	Symptomatic, prevents daily activities	Visit to the emergency room* OR Hospitalization
Allergic reaction	Systemic intervention not indicated	Oral intervention indicated	Bronchospasm; hospitalization indicated for clinical sequelae; intravenous intervention indicated	Life-threatening consequences; urgent intervention indicated

The severity of unsolicited clinical adverse events will be classified using a numerical scale from 1 to 4, according to TABLE 5, created based on the "Toxicity Grading Scale for Healthy Adult and Adolescent Volunteers Enrolled in Preventive Vaccine Clinical Trials" guide of the Administration United States Food

and Drug Administration (FDA) and the "Common Terminology Criteria for Adverse Events - Version 5.0" guide of the United States National Cancer Institute (NIC / NIH) .

TABLE 7 CLASSIFICATION OF THE SEVERITY OF UNSOLICITED CLINICAL ADVERSE EVENTS AND OF OTHER SIGNS AND SYMPTOMS IN CASE OF FEVER AND SUSPICION OF COVID-19

Adverse Event	Grade 1	Grade 2	Grade 3	Grade 4
Respiratory rate	17-20 breaths per minute	21-25 breaths per minute	>25 breaths per minute	Intubation
Dyspnea	Shortness of breath with moderate exertion	Shortness of breath with minimal exertion; limiting instrumental activities of daily living	Shortness of breath at rest; limiting self care activities of daily living	Life-threatening consequences; urgent intervention indicated
Nasal congestion	Mild symptoms; intervention not indicated	Moderate symptoms; medical intervention indicated	Associated with bloody nasal discharge or epistaxis	
Anosmia	Present	-----	-----	-----
Dysgeusia / Ageusia	Altered taste but no change in diet	Altered taste with change in diet (e.g., oral supplements); noxious or unpleasant taste; loss of taste	-----	-----
Illness or clinical adverse event (as defined according to applicable regulations) and other signs and symptoms	No interference with activity	Some interference with activity not requiring medical intervention	Prevents daily activity and requires medical intervention	Visit to the emergency room* OR Hospitalization

3.16.4 CLASSIFICATION OF THE CAUSAL RELATIONSHIP

All adverse events should be classified by the principal investigator or his medical representative as to their causal relationship with the product under investigation, according to the classification adapted from the "Uppsala Monitoring Center" of the World Health Organization (WHO-UMC) [39], described in TABLE 8.

The sponsor may request additional clarifications from the principal investigator to justify the causal relationship attributed to the event. The causal relationship may also be reviewed at the request of the Independent Data and Safety Monitoring Committee upon written justification. All local reactions after administration of the product under investigation will be considered as adverse events with a certain causal relationship to vaccination.

TABLE 2 CLASSIFICATION OF CAUSAL RELATIONSHIP OF ADVERSE EVENTS WITH THE INVESTIGATIONAL PRODUCT

**Reasonable causal relationship**			**Causal relationship NOT reasonable**	
Adverse Event considered as Adverse Reaction			Adverse Event cannot be considered as Adverse Reaction.	
**Certain**	**Probable**	**Possible**	**Unlikely**	**Not related**
Event or change (abnormal value) in a laboratory test, with plausible temporal relationship with regarding the administration of the intervention;	A clinical event, including a change (abnormal value) in a laboratory test, with a reasonable temporal relationship regarding the administration of the intervention;	A clinical event, including a change (abnormal value) in a laboratory test, with a reasonable temporal relationship regarding the administration of the intervention;	A clinical event, including a change (abnormal value) in a laboratory test, which, due to the time of the administration of the intervention, gives cause to an unlikely, but not impossible, relationship;	A clinical event, including a change (abnormal value) in a laboratory test, which, due to the time of the administration of the intervention, gives cause to a non-existing relationship;
It cannot be explained by a concurrent disease or another intervention or medication;<br><br>The event is defined pharmacologically or phenomenologically (i.e., a specific and objective disorder or a pharmacologically recognized phenomenon);	It is unlikely to be caused by a concomitant illness or by another intervention or medication;	It can also be explained by concurrent disease or other interventions or medications;	Another disease or another drug provide a plausible explanation	Another disease or another drug provide a plausible explanation
The response to discontinuation or withdrawal is plausible (pharmacologically, pathologically);	The response to discontinuation or withdrawal is clinically reasonable;	Lack of information or lack of clarity about withdrawal or treatment discontinuation		
Re-exposure is satisfactory, if required	Re-exposure is not required			

The safety monitoring in this study will include clinical and laboratory assessments of the participants, covering both local and systemic adverse events, both solicited and unsolicited.

Initially, participants will be observed at the study center for 60 minutes after each vaccination and will be instructed to record any adverse reactions within the first three weeks after vaccination, with additional visits to be scheduled if necessary. Reasonably related adverse events are expected to occur more frequently in the first two weeks after vaccination.

On visit V2, on the day of the second vaccination, and on visits SI1 and SI2, two and four weeks after the second vaccination, the researchers responsible for the clinical and laboratory evaluation of the participants will check the records in the "Participant's Diary", will question the participants about the occurrence of possible adverse events and will perform a physical examination. Participants will be able to contact a study doctor 24 hours a day for the entire study period via a telephone number.

3.16.5.1 Reporting of Adverse Events

All adverse events that are identified during the study must be recorded in a source document. Adverse events that occurred in the first 28 days after vaccination and those that happened at any time that have a reasonable causal relationship with the product under investigation should be reported to the sponsor through the adverse event case report form (CRF), within seven days. knowledge of it by any member of the study team. Updates about an adverse event should be recorded as soon as new information is available until its outcome (recovered, in recovery, recovered with sequelae, loss of follow-up, not recovered or death).

3.16.5.2 Expediting Reporting of Events of Special Interest

The Expedited Reporting of Events of Special Interest aims to inform the sponsor, other investigators and regulatory bodies about new data regarding serious reactions or occurrences that require special monitoring. The Priority List of Adverse Events of Special Interest in COVID-19 vaccines prepared by Brighton Collaboration [40] is included in events of special interest. In this study, the following should be reported expeditiously:

- Serious Adverse Events (see section 3.16.1.2);
- Confirmed case of COVID-19, including serious cases (see sections 3.1.1.7.7 and 3.16.7.4);
- Pregnancy in the first 4 weeks after the last vaccination;
- Generalized seizure;
- Guillain-Barré syndrome;
- Acute disseminated encephalomyelitis;
- Hematological thrombocytopenia;
- Immune anaphylaxis;
- Vasculitis;
- Other serious local or systemic adverse events after immunization.

All Events of Special Interest must be reported to the Sponsor within one (1) calendar day from the date of knowledge of the event by the research center staff. The initial notification should not be extended even if the information is incomplete. The report of Serious Adverse Events will be filled out preferably through a specific form that is integrated into the CRF system after evaluation by a study doctor. If it is not possible to access the CRF, the notification must be made through a specific form sent by email or telephone call to the team of the Sponsor's Clinical Trials and Pharmacovigilance Division and will be registered as soon as possible in the form integrated in the above mentioned CRF system referencing contact date and time of the first notification by any means.

For Serious Adverse Events and probable or confirmed cases of COVID-19, updates should be sent every two weeks, or as soon as new information is available until its final outcome (recovered, in recovery, recovered with sequel, loss of follow-up, not recovered or death) and whether or not there was a need for hospitalization. For pregnancies in progress in the first weeks of study, updates should be sent periodically until their outcome (delivery, abortion or stillbirth) to ensure verification of the occurrence or not of Serious Adverse Events. Volunteers whose pregnancy has been detected in the first 13 weeks of study will be referred for follow-up at a specialized obstetrics service and followed up until the outcome of the pregnancy and up to 6 weeks after delivery, if necessary.

The CRF with information on Serious Adverse Events must be completed and updated in all cases with the information evaluated by a study physician, regardless of the form of initial notification of the event. When a Serious Adverse Event has its causal relationship with the product under investigation classified as unreasonable, an alternative etiology, diagnosis or explanation must be provided. In this study, the following types of hospitalizations do not generate an Urgent Report:

- Any admission unrelated to an adverse event (e.g., childbirth, cosmetic or scheduled surgery);
- Any admission for diagnosis or therapy of a clinical condition that existed prior to administration of the product under study investigation and which has not increased in frequency or severity, in the judgment of one of the study physicians;
- pregnancies where the day of conception is later than Day 28 post-immunization; is determined by a negative β-hCG test, in urine or blood, performed on Day 28 or later and prior to pregnancy, or by calculating gestational age performed during pregnancy monitoring.

3.16.6 SAFETY ASSESSMENT PERIOD

All solicited and unsolicited adverse events identified during the first four weeks (28 days) after vaccination should be reported regardless of their causal relationship with the products under investigation. Unsolicited adverse events with a causal relationship reasonable with the research product at the discretion of the investigator and / or the sponsor should be reported at any time during the study. Serious adverse events, regardless of their causal relationship, should be reported at any time during the study or even after the study.

Pregnancies where it is proven that conception occurred beyond four weeks after the last immunization will not be followed up to assess serious adverse events, given that conception would have occurred after clearance of the vaccine. COVID-19 cases will be reported for the duration of the study.

3.16.7 COVID-19 CASE DEFINITIONS

The definition of case caused by the 2019 SARS-CoV-2 (COVID-19) that will be used in this study will be that stated by the FDA guides [26], as follows:

Anyone who has at least one of the following symptoms for two days or more should be tested to detect SARS-CoV-2 nucleic acid in a clinical sample:

- Fever or chills;
- Cough;
- Shortness of breath or difficulty in breathing;
- Fatigue;
- Muscle or body pain;
- Headache;
- Loss of smell or new taste;
- Sore throat;
- nasal congestion or runny nose;
- Nausea or vomiting;
- Diarrhea.

The definition of symptoms is subjective and depends on what each participant considers abnormal for their routine condition. It is recommended to evaluate all participants between the second and the seventh day of presentation of these symptoms, although collection is allowed until the fourteenth day after the onset of symptoms.

Laboratory criteria
Detection of SARS-CoV-2 nucleic acid in a clinical sample.

Classification of cases

Possible:

Anyone who meets the clinical criteria.

Confirmed case:

Anyone who meets the laboratory criteria.

Discarded case:

A discarded case is a possible COVID-19 case that had two negative RT-PCR tests for detection of SARS-CoV-2 nucleic acid with at least two days between them. For the second test the sample collection period for nucleic acid detection must be repeated within 14 days after the onset of symptoms, preferably within seven days. If the second collection exceeds 14 days, a serological sample will be evaluated until the 28th day after the onset of symptoms to assess possible recent exposure.

COVID severe case:

Refers to a laboratory confirmed case of SARS-CoV-2 infection that has one or more of the following conditions [26]:

- Clinical signs at rest indicating severe systemic disease (respiratory rate ≥ 30 per minute, heart rate ≥ 125 per minute, oxygen saturation ≤ 93% at room temperature at sea level or PaO2 / FiO2 <300 mm Hg);
- Respiratory failure (defined as the need for high-flow supplemental oxygen, non-invasive ventilation, mechanical ventilation or extracorporeal oxygenation);
- Evidence of shock (Systolic BP <90 mm Hg, Diastolic BP <60 mm Hg, or need for vasopressors);
- Major acute renal, hepatic or neurological dysfunction;
- Admission to the Intensive Care Unit;
- Death.

Clinical progression scale for SARS-CoV-2 infection

All cases of SARS-CoV2 infection will be classified according to the scale of clinical progression proposed by the World Health Organization [41] according to Table 9. The evaluation of hospitalized cases (score 4 or higher) will be done daily until the resolution of symptoms. In non-hospitalized cases, the maximum score and duration of symptoms will be recorded (score 1-3).

Table 9. Scale of clinical progression of SARS-CoV-2 infection. Adapted from a proposal by the World Health Organization [41].

Score Description	
Uninfected, viral RNA not detected	0
Asymptomatic, viral RNA detected	1
Symptomatic, independent	2
Symptomatic, need help	3
Hospitalized *, without oxygen	4
Hospitalized, supplemental oxygen by mask or nasal cannula	5

Hospitalized, oxygen by non-invasive or high flow ventilation	6
Intubation and mechanical ventilation, P02 / FiO2 ≥150 or SpO2 / FiO2 ≥ 200	7
Mechanical ventilation P02 / FiO2 <150 (SpO2 / FiO2 <200) or vasopressors	8
Mechanical ventilation P02 / FiO2 <150 (SpO2 / FiO2 <200) and vasopressors, dialysis or extracorporeal oxygenation	9
Death	10

** If hospitalization is for isolation only, record the status with an outpatient*

3.16.8 RESPONSE TO NEW OR UNEXPECTED FINDINGS AND CHANGES IN THE STUDY ENVIRONMENT

All adverse events will be assessed by an Independent Data and Safety Monitoring Committee. If the number of adverse events is higher than expected (see section 3.15.3 of this protocol), the administration of the product under investigation may be suspended.
The principal investigator and / or the sponsor may suspend the administration of the product under investigation in order to protect participants, should a potential unforeseen risk be identified.
In the event of suspension of administration of the product under investigation, participants will continue with their safety assessments by adding new parameters that are necessary to prevent possible risks. The continuation or permanent interruption of the administration of the product under investigation will only happen after approval by the Research Ethics Committee.

3.17 DATA QUALITY ASSURANCE / CONTROL

3.17.1 CLINICAL MONITORING

Monitoring of this study will be conducted in accordance with the Americas Document for Good Clinical Practice and standard operating procedures in accordance with applicable government regulations.
The principal investigator will be informed about the frequency of monitoring visits, notified in advance before each visit and should be available, along with the rest of the research center staff, during all visits to clarify doubts and discussions about the monitoring of the study.

The objectives of the monitoring visits will be to verify the prompt communication of adverse events, the existence of the signed informed consent form for each participant, to compare CRFs and source documents to ascertain their completeness and accuracy, to ensure the protection of the study participants, to check the conduct of the study as per protocol, and to evaluate the completeness and accuracy of other records that are deemed necessary. Study documentation will be available for analysis, upon request, throughout the course of the study.

In addition to the monitoring visits, the monitors will monitor the entry of data in the electronic CRFs in order to detect any anomaly that requires attention before the following visits are carried out. Special emphasis will be placed on serious adverse events and information that could compromise the safety of the study participants. Considering the restrictions of health and biosafety authorities in areas where the research centers are located, the monitoring plan will contemplate the possibility of centralized and / or remote monitoring according to the circumstances of the research center and greater efficiency in the process within the guidelines of the ethical and regulatory authorities.

3.17.2 ACCESS TO SOURCE DOCUMENTS

Study-related documents must be kept by the principal investigator in a secure manner for a period of two years after the final approval of the marketing of the study vaccine, or until two years have elapsed since the formal interruption of the clinical development of the product. The maintenance time of these

documents must also be in accordance with the time required by the Research Ethics Committee and by ANVISA, and the longer time must prevail. It is the sponsor's obligation to inform the principal investigator, in writing, when it is no longer necessary to keep these documents.
No study document should be destroyed before the deadline established by legal precepts and the ethical and regulatory standards of Brazil without the prior written agreement between the sponsor, the Research Ethics Committee, the Independent Data and Safety Monitoring Committee and ANVISA .
No medical record from an external institution will be requested unless there is a need to clarify the participant's medical history and will only be requested after the participant's voluntary and informed consent in writing.

All source documents will be available upon request for the assessment of monitors and auditors appointed by the sponsor and for inspectors of competent regulatory authorities in accordance with current regulations and legal provisions.

4 ETHICS AND PUBLICATIONS

4.1 APPROVALS FOR CARRYING OUT THE RESEARCH

4.1.1 STATEMENT OF THE REGULATORY STUDY FRAMEWORK

The protocol is regulated by Resolutions 466/12 [42] and 441/2011 [43] of the National Health Council, the Declaration of Helsinki in its most recent version and the Guidelines on Good Clinical Practices of the International Harmonization Conference, RDC 9 / 2015 of ANVISA [44]. Any other applicable standard that may be incorporated into Brazilian regulations during the protocol will be applied to this study.

4.1.2 STUDY APPROVALS

Each principal investigator will conduct the study in accordance with the protocol agreed with the sponsor and approved by its Research Ethics Committee and by the National Health Surveillance Agency of Brazil - ANVISA. The original version of the protocol is in Portuguese.

4.2 AMENDMENTS TO THE PROTOCOL

If necessary, sponsor representatives and investigators may amend the protocol by mutual agreement. Amendments to the protocol must be submitted for approval by the respective Research Ethics Committees before their implementation. ANVISA will be notified of each amendment as soon as approval by the Ethics Committees is obtained under the terms of the current regulation. The study is registered in public databases that will be updated as soon as the amendment obtains all necessary approvals.

No investigator is authorized to make any changes to this protocol without obtaining authorization from the sponsor and the Research Ethics Committee except to eliminate immediate risk (s) to the participants. In case of an emergency to eliminate the risk of the participants, the amendment to inform the Ethics Committees and ANVISA will be sent as soon as possible.

4.3 VOLUNTARY INFORMED CONSENT

4.3.1 PROCEDURES FOR IMPLEMENTING AND DOCUMENTING VOLUNTARY INFORMED CONSENT

In order to obtain and document voluntary informed consent, the principal investigator and his team must comply with the applicable regulatory standards and requirements, Good Clinical Practices and ethical principles.

The Informed Consent Form must be approved by the Research Ethics Committee before its use. The participant will have the opportunity to read, ask questions and clarify their questions before confirming your intention to participate in the study and you will receive a copy of the signed informed consent form.

The participant may withdraw his consent at any time, even after the beginning of the study procedures, without prejudice to your attendance at the institution, if this attendance is due to your participation in the study. Participants' rights and well-being will be protected by emphasizing that the quality of their medical care will not be affected if they do not accept to participate in the study.

4.3.2 STORAGE OF BIOLOGICAL SAMPLES AND USE OF BIORREPOSITORIES

All biological material collected in the course of the study may, with the participant's permission, be kept for use in future research in a specific biorepository of the study. This material can be used to validate results and improve tests during the study and to establish longitudinal comparisons of the participants through the follow-up time.

Stored samples can also be used to obtain data for investigating adverse events when there is a medical indication. The retention period of the material will be until the completion of the processing of all samples of the study, except when mediating specific authorization for retention for a longer period to carry out sub-studies according to ethical standards. The participant may withdraw his permission at any time, in which case the material will be made available to him.

The extension of the storage period will be subject to ethical approval in accordance with current regulatory provisions. This biological material will not be commercialized or used for the production of commercial products. All biological material will be stored and will be identified with the identification number received by the participant after being included in the study. Samples may be exported to the laboratory where the vaccine was developed in China to validate results and improve tests. Brazilian researchers will have unrestricted access to exported samples and Brazilian standards on research on human beings and storage of samples will be respected, including with regard to the prohibition of commercialization or patent filing of discoveries in these samples.

The extensive characterization of the immunological response generated by the vaccine is essential for an adequate understanding of its safety and immunogenicity profile. The storage of blood samples from the participants included in this study will make it possible to carry out additional research projects that will assist in the improvement of this vaccine or in the future development of other COVID-19 vaccines.

Any new research project that is carried out in the future with the stored material will be submitted for approval by the Research Ethics Committee. All procedures for handling the biorepository will follow the guidelines of Resolution No. 441 of the National Health Council [43].

4.4 DESCRIPTION OF RISKS (PHYSICAL, SOCIAL AND PSYCHOLOGICAL) FOR THE INDIVIDUAL OR GROUP AND METHODS TO MINIMIZE THESE RISKS

In this study, the risks for participants are associated with venipuncture and immunization. Female participants will be warned about the unknown risks of the vaccine used in this study for the fetus and will be advised to use effective methods of contraception during their participation in the study, when necessary.

4.4.1 STUDY RISKS

4.4.1.1 Venipuncture

The risks associated with venipuncture include the occasional need for more than one puncture during the collection, pain and hematoma at the venipuncture site. Fainting or infection at the puncture site is rare. To minimize these risks, blood collection will be performed by trained, experienced personnel, using individual, disposable material and aseptic collection techniques.

According to Ordinance 1,353 of June 14, 2011 from the Ministry of Health, the volume of blood acceptable for collection is nine (9) mL / kg of weight for men and eight (8) m / kg of weight for women, an individual can donate blood up to three times a year. Therefore, the volume of blood collected during the study can be considered safe for the participants.

4.4.1.2 Vaccination

Possible local adverse reactions include pain, swelling, induration, itching or erythema that can last up to two to three days after vaccination, as well as inflammation in lymph nodes that drain the administration site. Systemic reactions such as pain, headache and fever in a similar way as with other vaccines can happen after using vaccines similar to those used in this study.

As with any vaccine under investigation, there is a theoretical possibility of risks that we are currently unaware of. Participants will be informed if new information becomes available or any new risks are identified. It is important to note that the study in non-human primates did not result in any safety problems related to exposure to SARS-CoV-2 after vaccination, such as, for example, any exacerbation of the disease among vaccinated animals. Thus, the possibility of an antibody-dependent exacerbation of COVID-19 remains just a theoretical risk of vaccines with no evidence yet.

4.4.2 DESCRIPTION OF ANTICIPATED BENEFITS TO THE STUDY PARTICIPANT

Participants will not receive any direct benefit from participating in this study. There is a possibility that participants will develop immunity against the SARS-CoV-2 virus that is protective against COVID-19.
It is expected that the information obtained from the present study will confirm the vaccine's efficacy and safety, enabling its registration for use by the population.

4.4.3 DESCRIPTION OF THE RELATIONSHIP BETWEEN POTENTIAL RISKS AND ANTICIPATED BENEFITS

Considering the results of similar studies that evaluated inactivated virus vaccines, Serious Adverse Events associated with the Study Vaccine are not expected to occur. Even so, the study team will actively seek to reduce the risks arising from blood collection and detect all cases of COVID-19 early to take appropriate preventive isolation measures.

Currently, COVID-19 is widely distributed, has a high incidence in Brazil and health professionals are at high risk. Therefore, efforts to find a preventive vaccine against COVID-19 represent a great potential benefit for this population.

4.4.4 DESCRIPTION OF COSTS AND REASONS FOR REFUNDS OR COMPENSATIONS TO BE USED

Participation in the study will have no cost to the participants. Participants will not receive any cash compensation for participating in this study.

Transport or parking costs to participate in scheduled visits will be reimbursed for each visit and a light snack may be offered after each blood collection.

4.5 CONFIDENTIALITY

All information related to the study will be stored securely in the research center, in files locked with a key with restricted access to study personnel. All laboratory samples, reports, administrative forms and data collection will be identified only by the number of participants' randomization in order to maintain their confidentiality.

Information that may allow identification of participant will only be accessible, within the respective research center, to members of the research team in charge of the participant's care, to clinical monitors and auditors appointed by the sponsor to supervise the activities of the center and to inspectors from regulatory authorities and ethics within the framework of current legislation.

4.6 CONFLICT OF INTEREST STATEMENT

All the principal investigators will sign a conflict of interest statement before the study begins.

The Instituto Butantan, sponsor of the research and producer of the vaccine, is a public institution of the São Paulo State Department of Health and there is no possibility of financial return for researchers or employees of the Institute in the form of royalties, shares or dividends for the sales of vaccine, if its commercialization is approved.

4.7 ACCESS TO DATA

The original databases with the information collected during the study, without the participants' identifiers, will be kept by the sponsor.

The researchers will have free access to the information collected in their respective centers during the study. Investigators will be able to request additional analyzes and / or access to the data set of all centers as long as confidentiality and the procedures provided for in the protocol are maintained.

Additional analyzes during the execution of the study that modify the objectives and endpoint of this protocol can be considered amendments and must be approved by the sponsor and the corresponding ethical and regulatory authorities.

The data will be made available at the request of the regulatory authorities within the current legal framework.

4.8 INSURANCE AND EMERGENCY CARE

In the event of adverse events associated with participation in the study, the volunteer will receive full medical care at the institution through which he was recruited. This study is supported by an insurance policy to cover losses to participants related to the use of the product under investigation under this protocol.

4.9 NOTICE TO PARTICIPANTS OF THEIR INDIVIDUAL RESULTS

Study participants will receive their validated laboratory test results as soon as they are available, including tests performed to confirm a suspected case of COVID-19. Experimental tests and those to assess the antibody response to vaccination will only be available after the blinding has broken. If

necessary, the participant may be invited to deliver the results and clarify the actions that are relevant to their health according to these results.

4.9.1 DISCLOSURE OF RESULTS TO THE PUBLIC

Full disclosure of the study will be carried out through peer-reviewed scientific publications. The decision to author the publications will follow the Uniform Requirements for Forwarding Manuscripts to Biomedical Journals of the International Committee of Medical Journal Editors available at http://www.icmje.org.

An ad hoc committee with representatives of the sponsor, the manufacturer and the researchers will be in charge of discussing the proposals for the dissemination of results. Members of this committee will write the draft manuscript of the publication and forward it to possible co-authors for contributions and approval. The sponsor must be consulted four weeks in advance of the forwarding of any results derived from this study for dissemination at a scientific event or publication. The study has been registered in a public database since before recruitment began and its results will be summarized in that same record: Record at ClinicalTrials.gov: NCT04456595. No results of the study can be released in the media without being previously presented in a peer-reviewed publication.

4.9.2 NOTICE TO PARTICIPANTS OF THE STUDY FINDINGS

All study participants will receive a summary of the main peer-reviewed scientific publication and written in appropriate language with the main research results. This summary will be subject to prior approval by the Research Ethics Committee of the institution that carries out the study. A press release will also be released with the main findings reported in this scientific publication.

4.9.3 THIRD PARTY ACCESS TO STUDY DATA

The sponsor will consider justified requests for access to the databases for additional analysis by third parties when the study is completed and the analyzes described in this protocol and the study data analysis plan are carried out. Requests may be partially accepted or total. or total. To decide on these requests, the sponsor must evaluate the protection of the confidentiality of the participants and the technical quality of the proposal.

5 REFERENCES

1. de Wilde AH, Snijder EJ, Kikkert M, van Hemert MJ. Host Factors in Coronavirus Replication. In 2017. p. 1–42. Available from: http://link.springer.com/10.1007/82_2017_25

2. Callow KA, Parry HF, Sergeant M, Tyrrell DAJ. The time course of the immune response to experimental coronavirus infection of man. Epidemiol Infect [Internet]. 1990 Oct 15;105(2):435–46. Available from: https://www.cambridge.org/core/product/identifier/S0950268800048019/type/journal_article

3. Cao W-C, Liu W, Zhang P-H, Zhang F, Richardus JH. Disappearance of Antibodies to SARS-Associated Coronavirus after Recovery. N Engl J Med [Internet]. 2007 Sep 13;357(11):1162–3. Available from: http://www.nejm.org/doi/abs/10.1056/NEJMc070348

4. Lin Q, Zhu L, Ni Z, Meng H, You L. Duration of serum neutralizing antibodies for SARS-CoV-2: Lessons from SARS-CoV infection. J Microbiol Immunol Infect [Internet]. 2020 Mar; Available from: https://linkinghub.elsevier.com/retrieve/pii/S168411822030075X

5. Payne DC, Iblan I, Rha B, Alqasrawi S, Haddadin A, Al Nsour M, et al. Persistence of Antibodies against Middle East Respiratory Syndrome Coronavirus. Emerg Infect Dis [Internet]. 2016 Oct;22(10):1824–6. Available from: http://wwwnc.cdc.gov/eid/article/22/10/16-0706_article.htm

6. Zhu N, Zhang D, Wang W, Li X, Yang B, Song J, et al. A Novel Coronavirus from Patients with Pneumonia in China, 2019. N Engl J Med [Internet]. 2020 Feb 20;382(8):727–33. Available from: http://www.nejm.org/doi/10.1056/NEJMoa2001017

7. Mousavizadeh L, Ghasemi S. Genotype and phenotype of COVID-19: Their roles in pathogenesis. J Microbiol Immunol Infect [Internet]. 2020 Mar; Available from: https://linkinghub.elsevier.com/retrieve/pii/S1684118220300827

8. Andersen KG, Rambaut A, Lipkin WI, Holmes EC, Garry RF. The proximal origin of SARS-CoV-2. Nat Med [Internet]. 2020 Apr 17;26(4):450–2. Available from: http://www.nature.com/articles/s41591-020-0820-9

9. Forster P, Forster L, Renfrew C, Forster M. Phylogenetic network analysis of SARS-CoV-2 genomes. Proc Natl Acad Sci [Internet]. 2020 Apr 28;117(17):9241–3. Available from: http://www.pnas.org/lookup/doi/10.1073/pnas.2004999117

10. van Doremalen N, Bushmaker T, Morris DH, Holbrook MG, Gamble A, Williamson BN, et al. Aerosol and Surface Stability of SARS-CoV-2 as Compared with SARS-CoV-1. N Engl J Med [Internet]. 2020 Apr 16;382(16):1564–7. Available from: http://www.nejm.org/doi/10.1056/NEJMc2004973

11. Huang C, Wang Y, Li X, Ren L, Zhao J, Hu Y, et al. Clinical features of patients infected with 2019 novel coronavirus in Wuhan, China. Lancet [Internet]. 2020 Feb;395(10223):497–506. Available from: https://linkinghub.elsevier.com/retrieve/pii/S0140673620301835

12. Chow N, Fleming-Dutra K, Gierke R, Hall A, Hughes M, Pilishvili T, et al. Preliminary Estimates of the Prevalence of Selected Underlying Health Conditions Among Patients with Coronavirus Disease 2019 — United States, February 12–March 28, 2020. MMWR Morb Mortal Wkly Rep [Internet]. 2020 Apr 3;69(13):382–6. Available from: http://www.cdc.gov/mmwr/volumes/69/wr/mm6913e2.htm?s_cid=mm6913e2_w

13. Zhu J, Ji P, Pang J, Zhong Z, Li H, He C, et al. Clinical characteristics of 3,062 COVID-19 patients: a meta-analysis. J Med Virol [Internet]. 2020 Apr 15; Available from: http://doi.wiley.com/10.1002/jmv.25884

14. Lechien JR, Chiesa-Estomba CM, De Siati DR, Horoi M, Le Bon SD, Rodriguez A, et al. Olfactory and gustatory dysfunctions as a clinical presentation of mild-to-moderate forms of the coronavirus disease (COVID-19): a multicenter European study. Eur Arch Oto-Rhino-Laryngology [Internet]. 2020 Apr 6; Available from: http://link.springer.com/10.1007/s00405-020-05965-1

15. Bialek S, Gierke R, Hughes M, McNamara LA, Pilishvili T, Skoff T. Coronavirus Disease 2019 in Children — United States, February 12–April 2, 2020. MMWR Morb Mortal Wkly Rep [Internet]. 2020 Apr 10;69(14):422–6. Available from: http://www.cdc.gov/mmwr/volumes/69/wr/mm6914e4.htm?s_cid=mm6914e4_w

16. Dong Y, Mo X, Hu Y, Qi X, Jiang F, Jiang Z, et al. Epidemiology of COVID-19 Among Children in China. Pediatrics [Internet]. 2020 Mar 16;e20200702. Available from: http://pediatrics.aappublications.org/lookup/doi/10.1542/peds.2020-0702

17. Huang L, Zhang X, Zhang X, Wei Z, Zhang L, Xu J, et al. Rapid asymptomatic transmission of COVID-19 during the incubation period demonstrating strong infectivity in a cluster of youngsters aged 16-23 years outside Wuhan and characteristics of young patients with COVID-19: A prospective contact-tracing study. J Infect [Internet]. 2020 Jun;80(6):e1–13. Available from: https://linkinghub.elsevier.com/retrieve/pii/S0163445320301171

18. Leal FE, Mendes-Correa MC, Buss LF, Costa SF, Bizario JCS, Souza SRP, et al. A primary care approach to the COVID-19 pandemic: clinical features and natural history of 2,073 suspected cases in the Corona Sao Caetano programme, Sao Paulo, Brazil. medRxiv. 2020 Jan;2020.06.23.20138081.

19. Verity R, Okell LC, Dorigatti I, Winskill P, Whittaker C, Imai N, et al. Estimates of the severity of coronavirus disease 2019: a model-based analysis. Lancet Infect Dis [Internet]. 2020 Mar; Available from: https://linkinghub.elsevier.com/retrieve/pii/S1473309920302437

20. Long Q-X, Tang X-J, Shi Q-L, Li Q, Deng H-J, Yuan J, et al. Clinical and immunological assessment of asymptomatic SARS-CoV-2 infections. Nat Med [Internet]. 2020 Jun 18; Available from: http://www.nature.com/articles/s41591-020-0965-6

21. Lin L, Lu L, Cao W, Li T. Hypothesis for potential pathogenesis of SARS-CoV-2 infection–a review of immune changes in patients with viral pneumonia. Emerg Microbes Infect [Internet]. 2020 Jan 1;9(1):727–32. Available from: https://www.tandfonline.com/doi/full/10.1080/22221751.2020.1746199

22. Lin J-T, Zhang J-S, Su N, Xu J-G, Wang N, Chen J-T, et al. Safety and immunogenicity from a phase I trial of inactivated severe acute respiratory syndrome coronavirus vaccine. Antivir Ther [Internet]. 2007;12(7):1107–13. Available from: http://www.ncbi.nlm.nih.gov/pubmed/18018769

23. Folegatti PM, Bittaye M, Flaxman A, Lopez FR, Bellamy D, Kupke A, et al. Safety and immunogenicity of a candidate Middle East respiratory syndrome coronavirus viral-vectored vaccine: a dose-escalation, open-label, non-randomised, uncontrolled, phase 1 trial. Lancet Infect Dis. 2020;

24. Thanh Le T, Andreadakis Z, Kumar A, Gómez Román R, Tollefsen S, Saville M, et al. The COVID-19 vaccine development landscape. Nat Rev Drug Discov [Internet]. 2020 May 9;19(5):305–6. Available from: http://www.nature.com/articles/d41573-020-00073-5

25. Lambert P-H, Ambrosino DM, Andersen SR, Baric RS, Black SB, Chen RT, et al. Consensus summary report for CEPI/BC March 12–13, 2020 meeting: Assessment of risk of disease enhancement with COVID-19 vaccines. Vaccine [Internet]. 2020 Jun;38(31):4783–91. Available from: https://linkinghub.elsevier.com/retrieve/pii/S0264410X2030709X

26. Development and Licensure of Vaccines to Prevent COVID-19: Guidance for Industry [Internet]. Silver Spring; 2020. Available from: www.fda.gov/media/139638/download

27. Hotez PJ, Corry DB, Strych U, Bottazzi ME. COVID-19 vaccines: neutralizing antibodies and the alum advantage. Nat Rev Immunol [Internet]. 2020 Jul 4;20(7):399–400. Available from: http://www.nature.com/articles/s41577-020-0358-6

28. Hotez PJ, Bottazzi ME, Corry DB. The potential role of Th17 immune responses in coronavirus immunopathology and vaccine-induced immune enhancement. Microbes Infect [Internet]. 2020 May;22(4–5):165–7. Available from: https://linkinghub.elsevier.com/retrieve/pii/S1286457920300721

29. Bottazzi ME, Strych U, Hotez PJ, Corry DB. Coronavirus Vaccine-Associated Lung Immunopathology-What Is The Significance? Microbes Infect [Internet]. 2020 Jun; Available from: https://linkinghub.elsevier.com/retrieve/pii/S1286457920301258

30. Gao Q, Bao L, Mao H, Wang L, Xu K, Yang M, et al. Development of an inactivated vaccine candidate for SARS-CoV-2. Science (80-) [Internet]. 2020 May 6;eabc1932. Available from: https://www.sciencemag.org/lookup/doi/10.1126/science.abc1932

31. Zhang Y-J, Zeng G, Pan H-X, Li C-G, Kan B, Hu Y-L, et al. Immunogenicity and Safety of a SARS-CoV-2 Inactivated Vaccine in Healthy Adults Aged 18-59 years: Report of the Randomized, Double-blind, and Placebo-controlled Phase 2 Clinical Trial. medRxiv [Internet]. 2020; Available from: https://www.medrxiv.org/content/early/2020/08/10/2020.07.31.20161216

32. Grifoni A, Weiskopf D, Ramirez SI, Mateus J, Dan JM, Moderbacher CR, et al. Targets of T Cell Responses to SARS-CoV-2 Coronavirus in Humans with COVID-19 Disease and Unexposed Individuals. Cell [Internet]. 2020 Jun;181(7):1489-1501.e15. Available from: https://linkinghub.elsevier.com/retrieve/pii/S0092867420306103

33. Curtis KM, Tepper NK, Jatlaoui TC, Berry-Bibee E, Horton LG, Zapata LB, et al. U.S. Medical Eligibility Criteria for Contraceptive Use, 2016. MMWR Recomm Reports [Internet]. 2016 Jul 29;65(3):1–103. Available from: http://www.cdc.gov/mmwr/volumes/65/rr/rr6503a1.htm

34. Rüggeberg JU, Gold MS, Bayas J-M, Blum MD, Bonhoeffer J, Friedlander S, et al. Anaphylaxis: Case definition and guidelines for data collection, analysis, and presentation of immunization safety data. Vaccine [Internet]. 2007 Aug;25(31):5675–84. Available from: https://linkinghub.elsevier.com/retrieve/pii/S0264410X07002642

35. Hanley JA, Lippman Hand A. If Nothing Goes Wrong, Is Everything All Right?: Interpreting Zero Numerators. JAMA J Am Med Assoc. 1983;

36. Brasil M da S. Diretrizes Operacionais para o Estabelecimento e o Funcionamento de Comitês de Monitoramento de Dados e de Segurança. 2008.

37. ICH. Clinical Safety Data management: Definitions and Standards for Expedited Reporting [Internet]. Genebra: International Conference on Harmonisation of Technical Requirements ror Registration of Pharmaceuticals for Human Use; 1994 [citado 2011 Set 29]. E2A .

38. Agência Nacional de Vigilância Sanitária. Gerência de Farmacovigilância, Ministério da Saúde B. Guias de farmacovigilância para detentores de registro de Medicamentos. Ministério da Saúde Bras{$\$'$\$i}lia; 2010.

39. UMC/WHO. The use of the WHO-UMC system for standardised case causality assessment [Internet]. Uppsala:The Uppsala Monitoring Centre. [citado 2011 Set 29]. Disponível em: http://www.who-umc.org/Graphics/24734.pdf.

40. Law B, Sturkenboom M. D2.3 Priority List of Adverse Events of Special Interest: COVID-19 [Internet]. Decatur, GA; 2020. Available from: brightoncollaboration.us/wp-content/uploads/2020/06/SPEAC_D2.3_V2.0_COVID-19_20200525_public.pdf

41. Marshall JC, Murthy S, Diaz J, Adhikari NK, Angus DC, Arabi YM, et al. A minimal common outcome measure set for COVID-19 clinical research. Lancet Infect Dis [Internet]. 2020 Aug;20(8):e192–7. Available from: https://linkinghub.elsevier.com/retrieve/pii/S1473309920304837

42. Ministério da Saúde, Conselho Nacional de Saúde. Resolução no 466, de 12 de dezembro de 2012, que trata sobre as diretrizes e normas regulamentadoras de pesquisas envolvendo seres humanos. Diário Of da União. 2012;

42. Brasil. Ministério da Saúde. Conselho Nacional de Saúde. Resolução CNS Nº 441. 2011.

43. CNS/MS. Resolução CNS Nº 441 [Internet]. Brasília: Conselho Nacional de Saúde; 2011 [citado 2011 Set 29]. Disponível em: http://conselho.saude.gov.br/resolucoes/2011/Reso441.pdf.

44. Ministério da Saúde B, ANVISA. RDC 09/2015.In: Agência Nacional de Vigilância Sanitária editor. Brasília DF. 114. 2011.
